# Light-powered *Escherichia coli* cell division for chemical production

**DOI:** 10.1038/s41467-020-16154-3

**Published:** 2020-05-08

**Authors:** Qiang Ding, Danlei Ma, Gao-Qiang Liu, Yang Li, Liang Guo, Cong Gao, Guipeng Hu, Chao Ye, Jia Liu, Liming Liu, Xiulai Chen

**Affiliations:** 1 State Key Laboratory of Food Science and Technology, Jiangnan University, 214122 Wuxi, China; 2 Key Laboratory of Industrial Biotechnology, Ministry of Education, Jiangnan University, 214122 Wuxi, China; 3 Hunan Provincial Key Laboratory for Forestry Biotechnology, Central South University of Forestry and Technology, 410004 Changsha, China; 4 National Engineering Laboratory for Cereal Fermentation Technology, Jiangnan University, 214122 Wuxi, China

**Keywords:** Metabolic engineering, Metabolic engineering, Metabolic engineering

## Abstract

Cell division can perturb the metabolic performance of industrial microbes. The C period of cell division starts from the initiation to the termination of DNA replication, whereas the D period is the bacterial division process. Here, we first shorten the C and D periods of *E. coli* by controlling the expression of the ribonucleotide reductase NrdAB and division proteins FtsZA through blue light and near-infrared light activation, respectively. It increases the specific surface area to 3.7 μm^−1^ and acetoin titer to 67.2 g·L^−1^. Next, we prolong the C and D periods of *E. coli* by regulating the expression of the ribonucleotide reductase NrdA and division protein inhibitor SulA through blue light activation-repression and near-infrared (NIR) light activation, respectively. It improves the cell volume to 52.6 μm^3^ and poly(lactate-co-3-hydroxybutyrate) titer to 14.31 g·L^−1^. Thus, the optogenetic-based cell division regulation strategy can improve the efficiency of microbial cell factories.

## Abstract (web summary)

Manipulation of genes controlling microbial shapes can affect bio-production. Here, the authors employ an optogenetic method to realize dynamic morphological engineering of *E. coli* replication and division and show the increased production of acetoin and poly(lactate-co-3-hydroxybutyrate).

## Introduction

Microbial cell factories offer an economic and environmentally friendly method for producing valuable chemicals, including biofuels, fine chemicals, and pharmaceuticals, from renewable feedstock^1,2^. To maximize the efficiency of microbial cell factories, a series of strategies have been developed, including traditional breeding^3^, adaptive evolution^4^, synthetic biology^5,6^, and metabolic engineering^4,7^. Among these strategies, metabolic engineering has proven to be the most efficient and feasible way in improving the performance of industrial microbes^4,8,9^. Recently, DCEO biotechnology^10^ is proposed for engineering industrial microbes, and mainly involves in four technical steps: pathway design, pathway construction, pathway evaluation, and pathway optimization. Based on this, metabolic engineering strategies can be adopted to engineer microbial cell factories for chemical production at DNA level (promoter engineering^11^), RNA level (transcription factor engineering and synthetic RNA switches^12^), protein level (protein engineering and cofactor engineering^5^), and cell level (morphology engineering^13^ and consortia engineering^14^). In these strategies, cell morphology can affect the efficiency of microbial cell factories at cell level by changing cell density^15^, mass transfer^16^, cell size^13,16^, and cell lysis^17,18^.

Cell morphology can be generally affected by culture conditions^19^, nutrient components^20^, cell division^21^, and cell metabolism^22–24^. Previous studies on regulating cell morphology are mainly divided into three aspects: (1) Nutritional regulation: carbon sources, nitrogen sources, and metal ions can be used to regulate cell growth and biomass density by supplying essential components^15^; (2) Mechanical regulation: micro-particle cultivation and fermenter pressure play important roles in changing the culture rheology and oxygen transfer rate by affecting nutrient assimilation and mass transfer^15,19^; and (3) Skeleton protein regulation: skeleton proteins can alter cell width and cell lysis by perturbing cell membrane synthesis^25^. On the other hand, bacteria can tightly coordinate various events during cell cycle to control their cell morphology^26,27^. There are two key stages in the bacterial cell cycle: the C period starts from the initiation to the termination of DNA replication, and the D period is the bacterial division process^21^. The C and D periods of cell division can affect cell morphology by perturbing dNTP synthesis or the assembly of *Escherichia coli* divisomes^21,28^. As a result, cell growth or cell volume is efficiently improved for chemical production^13,29,30^. Previous studies have demonstrated that overexpression of the division-related proteins—FtsZ, FtsQ, FtsA, and FtsN—or ribonucleotide reductases—NrdA, NrdB, and NrdD—can shorten the C and D periods. In addition, weak expression of NrdA, NrdB, and NrdD or overexpression of the division protein inhibitors of SulA, MinC, MinD, MinE, and FtsH can prolong the C and D periods^30–32^ (Supplementary Note 1). Thus, how to change the cell morphology by regulating the C and D periods is interesting for chemical production. Optogenetics is an efficient strategy for regulating cell phenotypes to enhance the chemical production at multiple levels in a noninvasive, reversible, and spatiotemporal manner. For example, blue light-mediated optogenetics, far-red light-regulated CRISPR technology, and light-based metabolic flux regulation have been used for isobutanol production^33^, live cell therapy^34^, and deoxyviolacein biosynthesis^35^, respectively. These studies indicate that the optogenetics-based cell division may be a promising strategy for improving the efficiency of chemical production.

In this study, we show that the C and D periods of cell division can be shortened or prolonged through optogenetic regulation. The specific surface area (SSA) of *E. coli* is engineered to drive acetoin production by shortening the C and D period with a blue light activation and near-infrared (NIR) light activation system (BANA). The cell volume of *E. coli* is engineered to increase poly(lactate-co-3-hydroxybutyrate) production by prolonging the C and D periods with a blue light activation–repression and NIR light activation system (BARNA). The optogenetics-based cell division strategy can increase chemical production via microbial cell factories.

## Results

### Screening cell division genes

The 14 genes involved in the C and D periods of cell division were divided into two distinct groups. The first group included nine overexpressed genes (*nrdAB*, *nrdA*, *nrdB*, *nrdD*, *ftsZA*, *ftsZ, ftsA*, *ftsQ*, and *ftsN*), which can stimulate dNTP synthesis and *E. coli* divisome assembly^21^. Genes in the second group included the weakly expressed *nrdAB*, *nrdA*, *nrdB*, and *nrdD* and overexpressed *sulA, minC, minD, minE*, and *ftsH*. These genes can perturb dNTP synthesis and Z-ring assembly^36,37^, thus are involved in prolonging cell division.

**Fig. 1:**
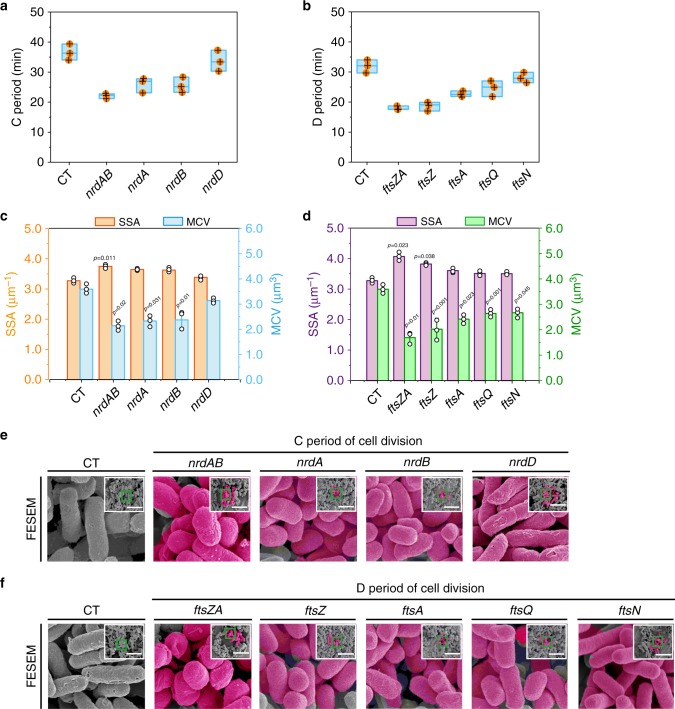
Shortening the C and D periods of cell division by genetic manipulation. **a**, **b** Effect of shortening the C and D periods of cell division on cell division. **c**, **d** Effect of shortening the C and D periods of cell division on the specific surface area (SSA) and mean cell volume (MCV), respectively. **e**, **f** Confirming the morphological variations of all engineered strains by the filed emission scanning electron microscopy (FESEM). The pink color of FESEM was highlighted. The original pictures were shrinked into the top right corner of the enlarged pictures. **a**, **b** The box plots define the minima, maxima, center, and bounds of box. **e**, **f** The scale bar is 5 μm. Significance (*p*-value) was evaluated by two-sided *t*-test compared to CT. **a**–**d** Values were shown as mean ± s.d. from three (*n* = 3) biological independent replicates. Information for each gene was provided in Supplementary Note 1. Source data are provided as a Source data file.

As illustrated in Fig. 1, independent overexpression of *nrdAB*, *nrdA*, *nrdB*, *nrdD, ftsZA, ftsZ*, *ftsA*, *ftsQ*, and *ftsN* in *E. coli* JM109 shortened cell division (Supplementary Data 1 and 2). As a result, the periods of cell division, mean cell length (MCL), mean surface area, and mean cell volume (MCV) were decreased. The SSA, cell count (c.f.u), cell growth (OD_600_), and dry cell weight (DCW) were increased. In addition, the mean cell width (MCW) was not changed (Fig. 1e, f, Supplementary Figs. 1A, B and  4A). However, when *nrdAB* (or *ftsZA*) was overexpressed, an obvious difference was observed in cell morphology. Compared with the control strain, the periods of cell division, mean surface area, MCV of the engineered strains overexpressing *nrdAB* (or *ftsZA*) were decreased by 39.73% (or 43.75%), 31.66% (or 41.53%), and 40.35% (or 52.94%), respectively (Fig. 1a–d, Supplementary Fig. 3A). In addition, the SSA, c.f.u, OD_600_, and DCW were increased by 14.69% (or 24.25%), 15.73% (or 25.84%), 38.98% (or 37.29%), and 38.98% (or 37.29%), respectively (Fig. 1c, d, Supplementary Figs. 5A and  7A). These results indicated that overexpressing *nrdAB* and *ftsZA* could efficiently shorten the C and D periods of cell division, respectively.

**Fig. 2:**
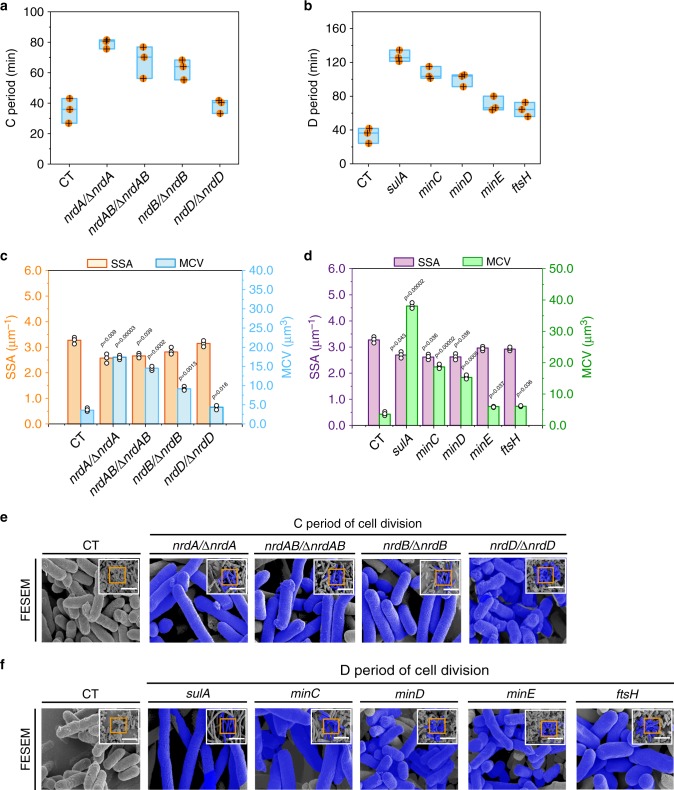
Prolonging the C and D periods of cell division by genetic manipulation. **a**, **b** Effect of prolonging the C and D periods of cell division on cell division. **c**, **d** Effect of prolonging the C and D periods of cell division on the specific surface area (SSA) and mean cell volume (MCV). **e**, **f** Confirming the morphological variations of the all engineered strains by the FESEM. The blue color of FESEM was highlighted. For the original pictures were shrinked into the top right corner of the enlarged pictures. In *nrdA/*∆*nrdA*, *nrdAB/*∆*nrdAB*, *nrdB*/∆*nrdB*, and *nrdD/*∆*nrdD* mutants, *nrdA*, *nrdAB*, *nrdB*, and *nrdD* were weakly expressed by low concentration IPTG (10 μM), respectively. **a**, **b** The box plots define the minima, maxima, center, and bounds of box. **e**, **f** The scale bar is 5 μm. Significance (*p*-value) was evaluated by two-sided *t*-test, compared to CT. **a**–**d** Values are shown as mean ± s.d. from three (*n* = 3) biological independent replicates. Information for each gene was provided in Supplementary Note 1. Source data are provided as a Source data file.

As showed in Fig. 2, the independent weak expression of *nrdAB*, *nrdA*, *nrdB*, and *nrdD* in the corresponding *E. coli* JM109 mutant strains and the independent overexpression of *sulA, minC, minD, minE*, and *ftsH* in *E. coli* JM109 could prolong cell division (Supplementary Data 1 and 2). As a result, the periods of cell division, the MCL, mean surface area, and MCV were increased. The SSA, c.f.u, OD_600_, and DCW were decreased. In addition, those genes expression did not change MCW (Fig. 2e, f, Supplementary Figs. 2A, B,  4B). Among these strains, an obvious difference was showed in cell morphology by weak expression of *nrdA* and overexpression of *sulA*. Compared with the control strain, the periods of cell division, mean surface area, MCV of the engineered strains with weak expression of *nrdA* (or overexpression of *sulA*) were increased by 124.6% (or 271.8%), 281.21% (or 776.87%), and 384.57% (or 960.62%), respectively (Fig. 2a–d, Supplementary Fig. 3B). In addition, the SSA, c.f.u, OD_600_, and DCW were decreased by 21.41% (or 17.74%), 11.55% (or 31.05%), 4.65% (or 5.81%), and 4.65% (or 5.81%), respectively (Fig. 2c, d, Supplementary Fig. 6A,  7B). Compared with *E. coli* JM109 with weak expression of *nrdA*, *nrdAB*, *nrdB*, and *nrdD*, the C period of *E. coli* JM109 with deletion of *nrdA*, *nrdAB*, *nrdB*, and *nrdD* was increased by 102.77%, 51.58%, 59.28%, and 18.53%, respectively (Supplementary Fig. 3C, and Supplementary Note 2). These results suggested that weak expression of *nrdA* and overexpression of *sulA* could efficiently prolong the C and D periods of cell division, respectively.

**Fig. 3:**
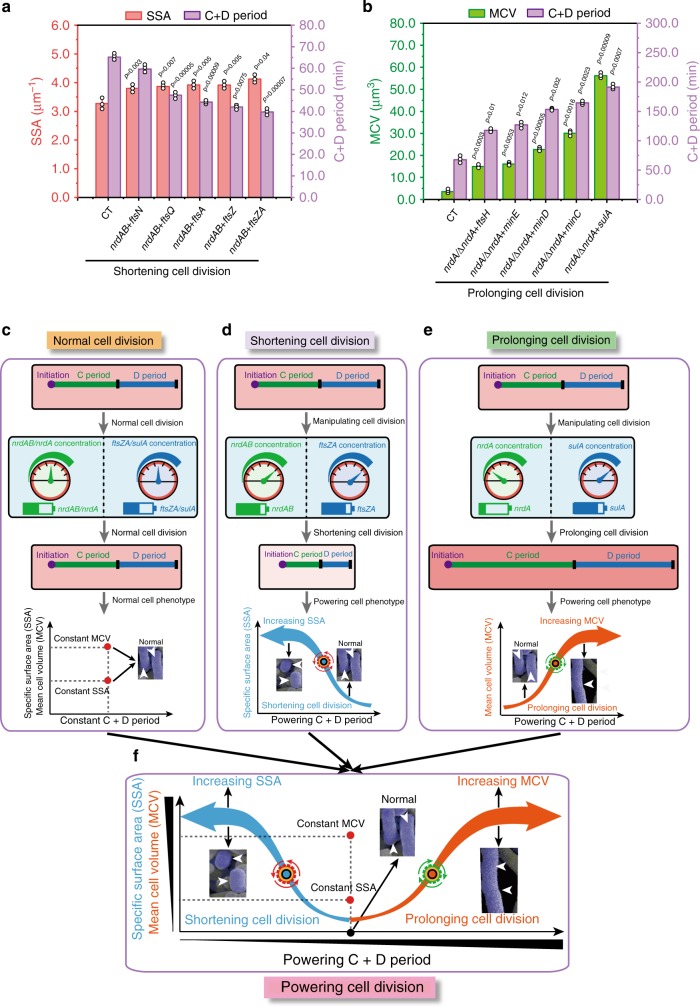
Combination of the C and D periods of cell division. **a** Combining the C and D periods for shortening cell division. **b** Combining the C and D periods for prolonging cell division. **c** Effect of normal cell division on cell morphology and the C+D periods. **d** Effect of shortening cell division on cell morphology and the C+D periods. **e** Effect of prolonging cell division on cell morphology and the C+D periods. **f** Engineering the C+D periods of cell division for improving specific surface area (SSA) or mean cell volume (MCV). Significance (*p*-value) was evaluated by two-sided *t*-test, compared to CT. **a**, **b** Values are shown as mean ± s.d. from three (*n* = 3) biological independent replicates. Source data underlying Fig. 3a, b are provided as a Source data file.

Finally, we investigated the effect of *nrdAB* and *ftsZA* overexpression on shortening cell division and the effect of weak *nrdA* expression and *sulA* overexpression on prolonging cell division. As shown in Fig. 3a, b, the simultaneous overexpression of *nrdAB* and *ftsZA* increased the SSA to 4.12 μm^−1^, and the weak expression of *nrdA* and overexpression of *sulA* increased the MCV to 56.2 μm^3^. However, the coexpression of *nrdAB* + *ftsZAQ* and *nrdAB* + *ftsZAQN* did not shorten the C and D periods or increase the SSA, c.f.u, or OD_600_ (Supplementary Fig. 8A, B,  9A). Thus, the *nrdAB* + *ftsZA* and *nrdA* + *sulA* groups were chosen to shorten or prolong the C and D periods of cell division, respectively (Fig. 3c–f).

### Constructing the basic tools for powering cell division

To achieve spatial, temporal, and reversible control of *E. coli* SSA and MCV, three basic optogenetics-based cell division regulation tools, a blue-light activation tool (BLAT), a blue-light repression tool (BLRT), and an NIR-light activation tool (NRAT), were designed and constructed (Supplementary Fig. 13).

**Fig. 4:**
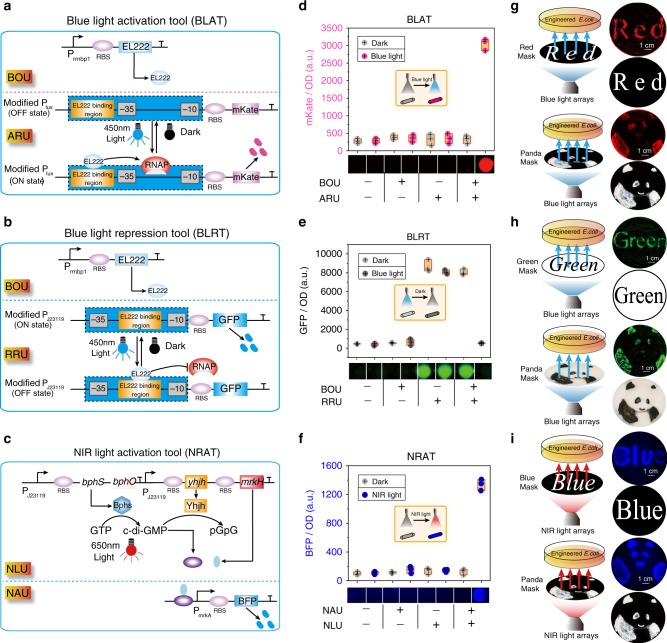
The performance of three basic optogenetics tools. **a**–**c** The schematic diagram of BLAT (blue-light), BLRT (blue-light), and NRAT (NIR-light), respectively. **d**–**f** Fluorescence activation with BLAT, BLRT, and NRAT under dark, 0.8 W/cm^2^ blue-light, and NIR-light, respectively. **g**–**i** Blue-light or NIR-light LEDs were mounted on the bottom of a photomask with a setup for pattern illumination. Red, green, blue, and chinese panda-patterned masks made from aluminum foil were placed on top of the photomask to illuminate culture plate. In this setup, photomasks were placed onto the bottom of the engineered *E. coli*-culture plate with BLAT, BLRT, and NRAT, respectively. *P*_J23119_: constitutive promoter; *P*_lux_: quorum sensing promoter; *P*_mrkA_: MrkH-targeted promoter; EL222: light-sensitive protein from *Erythrobacter litoralis*; *bphS*: a c-di-GMP diguanylate cyclase from *Rhodobacter sphaeroides*; *bphO*: a heme oxygenase from *Rhodobacter sphaeroides*; *yhjH*: c-di-GMP PDE from *E. coli*; *mrkH*: a transcriptional factor from *Klebsiella pneumoniae*. The box plots define the minima, maxima, center, and bounds of box. **d**–**f** *n* values are shown as mean ± s.d. from three (*n* = 3) biological independent replicates. Source data underlying Fig. 4d–i are provided as a Source data file.

To construct a BLAT, two units were designed and assembled (Fig. 4a): a blue optogenetics unit (BOU) to express light-sensitive protein EL222, and an activation reporter unit to replace the LuxR-binding region with an EL222-binding region by overlaping the −35 region (*E. coli* consensus −35 and −10 regions) of the LuxI promoter. In this BLAT, EL222 can bind to the blue-light-inducible promoter following illumination with blue light, and then induce gene expression by recruiting RNA polymerase. To construct a BLRT, the modified J23119 promoter was converted into a transcriptional repressor by positioning the J23119 box between and partially overlapping the consensus −35 and −10 regions of the constitutive promoter^38^, resulting in an repression reporter unit (Fig. 4b), which was then combined with the blue optogenetics unit (BOU). To construct an NRAT, two units were designed and assembled: a near-infrared light unit to constitutively express the *bphS*, *bphO, yhjH*, *mrkH* genes, and a near-infrared activation unit with the *P*_mrkA_ promoter (Fig. 4C). In this NRAT, GTP is converted into c-di-GMP by utilizing the BphS and BphO proteins, and then c-di-GMP can be degraded by the YhjH protein. Finally, the MrkH protein can bind c-di-GMP to activate the MrkH-dependent *P*_mrkA_ promoter^39^.

The performances of BLAT, BLRT, and NRAT were evaluated. The abundance of mKate (BLAT) and BFP (NRAT) was increased by 9.3-fold and 10.5-fold with illumination of 0.8 W/cm^2^ blue light (Fig. 4d) and 0.8 W/cm^2^ NIR light (Fig. 4f), respectively, but the GFP abundance (BLRT) was decreased by 15.2-fold with illumination with 0.8 W/cm^2^ blue light (Fig. 4e) compared to the corresponding abundance of the dark condition. The genes of interest (GOIs) of BLAT and NRAT showed an increased abundance, but the abundance of the BLRT GOIs decreased with the increasing illumination time (0–14 h), intensity (0–0.8 W/cm^2^), and pulse (0–100%) (Supplementary Figs. 14A–C,  17A–C, and  20A–C). Then, the bioimaging in Fig. 4g–i for red, green, blue, and panda with a setup were used to display the spatial specificity of BLAT, BLRT, and NRAT. For the temporal specificity of BLAT, BLRT, and NRAT, the reversible transcription activation in β-galactosidase or β-glucuronidase was presented by an ON-OFF-ON or OFF-ON-OFF switch under BLAT, BLRT, or NRAT control, respectively (Supplementary Figs. 15A, B,  18A, B, and  20D).

To further obtain a powerful light-inducible tool, we optimized BLAT, BLRT, and NRAT, respectively. In BLAT, the EL222-binding site length and EL222 expression level were modified to control the mKate abundance, and thus the results showed a 23.5-fold activation in mKate abundance (Supplementary Fig. 16A–C). In BLRT, a promoter library of the −35 and −10 hexamers was constructed and screened to tune the dynamic range of the bacterial promoters, and thus the results revealed a 53-fold repression in GFP expression of aB (TTGACA/GATAAT) group (Supplementary Fig. 19A, B). In NRAT, the RBS strength of *yhjH* was optimized to regulate the c-di-GMP levels, and thus the results showed a 28.6-fold increase in BFP abundance (Supplementary Fig. 21A). Additionally, these three optogenetic tools were noninvasive for cell growth under blue or NIR light (Supplementary Fig. 22A–C).

The optogenetic tools could efficiently regulate endogenous bacterial genes. BLAT, BLRT, and NRAT were introduced into *E. coli* JM109 to replace the fluorescent proteins and spatiotemporally control cell division. Compared to the control strain, when BLAT was used to shorten the C+D periods of cell division, the SSA was increased by 1.26-fold (Supplementary Fig. 23A). In addition, when BLAT was used to prolong the C and D periods of cell division, the MCV was increased by 3.68-fold and 10.36-fold, respectively (Supplementary Fig. 23A). Similarly, when BLRT and NRAT were introduced into *E. coli* JM109, the C and D periods of cell division were increased and decreased, respectively (Supplementary Fig. 23B, C). To further confirm the universal properties of these optogenetic tools, BLAT, BLRT, and NRAT were introduced into lactate-producing strain *E. coli* GL0002^40^ and pyruvate-producing strain *E. coli* F0601^40^, the similar changes in the C and D period of cell division were also detected, respectively (Supplementary Fig. 24A). Together, these results demonstrated that the optogenetic tools exhibited good applicability for the regulation of cell division.

### Enhancing acetoin production by shortening cell division

**Fig. 5:**
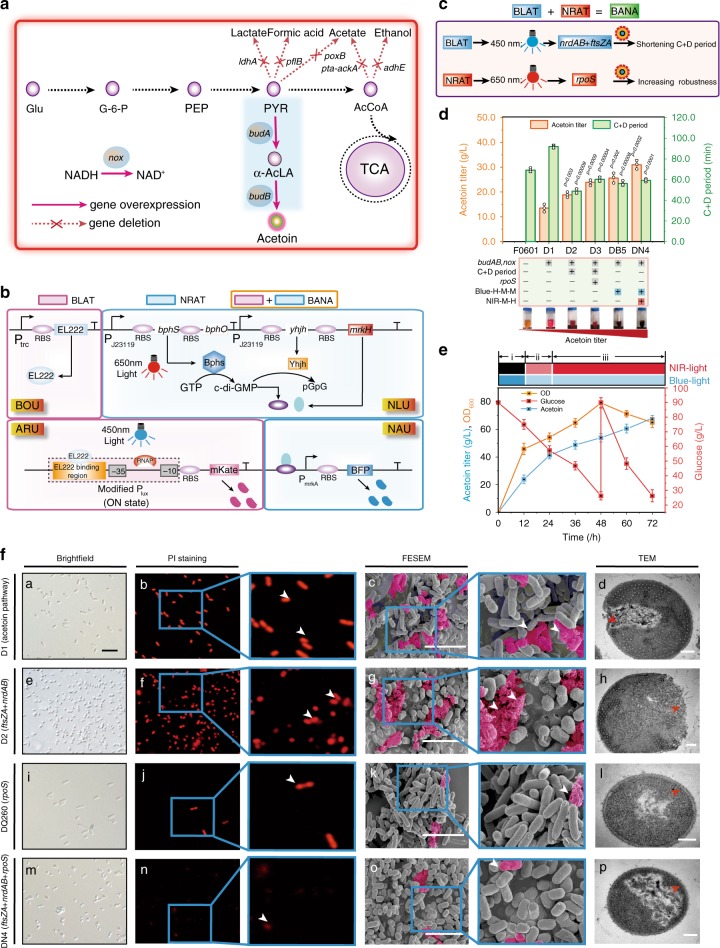
Shortening cell division for acetoin production by the BANA system. **a** The schematic diagram of acetoin biosynthesis pathway in *E. coli* D1. **b** The schematic diagram of BANA containing the BLAT and NRAT. **c** Enhancing acetoin production by BANA regulation. BLAT was used to control *nrdAB* and *ftsZA*, and at the same time NRAT controlled *rpoS*. **d** Effect of illumination combination on the C+D periods and acetoin production. H, M, and L were 0.8, 0.3, and 0.2 W/cm^2^, respectively (Supplementary Fig. 27A–C). The genes in the C+D periods of cell division were constitutively expressed in *E. coli* D1, resulting in *E. coli* D2. The *rpoS* gene was constitutively expressed in *E. coli* D2, resulting in *E. coli* D3. The genes in the C+D periods of cell division were controlled by blue light in *E. coli* D1, resulting in *E. coli* DB5 (Blue-H-M-M represented 0.8 W/cm^2^ blue-light in phase I and 0.3 W/cm^2^ blue-light in phase II and phase III). The *rpoS* gene was controlled by NIR-light in *E. coli* DB5, resulting in *E. coli* DN4 (NIR-M-H represented 0.3 W/cm^2^ NIR-light in phase II and 0.8 W/cm^2^ NIR-light in phase III (Supplementary Fig. 30A, B, and Supplementary Note 6). The inserted figure was the creatine color reaction. **e** Acetoin production with the engineered *E. coli* DN4 controlled by light stimulation during fed-batch fermentation. **f** Effect of shortening cell division on cell morphology during acetoin production. The *rpoS* gene was constitutively expressed in *E. coli* D1, resulting in *E. coli* DQ260. The scale bar for c, g, k, o are 5 μm. For d, h, l, p are 0.2 μm. For a, e, i, m are 5 μm. **d**, **e** Values are shown as mean ± s.d. for *n* = 3 biological independent replicates. Glu glucose, G6P glucose-6-phosphate, PYR pyruvate, PEP phosphoenolpyruvate, AcCoA acetyl-CoA, α-acLA α-acetolactate, α-*ackA* acetate kinase A, *adhE* alcohol dehydrogenase, *ldhA* lactate dehydrogenase, *poxB* pyruvate oxidase, *pflB* pyruvate-formate lyase; *pta* phosphate acetyl transferase, *budA* α-acetolactate synthase, *budB* α-acetolactate decarboxylase, *nox* NADH oxidase. Significance (*p*-value) was evaluated by two-sided *t*-test, compared to *E. coli* D1. Source data underlying Fig. 5d–f are provided as a Source data file.

Acetoin, a food flavoring and fragrance, is widely used in the food, pharmaceutical, and chemical industries^41^. An engineered *E. coli* D1 strain was constructed by constitutively overexpressing *budA*, *budB*, and *nox* genes in the biosynthetic pathway of acetoin. Based on this, the engineered strain produced 13.5 g L^−1^ acetoin (Fig. 5A, D, Supplementary Data 1 and 2).

Then, the state of cell division and robustness of engineered *E. coli* D1 were analyzed. As shown in Fig. 5D and Supplementary Fig. 28B, it was found that the C+D period of cell division (*E. coli* D1) was prolonged by 33.09% compared to *E. coli* F0601. As a result, the SSA and relative cell viability of *E. coli* D1 were decreased by 6.89% and 50.5%, respectively. These results demonstrated that prolonging the C+D period of cell division decreased SSA, and then disturbed cell viability, thus leading to the cessation of cell growth and potentially affecting acetoin production. To enhance cell growth and improve strain robustness, the process of acetoin fermentation was divided into three phases by introducing the optogenetic strategies to shorten cell division: (1) shortening phase to shorten cell division and increase the SSA by high-level expression of *nrdAB* + *ftsZA*; (2) cooperating phase to balance cell growth and acetoin production by expressing *nrdAB* + *ftsZA* at a moderate level and stimulating the expression of the *rpoS*^42^; and (3) defensing phase to improve strain robustness by expressing *nrdAB* + *ftsZA* at a moderate level and *rpoS* at a high level (Fig. 5c, Supplementary Fig. 25A, B, Supplementary Note 3, and Supplementary Data 4).

To spatiotemporally tune these three phases, a blue-light activation and NIR-light activation system (BANA) was constructed by combining BLAT and NRAT (Fig. 5b). Then, we confirmed the similar functions of dose-dependent and spatiotemporal specificity of BANA by implementing the illumination intensity, pulse, bioimaging, and GOIs abundance experiments, respectively (Supplementary Fig. 26A–C). Further, BANA was introduced into *E. coli* D1 to increase acetoin production by spatiotemporally regulating the expression of *nrdAB, ftsZA*, and *rpoS*. Based on this, the aforementioned three phases were achieved by manipulating the intensity and time of blue-light and NIR-light illumination, respectively (Fig. 5c, Supplementary Fig. 27A–C). This manipulation led to the formation of *E. coli* DN4. As a result, the C+D period of *E. coli* DN4 was shortened by 35.56% compared to *E. coli* D1, whereas the SSA, relative cell viability and half-maximal inhibitory concentration were increased by 20.39%, 62.18%, and 46.23%, respectively (Fig. 5d, f, Supplementary Fig. 28A, B). These results led to a 131.1% increase in the titer and productivity of acetoin in *E. coli* DN4 compared to those of *E. coli* D1, respectively (Fig. 5d). When this culture was scaled up to a 5-L fermenter, the acetoin titer, productivity, and OD_600_ of *E. coli* DN4 under light conditions were increased to 67.2 g L^−1^, 0.93 g L^−1^ h^−1^, and 64.97, respectively, which were 39.19, 39.19, and 16.43% higher than that of *E. coli* D1, 15.17, 15.17, and 6.02% higher than that of *E. coli* D3, 41.5, 41.5, and 23.78% higher than that of *E. coli* DN4 under the dark condition, respectively (Fig. 5e, Table 1). Furthermore, based on the fluorescence intensity and fluorescence density percentage under 5 and 10 L fermenters, a similar fluorescence expression in 250 mL shake flasks was activated. These results indicated the fine applicability of scale and high-density fermentation (Supplementary Figs. 31–33, and Supplementary Note 4).

**Table 1:** Differences in wild type and morphology engineered *E. coli* on production of acetoin in 5 L fermenter.

Chemical	Strains characteristics	Titer (g L^−1^)	Productivity (g L^−1^ h^−1^)	Cell growth (OD_600_)
Acetoin	*E. coli* D1 (Acetoin pathway)	48.28	0.67	55.8
	*E. coli* D3 (Acetoin pathway + C and D period, *rpoS*)	58.35 (*p* = 0.019)	0.81 (*p* = 0.019)	61.28 (*p* = 0.05)
	*E. coli* DN4 in the dark condition (Acetoin pathway + BANA-controlled C and D period, *rpoS*)	47.49	0.66	52.49
	*E. coli* DN4 in the light condition (Acetoin pathway + BANA-controlled C and D period, *rpoS*)	67.2 (*p* = 0.011)	0.93 (*p* = 0.011)	64.97 (*p* = 0.022)

Significance (*p*-value) was relative to the control strain *E. coli* D1; Differences were determined by two-tailed Student’s *t*-test.

### Increasing poly(lactate-co-3-hydroxybutyrate) (PLH) production by prolonging cell division

**Fig. 6:**
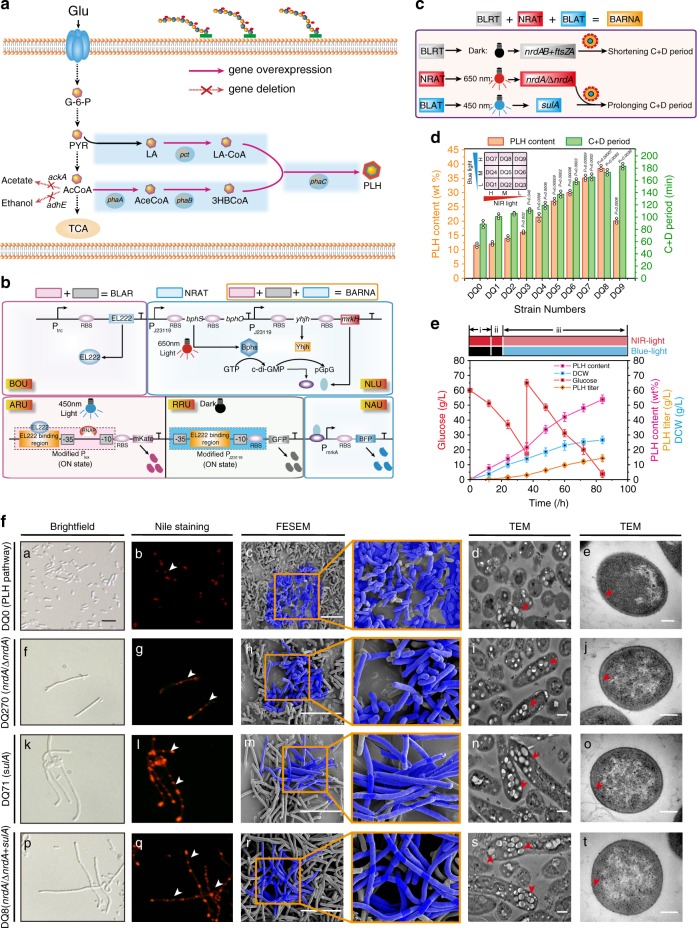
Prolonging cell division for PLH production by the BARNA system. **a** The schematic diagram of PLH biosynthesis pathway in *E. coli* DQ0. **b** The schematic diagram of BARNA containing BLRT and BANA. **c** Increasing PLH production by BARNA regulation. BLRT, BLAT, and NRAT were used to control *nrdAB* + *ftsZA*, *sulA*, and *nrdA*, respectively. **d** Effect of blue-light and NIR-light combination on PLH content and the C+D periods. H, M, and L were 0.8, 0.3, and 0.2 W/cm^2^, respectively (Supplementary Figs. 37A, B,  38A, B). The inserted figure was nine combination group (*E. coli* DQ1–DQ9) with orthogonally matrix by blue and NIR-light illumination intensity. **e** PLH production with the engineered *E. coli* DQ8 controlled by light stimulation during fed-batch fermentation. Phase I, II, and III were controlled by 0.8 W/cm^2^ NIR-light, 0.8 W/cm^2^ NIR-light, 0.8 W/cm^2^ blue-light and 0.3 W/cm^2^ NIR-light, respectively. **f** Effect of prolonging cell division on cell morphology during PLH production. The *nrdA* and *sulA* genes were constitutively expressed in *E. coli* DQ0, resulting in *E. coli* DQ270 and *E. coli* DQ271, respectively. The scale bar for c, h, m, r is 10 μm. The scale bar for d, i, n, and s is 0.5 μm. For e, j, o, and t is 0.2 μm. For a, f, k, and p is 5 μm. **d**, **e** values are shown as mean ± s.d. from three (*n* = 3) biological independent replicates. Glu glucose, G6P glucose 6-phosphate, PYR pyruvate, LA lactate, PEP phosphoenolpyruvate, AcCoA acetyl-CoA, aceCoA acetoacetyl-CoA, 3HBCoA 3-hydroxybutyryl-CoA, LAcoA lactyl-coA, *ackA* acetate kinase A, *adhE* alcohol dehydrogenase, *phaA* β-ketothiolase, *phaB* acetoacetyl-CoA reductase, *phaC* PHA synthase, *pct* propionyl-CoA transferase. Significance (*p*-value) was evaluated by two-sided *t*-test, compared to *E. coli* DQ0. Source data underlying Fig. 6d–f are provided as a Source data file.

PLH is a biodegradable and biocompatible synthetic polymer that accumulates as inclusion bodies in microorganisms^43^. We engineered *E. coli* DQ0, in which the *phaA, phaB, phaC*, and *pct* genes involved in the PLH pathway were constitutively overexpressed. Analytical result showed that its PLH content was at 11.5 wt% level (Fig. 6a, d, Supplementary Data 1 and 2). Then, the state of cell division were analyzed (Supplementary Fig. 34A, B). We found that the C+D period of cell division (*E. coli* DQ0) was prolonged by 17.95% (Fig. 6d). As a result, the MCV was increased by 9.38%, but c.f.u of *E. coli* DQ0 was decreased by 8.9% compared to that of *E. coli* GL0002 (Fig. 6d, Supplementary Fig. 39A, C). These results indicated that PLH production was limited by the MCV and cell counts. To solve these limitations in PLH fermentation, the process of PLH biosynthesis was divided into three phases by introducing the optogenetic strategies to prolong cell division: (1) shortening cell division to increase the cell count by the expression of *nrdAB, ftsZA*, and *nrdA*; (2) switching time phase to increase the PLH content by optimizing the switch time from dark to blue light; and (3) prolonging cell division to increase the MCV by stimulating the expression of *sulA* and decreasing the expression of *nrdA* (Fig. 6c, Supplementary Fig. 34A, B).

To spatiotemporally regulate these three phases, a blue light activation-repression and NIR light activation system (BARNA) was constructed by combining BANA and BLRT (Fig. 6b). Then, we confirmed the similar functions of dose-dependent and spatiotemporal specificity of BARNA by conducting the illumination pulse, intensity, bio-imaging, and GOIs abundance experiments (Supplementary Fig. 35A,  36A, B). Furthermore, BARNA was introduced into *E. coli* DQ0 to enhance PLH production by spatiotemporally controlling the expression of *nrdAB*, *ftsZA*, *nrdA*, and *sulA* genes. Based on this, the aforementioned three phases were achieved by adjusting the illumination intensity and time (Fig. 6c, Supplementary Fig. 37A, B, and Supplementary 38A, B). This manipulation led to the formation of *E. coli* DQ8. As a result, compared to *E. coli* DQ0, the C+D period of *E. coli* DQ8 was prolonged by 1.96-fold, whereas the cell wall thickness was decreased by 18.6% (Fig. 6D, F). In addition, the MCV and c.f.u were increased by 13.65-fold and 1.16-fold, respectively (Supplementary Fig. 39A, C). These results led to the PLH content, DCW, and PLH titer increased to 38.5 wt%, 7.41 g L^−1^, and 2.85 g L^−1^, respectively, which was 234.78, 36.96, 359.68% higher than that of *E. coli* DQ0 (Fig. 6d, f, Supplementary Fig. 39B). After that, the switch time in the dark was optimized before starting fermentation, and the PLH content was increased to 47.5 wt% (Supplementary Fig. 39D). When this culture were scaled up to 5 L fermenter, the PLH titer, content, and DCW of *E. coli* DQ8 under the light condition were 14.31 g L^−1^, 53.8 wt%, and 26.6 g L^−1^, which were 137.71, 100.97, and 18.33% higher than that of *E. coli* DQ0, 34.87, 22.66, and 9.96% higher than that of *E. coli* DQ280, 114.22, 86.87, and 14.61% higher than that of *E. coli* DQ8 under the dark condition, respectively (Fig. 6e, Table 2).

**Table 2:** Differences in wild type and engineered *E. coli* on PLH production in 5 L fermenter.

Chemical	Strains characteristics	PLH contents (wt%)	Titer (g L^−1^)	Productivity (g L^−1^ h^−1^)	DCW (g L^−1^)
PLH	*E. coli* DQ0 (PLH pathway)	26.77	6.02	0.07	22.48
	*E. coli* DQ280 (PLH pathway + C and D period)	43.86 (*p* = 0.011)	10.61 (*p* = 0.012)	0.13 (*p* = 0.012)	24.19 (*p* = 0.204)
	*E. coli* DQ8 in the dark condition (PLH pathway + BARNA-controlled C and D period, dark)	28.79 (*p* = 0.220)	6.68 (*p* = 0.259)	0.08 (*p* = 0.259)	23.21 (*p* = 0.781)
	*E. coli* DQ8 in the light condition (PLH pathway + BARNA-controlled C and D period, light)	53.8 (*p* = 0.003)	14.31 (*p* = 0.001)	0.17 (*p* = 0.001)	26.6 (*p* = 0.007)

Significance (*p*-value) was relative to the control strain *E. coli* DQ0; Differences were determined by two-tailed Student’s *t*-test.

## Discussion

In this study, the BANA and BARNA light-powered systems were constructed and employed to spatiotemporally regulate the cell division of *E. coli*. When the BANA light-powered system was used to shorten cell division of *E. coli* DN4, the SSA and acetoin titer of *E. coli* DN4 were increased to 3.7 μm^−1^ and 67.2 g L^−1^, which were 20.39 and 39.19% higher than these of *E. coli* D1, respectively. When the BARNA light-powered system was used to prolong the cell division of *E. coli* DQ8, the MCV, PLH content, DCW, and PLH titer of *E. coli* DQ8 were increased to 52.6 μm^3^, 53.8 wt%, 26.6 and 14.31 g L^−1^, which were 13.65-fold, 2.01-fold, 1.18-fold, and 2.38-fold higher than these of *E. coli* DQ0, respectively. These results demonstrated that engineering the C and D periods of cell division represented an useful strategy to streamline the efficiency of microbial cell factories.

Cell division in *Escherichia coli* is mediated by a large protein complex and deoxyribonucleotides^31,37^, which are regulated by two factors: (i) a positive regulator: the *nrdAB*, *nrdA*, *nrdB*, and *nrdD* genes in the C period of cell division catalyze deoxyribonucleoside diphosphate for dNTP production to promote DNA replication for cell division, and then the *ftsZA*, *ftsZ, ftsA*, *ftsN*, and *ftsQ* genes in the D period of cell division assemble into a Z-ring scaffold to accelerate cell division; (ii) a negative regulator: the deletion of the *nrdAB*, *nrdA*, *nrdB*, and *nrdD* genes in the C period of cell division can perturb dNTP synthesis, and then the *sulA, minC, minD, minE*, and *ftsH* genes in the D period of cell division are the inhibitors of cell division. To regulate cell division at multiple levels, optogenetics is a promising strategy in a noninvasive, reversible, and spatiotemporal way^33^. For example, a blue light feedback gene circuit was used to reversibly regulate pyruvate decarboxylation to decouple cell growth and chemical production, resulting in isobutanol and 2-methyl-1-butanol production up to 8.49 g L^−1^ and 2.38 g L^−1^, respectively^33^. In addition, red light and blue light were utilized to regulate the Bphs and BlrP1 proteins for biofilm formation^44^. Based on these studies, an optogenetics-based cell division strategy was proposed: the optogenetic strategy was used to shorten or prolong the C+D period of cell division by spatiotemporally controlling the genes involved in the C+D periods of cell division in different phases with various light illumination intensities and times. Thus, two different light-powered systems, BANA and BARNA, were constructed. A BARNA light-powered system was used for weak expression of *nrdA* and overexpression of *nrdAB, ftsZA*, and *sulA* in *E. coli* DQ8, and thus the C and D periods of cell division were prolonged. As a result, this system causes the mini-cell phenotype^29^ and increases the cell count and cell growth^30,45^. To hinder DNA replication and decrease cell division frequency, a BARNA light-powered system was used for weak expression of *nrdA* and overexpression of *nrdAB, ftsZA*, and *sulA* in *E. coli* DQ8, and thus the C and D periods of cell division was prolonged. Thus, this system causes a large-cell phenotype and decreases cell count and cell growth^13,46^.

As a vital physiological parameter of industrial microbes^20,47^, SSA could increase mass transfer by enhancing the nutrient uptake rate and cell growth and improve cell density by changing the rheology of cultures. During industrial fermentation, SSA can be affected by mechanical pressure, medium contents, and chemical stress^16^. Thus, many efforts have showed their application potential in regulating SSA, such as microparticle cultivation^20^, fermentation optimization^48^, and laboratory adaptive evolution engineering^49^. Based on these strategies, the efficiency of chemical production will be enhanced by increasing the SSA of industrial microbes. In this study, when the C and D periods of cell division were shortened by BANA, the SSA was showed a 20.39% increase. As a result, the acetoin titer was increased by 39.19% compared to that of *E. coli* D1. These results indicated that acetoin production was improved by accurately controlling cell division and engineering SSA. This study was different from the previous studies that engineered target metabolic pathways^41^ and eliminated carbon catabolite repression^50^.

The MCV of industrial microbes is an important physiological parameter^51^, where we want to maximize inclusion body production. In addition, MCV should be also considered to improve the efficiency of upstream and downstream bioprocessing through faster precipitation of cells with relatively higher gravity. To manipulate the MCV of industrial microbes, a series of strategies have been developed such as perturbing the peptidoglycan cell wall synthesis to obtain a more elastic and flexible cell structure^24^, screening the cytoskeletal mutants for morphological properties^25^, and disturbing the phosphatidylinositol biosynthesis in vivo for the formation of filamentous structures^23^. In this study, when the C and D periods of cell division were prolonged by BARNA light-powered systems, the MCV of *E. coli* DQ8 was showed a 13.65-fold increase. As a result, the cell space for PLH accumulation was increased, and thus the PLH content of *E. coli* DQ8 was increased by 2.01-fold compared to that of *E. coli* DQ0. These results indicated the PLH production was improved by accurately regulating the C and D periods of cell division to increase the MCV of *E. coli* DQ8. Weak expression of *nrdA* could prolong the C period of cell division by disturbing intracellular dNTP synthesis and DNA replication, and overexpression of *sulA* could prolong the D period of cell division by hindering Z ring formation and divisome assembly^46^. Thus, the frequency of cell division would be affected by prolonging cell division^29^, leading to an increase in cell size. The larger cell space for PLH accumulation improved the PLH content, titer, and DCW. The strategy in this study was different from the previous strategies, such as engineering the target metabolic pathways^52^, utilizing the post translational metabolic switching^53^, and controlling the cofactor ratio^54^.

In this study, an optogenetics-based cell division strategy was developed, which is noninvasive and incorporates spatial, temporal and reversible control. The SSA and MCV of *E. coli* were increased by shorting or prolonging the C and D periods of cell division using a BANA or BRANA light-powered system, respectively. Based on this, the higher SSA or MCV values led to the higher efficiency of acetoin or PLH production. Our results demonstrated that increasing the SSA and MCV by manipulating cell division could enhance the targeted chemical production. Furthermore, this optogenetic-based cell division strategy may provide an approach to construct microbial cell factories for high-value chemical production.

## Methods

### Strains and culture conditions

All plasmids and bacterial strains used in this study were listed in Supplementary Data 1 and 2. Luria-Bertani (LB) broth and plates were used for strain selection and propagation. Kanamycin (50 mg L^−1^), ampicillin (100 mg L^−1^), spectinomycin (30 mg L^−1^), anhydrous tetracycline (200 ng mL^−1^), IPTG (100–500 μM) was added appropriately according to different conditions. The key gene targets of cell division were screened using IPTG. For constructing *E. coli* DQ280, the *nrdAB, ftsZA*, and *sulA* were constitutively expressed by promoter *P*_J23119_, and *nrdA* was expressed by weak promoter *P*_J23113_ with weak RBS B0033. The expression of *nrdAB, nrdA, nrdB*, and *nrdD* in the corresponding gene deletion mutants were controlled by low concentration of IPTG (10 μM) and low copy plasmid. The effect of IPTG concentration on gene expression was added in Supplementary Note 5.

For acetoin production, seed cultures were used for fermentation by transferring fresh colonies to a 30 mL tube containing 5 mL LB medium. After culturing for 10 h at 37 °C and 200 rpm, this fermentation solution was inoculated into a 250 mL flask with 50 mL fermentation medium^55^ (100 g L^−1^ glucose, 15 g L^−1^ tryptone, 15 g L^−1^ yeast extract, 3 g L^−1^ KH_2_PO_4_, and 3 g L^−1^ K_2_HPO_4_, and 0.4 g L^−1^ MgSO_4_) with an initial optical density at 600 nm (OD_600_) of 0.1, and then 10 g L^−1^ CaCO_3_ was added as an acid neutralizing agent. For fed-batch cultures in a 5 L fermenter (Shanghai Baoxing Biological Engineering Equipment Co. LTD), seed cultures were used for fermentation by transferring fresh colonies to a 250 mL flask containing 50 mL LB medium. Acetoin fermentation was carried out in a 5 L fermenter containing 3 L fermentation medium with 5% inoculum size and 90 g L^−1^ initial glucose, and then supplied with 800 g L^−1^ glucose at 48 h. Acetoin fermentation was maintained at pH 6.0, rotate rate 400 rpm, air flow 0.5 vvm, and 35 °C by the automatic addition of 4 M NaOH or 2 M HCl.

For PLH production, seed cultures were used for fermentation by transferring fresh colonies to a 30 mL tube containing 5 mL LB medium. After culturing for 10 h at 37 °C and 200 rpm, this fermentation solution was inoculated into a 250 mL flask with 50 mL MR medium^56^ (60 g L^−1^ glucose, 6.75 g L^−1^ KH_2_PO_4_, 2 g L^−1^ (NH_4_)_2_HPO_4_, 0.85 g L^−1^ citric acid, 3 g L^−1^ yeast extract, 10 mL trace metal solution per liter; pH 6.8), with an initial OD_600_ of 0.1, and then 10 g L^−1^ CaCO_3_ was added as an acid neutralizing agent. The composition of the trace metal solution was 10 g L^−1^ FeSO_4_·7H_2_O, 2.2 g L^−1^ ZnSO_4_·7H_2_O, 0.58 g L^−1^ MnSO_4_·4H_2_O, 1 g L^−1^ CuSO_4_·5H_2_O, 0.1 g L^−1^ (NH_4_)_6_Mo_7_O_24_·4H_2_O, 0.2 g L^−1^ Na_2_B_4_O_7_·10H_2_O and 10 mL of 35% HCl per liter. For fed-batch cultures in a 5 L fermenter (Shanghai Baoxing Biological Engineering Equipment Co. LTD), seed cultures were used for fermentation by transferring fresh colonies to a 250 mL flask containing 50 mL LB medium. PLH fermentation was carried out in a 5 L fermenter containing 3 L fermentation medium with 5% inoculum size and 60 g L^−1^ initial glucose, and then supplied with 800 g L^−1^ glucose at 36 h. PLH fermentation was maintained at pH 6.5, rotate rate 500 rpm, air flow 1 vvm and 37 °C by the automatic addition of 4 M NaOH or 2 M HCl. Cell density was measured at 600 nm by a spectrophotometer.

### Optogenetics working conditions

For optogenetics LED illustration, cells were illuminated using a custom built 300 mm × 300 mm × 50 mm LED blue light and NIR light panels with adjustable ON/OFF pulsing and intensity. Two 450 nm blue light or two 650 nm NIR light (The characteristic of LED panel is 22 W, 24 V, 1.7 A; MODEL: HF-FX160, square light source) were opened to illuminate fermenter for activating gene expression. Light panel was placed 5 cm away from the vessel walls. These equipments were purchased from KOMA Vision Technology Company and LEMONS Co., Ltd, CHINA. Switching power supply AC/DC ADAPTER (MODEL: SPF-1210) was used to power the lighting apparatus simultaneously (AC 100–240 V ~50/60 Hz; OUTPUT: DC 12 V, 10 A). For improving the applicability and stability of scale, fermentation process was adopted a wrap-round illumination-type by two blue light and two NIR light illumination sources to surround 5 L and 10 L fermenters, respectively.

For optogenetics-controlled mKate and BFP expression in 5 L fermenter and 10 L fermenters (Labfors 5 Bacteria, INFORS, Switzerland), the culture pH, air flow, temperature were kept at 6.0, 1 vvm, and 37 °C, respectively. Samples were taken for every 12 h to measure OD_600_ and DCW of cell cultures. The average measurements were measured with three independent fermentation and the error bars were corresponded to the standard deviations of those three measurements. To analyze the expression of fluorescence proteins, fermentation was carried out in a 10 L fermenter containing 7.5 L fermentation medium with 5% inoculum size, and then supplied with 100 g L^−1^ yeast extract, 25 g L^−1^ peptone, and 400 g L^−1^ glycerol at a rate of 16 mL h^−1^ for high density fermentation. When cells density was increased to an OD_600_ of 10 in 5 L and 10 L fermenters, blue light or NIR light sources was opened to activate the expression of fluorescence proteins.

### DNA manipulation and plasmid construction

Gene deletions were performed according to the Red homologous recombination method^40^. All plasmids were constructed using basic molecular cloning techniques and Gibson assembly. To construct the acetoin biosynthesis pathway, the *budA* and *budB* genes were amplified by PCR from genomic DNA of *S. marcescens* H30. Similarly, the *nox* gene from *L. breris* was synthesized by Suzhou Genewiz Biotechnology with codon optimization. For constructing the PLH biosynthesis pathway, the *pct* gene from *M. elsdenii*, *phaA* and *phaB* genes from *R. eutropha*, and *phaC* gene from *Pseudomonas sp*. were synthesized by Suzhou Genewiz Biotechnology with codon optimization. The *el222*, *bphS, bphO, yhjH, mrkH* genes were synthesized by Suzhou Genewiz Biotechnology with codon optimization. Supplementary Data 2 and 3, gives a list of the relevant parts, sequences, and sources. Primers used in this study were listed in Supplementary Table 1.

### BANA and BARNA implementation

BANA and BARNA system were constructed. In short, the BANA and BARNA were implemented with a two-plasmid system, respectively. Supplementary Data 1–3 showed the names and sequences of BANA and BARNA. In BANA, two plasmids, P_Trc_-E-P_J23119_-SO-P_J23119_-Y_30_M, and pBLind-v1-P_mrkA_-BFP, were used as input plasmids with various blue-light and NIR-light illumination. In BARNA, two plasmids, P_Trc_-E-P_J23119_-SO-P_J23119_-Y_30_M and pBLirnd-v1-P_mrkA_-BFP, were used as input plasmids with various blue-light and NIR-light illumination.

To test BANA, a colony of BANA cells containing a controller plasmid P_Trc_-E-P_J23119_-SO-P_J23119_-Y_30_M and an actuator plasmid pBLind-v1-P_mrkA_-BFP was inoculated into medium with the corresponding ampicillin and spectinomycin for overnight (12–14 h) at 37 °C. Then, seed cultures were inoculated in the refresh LB for 12–14 h under the different blue-light (450 nm) or NIR-light (650 nm) illumination. This experiment was repeated with a different starting colony for three biological replicate. All cultures were grown in 50 mL medium in 250 mL shake flasks at 200 rpm. Finally, fluorescence density was analyzed (see below). To test BARNA, its method was similar to that of BANA.

### Analytical methods

The OD_600_ was measured using a spectrophotometer. Glucose analysis was quantified by the biosensor SBA-90E biological sensor. Acetoin were determined by high-performance liquid chromatography using an Aminex HPX-87H column (7.8 × 300 mm; Bio-Rad Laboratories, Inc., Hercules, CA, USA) at 60 °C with 0.05 mM sulfuric acid as the mobile phase. The injection volume was 20 μL, and the flow rate was 0.6 mL min^−1^. Bacterial cells were harvested by centrifugation at 8000 r min^−1^ for 10 min. The supernatant was discarded, and then cells were washed twice with 20 mL distilled water. Dry cell weight (DCW) was assayed after vacuum lyophilization. PLH contents were quantitatively analyzed by gas chromatography (GC-2014, SHIMADZU, Japan) after methanolysis of lyophilized cells in chloroform. Analytical P3HB and P(GA-LA) purchased from Sigma-Aldrich were used as analysis standards.

### The parameters of *E. coli* morphology

The model of *E. coli* was showed in Supplementary Fig. 40, which was used to calculate the mean cell width and MCL. The mean cell width was calculated from cell midline to cell midline. The MCL was calculated from pole to pole. The parameters^25^ of *E. coli* morphology were used to segment cells and to identify cell outlines from Nikon eclipse 80i microscope (Nikon corporation) image for 100 individuals cell. Specific surface area was calculated by dividing *S* (1) by *V* (2).

1 $$S = {\mathrm{2}}\pi R(L - {\mathrm{2}}R) + {\mathrm{4}}\pi R^{\mathrm{2}},$$

2 $$V = \pi R^{\mathrm{2}}(L - {\mathrm{2}}R) + {\mathrm{4}}/{\mathrm{3}}\pi R^{\mathrm{3}},$$

where *R* is the mean cell width and *L* is the MCL, *V* is cell volume, and *S* is surface area.

### Assay of field emission scanning electron microscope (FESEM) and transmission electron microscopy (TEM)

Bacterial cells were collected by centrifugation at 1500 r min^−1^ for 2 min, and then washed twice with phosphate buffer at pH 7.2. The supernatant was discarded, and then 200 μL 2.5% glutaraldehyde (pH = 7.2) was added for sample fixation at room temperature for 2–3 h. Subsequently, samples were re-washed twice as described above. Finally, samples were prepared by the FESEM or TEM (FEI Company, Quanta-200 and H-7650).

### Assay of the C and D periods and qPCR measurements

Measurement of the D period was based on measuring the ori/ter ratio by qPCR (Bio-Rad, Hercules, CA) as described in Liu et al.^36^ Two milliliter bacteria culture (OD_600_~0.4) was collected and immediately frozen in liquid nitrogen before sending to freezer for storage. After 24 h, the bacteria genome was extracted using a bacteria total genome DNA extraction kit. The DNA concentration was quantified by measuring the absorbance at 260 nm UV light with the NanoDrop ND-1000 UV–Vis Spectrophotometer (Thermo Scientific). Primers were used for amplifying the DNA region proximal to the origin (oriC) and terminus (ter)^57^. The qPCR reactions were performed with a SuperReal Premix SYBR Green Plus kit according to its manual. For each PCR reaction, 20 μL sample contained 10 ng of DNA, 0.6 μM of each primer and 10 μL of 2× SYBR Green Supermix. The reaction process was carried out in an Opticon 2 Real-time PCR system (Bio-Rad, Hercules, CA, USA) according to the following protocol: 95 °C for 3 min, followed by 40 cycles of 95 °C for 30 s, 60 °C for 30 s, and 72 °C for 30 s. The qPCR product was checked in a 2% agarose gel to ensure the specificity of PCR amplification. The 16S rRNA was used as the reference gene to normalize the expression level. For each RNA preparation, at least three independent real-time PCR measurements were performed. RT-PCR primers were listed in Supplementary Table 2.

Calculation of the C period is based on Zhu et al.^46^ (Eqs. 3, 4). Briefly,

3 $${\mathrm{ori}}/{\mathrm{ter}} = 2^{({\mathrm{C}}/\tau )}$$

4 $$\tau = {\mathrm{ln2}}/\mu,$$

*τ* is the mass doubling time; *μ* is the specific growth rate.

The C period was measured by qPCR, a DNA increment method was applied. Chloramphenicol was used to inhibit the initiation of new rounds of replication, cells were collected by filtration, and the DNA amount was measured by the diphenylamine colorimetric method. The C period was calculated by measuring DNA amount after inhibiting the replication initiation. After DNA initiation blockage corresponded to origins (Ori)/genome equivalents for the cell populations, the relative DNA amount was changed. Genome equivalents/cell was calculated using DNA/cell to divide the molecular mass of *E. coli* chromosome. DNA/cell was calculated by measuring total DNA/OD and cell count/OD^46^. Therefore, origins/cell was calculated by genome equivalents/cell times and the ori/genome equivalents ratio. The D period was further derived based on Eq. 5.

5 $${\mathrm{ori}}/{\mathrm{cell}} = 2^{({\mathrm{C}} + {\mathrm{D}})/{\uptau}},$$

### Nile red staining

Cells (1 mL MR culture) were washed once with PBS, and then re-suspended in 1 mL PBS. Five microliter of Nile Red (0.1 mg mL^−1^ in acetone) was added to cell suspension, and then incubated at room temperature, after culturing 5 min with dark condition. Cells were washed twice by 100% ethanol and 75% ethyl alcohol, respectively. Cells were imaged with a Nikon eclipse 80i microscope (Nikon Corporation).

### PI staining

The number of cells (about 10^6^ individual·mL^−1^) was collected for PI staining. Then, it were centrifuged at 500–1000 r·min^−1^ for 5 min to discard culture medium. Next, cells were washed with 3 mL PBS twice, and centrifuged. After centrifugation, PBS was discarded. Cells were dyed by 1 mL PI dying at 4 °C for 5–30 min under the dark condition. Cells were imaged with a Nikon eclipse 80i microscope (Nikon Corporation).

### X-gal assay

The colorless compound X-gal (dissolved in *N,N*-dimethylformamide) could be converted into galactose by β-galactosidase, forming the blue substance 5-bromo-4-indigo. In this study, the engineered *E. coli* was transformed into the plates containing 40 μg mL^−1^ X-gal, and then cultured at 37 °C for 32 h in the dark or irradiated with blue-light (450 nm, 0.8 W/cm^2^). Based on this, the colony that produced β-galactosidase was turned into blue.

### Creatine reaction assay

For creatine reaction assay, 10 mL reaction system included 10% NaOH, 5% beta-naphthol (dissolves in n-propanol), 0.5% creatine, and deionized water. The proportion of the reaction assay was 1:1:1:7. Hundred microliter of fermentation broth was added into reaction system to start reaction in test tube. This reaction was lasted for 30 min at 30 °C. The red reaction liquid was positive with acetoin titer, and could be used as a qualitative detection method for instructing acetoin titer.

### Flow cytometry assays

For flow cytometry analysis, *E. coli* cells were washed twice with PBS, and then resuspended to an OD_600_ of 0.2. The assays were performed by a LSR Fortessa instrument (BD Biosciences) using DAPI (BFP) and PE-TxRed (mKate) channels. The voltage gains for each detector were set to DAPI, 407 V and PE-TxRed, 650 V. Compensation was performed using cells that only expressed BFP or mKate. For each sample, at least 20,000 counts were recorded using a 0.5 mL s^−1^ flow rate. A gate was previously designed based on forward and side scatter (>99% cells were chosen for the analysis of fluorescence density percentage). All data were exported in FCS3 format and processed using FlowJo software (FlowJo-V10).

### The bioimage of agarose plates

*E. coli* strains harboring BLAT, BLRT, NRAT, BANA, and BARNA systems were cultured overnight, respectively^38^. A photomask was placed on the bottom-side of the prepared agar plate and used aluminum oxide to avoid light illumination. Seed cultures were transferred to fresh medium for OD_600_ = 0.8, and then 400 μL bacteria cultures was used to plate for bioimaging. The whole setup was kept inside the incubator at 37 °C. The LB plates were illuminated under 450 nm blue light or 650 nm NIR light or without illumination for 36 h. Fluorescence images were collected by IBright FL1000 (Thermo Fisher) and ChemiScope 6000 (Shanghai Qinxiang Scientific Instrument co. LTD).

### Cell viability assays

The viability of cells in LB plates was assessed by inoculating cells in fermentation medium. Cells were diluted to OD_600_ = 0.5 and 10 μL of 10× serially diluted cell suspension was spread on each agar plate with different strains. Then, the LB plates were cultured with 37 °C for 12 h. Next, the numbers of living cell in different culture time and different strains were calculated.

### Enzymatic assays

β-galactosidase^39^ gene was synthesized by GENEWIZ Biotechnology Co. LTD with codon optimization. ONPG (an analog of lactose) was used as substrates for assaying β-galactosidase activity. β-galactosidase could convert the colorless substrate oNPG into galactose and yellow-colored o-nitrophenol. o-Nitrophenol can be detected at 420 nm using a SpectraMax M3 plate reader. The diluted solution of β-galactosidase (0.1 mL) was added to 1.8 mL potassium phosphate buffer (50 mM, pH 6.5) containing 20 mM oNPG to start reaction. After incubation at 50 °C for 10 min, this reaction was stopped by adding 1 mL 1 M Na_2_CO_3_. All the extracellular activity represented the extracellular activity per ml medium supernatant. The method of β-glucuronidase assay was similar to β-galactosidase. 4-Nitrophenyl-β-d-glucuronide was converted to p-nitrophenol and β-gluconaldehyde glycoside by β-glucuronidase. For the enzymatic assay controlled by blue light or NIR light, culture temperature and rotation speed were set to 37 °C and 200 rpm, respectively. Culture medium was refreshed every 12 h to clean the residual β-galactosidase or β-glucuronidase for next assays.

### Assay of fluorescence intensity

The engineered *E*. *coli* strains used for assaying fluorescence intensity were plated on the LB plates for overnight at 37 °C, 200 rpm. After that, it was inoculated into 50 mL fresh LB with 2% inoculum size (vol/vol), and then cultured at 37 °C, 200 rpm. For assaying fluorescence intensity, the fluorescence of cell culture was detected by a SpectraMax M3 plate reader (Molecular Devices). The excitation and emission wavelengths of BFP were set at 402 ± 10 nm and 457 ± 10 nm, respectively. The excitation and emission wavelengths of GFP were set at 480 ± 10 nm and 515 ± 10 nm, respectively. The excitation and emission wavelengths of mKate were set at 588 ± 10 nm and 645 ± 10 nm, respectively. The excitation and emission wavelengths of DAPI were set at 358 ± 10 nm and 461 ± 10 nm, respectively.

### Statistical and reproducibility

All data were expressed as mean ± s.d. Differences between two groups were determined by two-tailed Student’s *t*-test and paired sample analysis through SPSS statistics software (SPSS V13.0). We have repeated each experiment at least three times of biological independent experiments.

### Reporting summary

Further information on research design is available in the Nature Research Reporting Summary linked to this article.

## Supplementary information

Supplementary Information

Reporting Summary

Description of Additional Supplementary Files

Supplementary Data 1

Supplementary Data 2

Supplementary Data 3

Supplementary Data 4

## Data Availability

Data supporting the findings of this work are available within the paper and its Supplementary Information files. A reporting summary for this Article is available as a Supplementary Information file. The datasets generated and analyzed during the current study are available from the corresponding author upon request. The source data underlying Figs. 1, 2, 3a, b, 4d–i, 5d–f, 6D–F, Tables 1, 2, as well as Supplementary Figures 1–12, 14, 15, 16b, c, 17, 18, 19B, 20–24, 26–31, 35–39 are provided in Source Data file. All SEM, TEM, fluorescence, bio-imaging pictures, and enzyme reaction of micrographs are also available at figshare (https://figshare.com/search?q=10.6084%2Fm9.figshare.9122534&searchMode=1).

## Source data

Source Data
