# Author Correction: Light-powered *Escherichia coli* cell division for chemical production

**DOI:** 10.1038/s41467-020-19642-8

**Published:** 2020-11-04

**Authors:** Qiang Ding, Danlei Ma, Gao-Qiang Liu, Yang Li, Liang Guo, Cong Gao, Guipeng Hu, Chao Ye, Jia Liu, Liming Liu, Xiulai Chen

**Affiliations:** 1 State Key Laboratory of Food Science and Technology, Jiangnan University, 214122 Wuxi, China; 2 Key Laboratory of Industrial Biotechnology, Ministry of Education, Jiangnan University, 214122 Wuxi, China; 3 Hunan Provincial Key Laboratory for Forestry Biotechnology, Central South University of Forestry and Technology, 410004 Changsha, China; 4 National Engineering Laboratory for Cereal Fermentation Technology, Jiangnan University, 214122 Wuxi, China

**Keywords:** Metabolic engineering, Metabolic engineering, Metabolic engineering

Correction to: *Nature Communications* 10.1038/s41467-020-16154-3, published online 8 May 2020.

The original version of this Article omitted a reference to previous work in reference 38, Jayaraman et al. Blue light-mediated transcriptional activation and repression of gene expression in bacteria. *Nucleic Acids Res.* **44**, 6994–7005 (2016), when describing construction of the blue-light activation tool (BLAT). The sentence “The construction of BLAT and BLRT follows a previously reported strategy^38^” has been added to the beginning of the second paragraph of the “Results” subsection “Constructing the basic tools for powering cell division”. Reference 38 has also been added to the sentence following: “To construct a BLAT, two units were designed and assembled (Fig. 4a): a blue optogenetics unit (BOU) to express light-sensitive protein EL222, and an activation reporter unit to replace the LuxR-binding region with an EL222-binding region by overlapping the -35 region (*E. coli* consensus -35 and -10 regions) of the LuxI promoter^38^.”

Furthermore, Fig. 4, panels a and b, are adapted from reference 38 Figure 1, panels a and c, and this was not clear in the figure legend. The sentence “Panels a and b are adapted from Jayaraman et al.^38^” has been added to the legend of Fig. 4 to clarify this.

These have been corrected in both the PDF and HTML versions of the Article.

