# Supplementary material for: Light-powered Escherichia coli cell division for chemical production
Source: Nat Commun. 2020 May 8;11:2262. doi: 10.1038/s41467-020-16154-3 (PMC7210317; doi:10.1038/s41467-020-16154-3)
Supplement: Supplementary file 1 — Supplementary Information [file 41467_2020_16154_MOESM1_ESM.pdf]

Light-powered *Escherichia coli* cell division for chemical production

Ding *et al.*

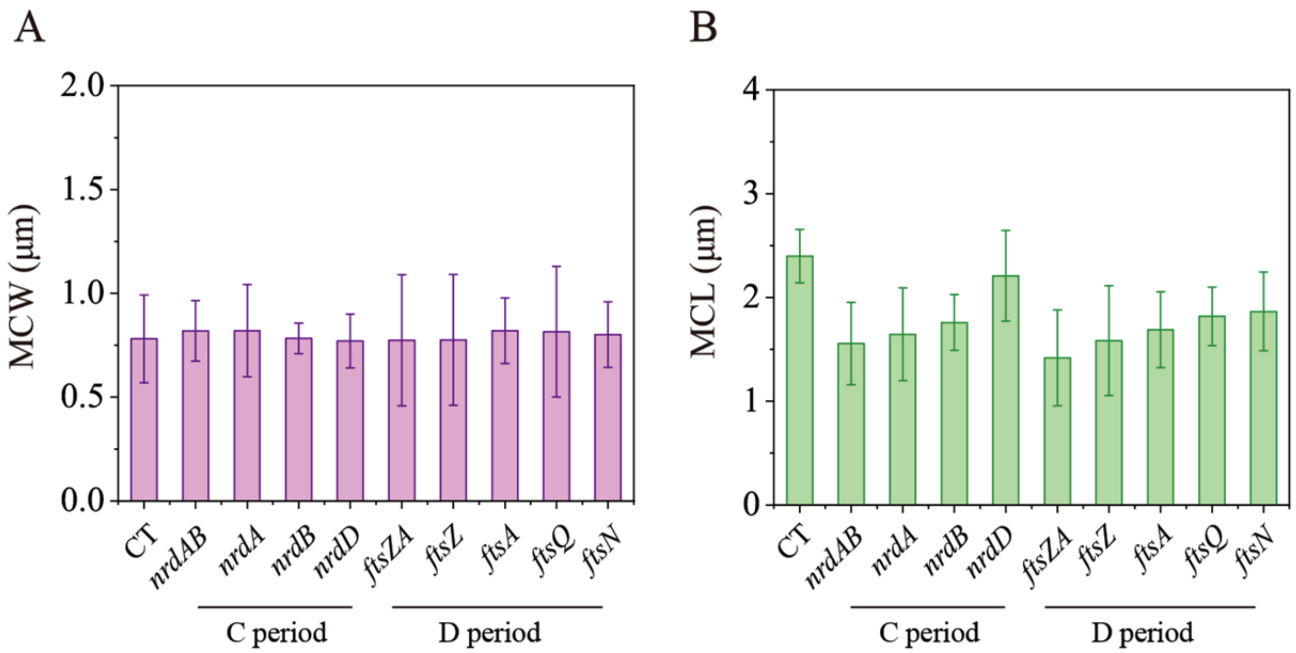

Supplementary Figure 1. Effect of shortening the C and D periods of cell division on the MCW and MCL. (A) Effect of shortening the C and D periods of cell division on the mean cell width (MCW), respectively. **(B)** Effect of shortening the C and D periods of cell division on the mean cell length (MCL), respectively. For A, B, values are shown as mean ± s.d. from three biological independent replicates, with total 100 cells (n=100). Source data are provided as a Source Data file.

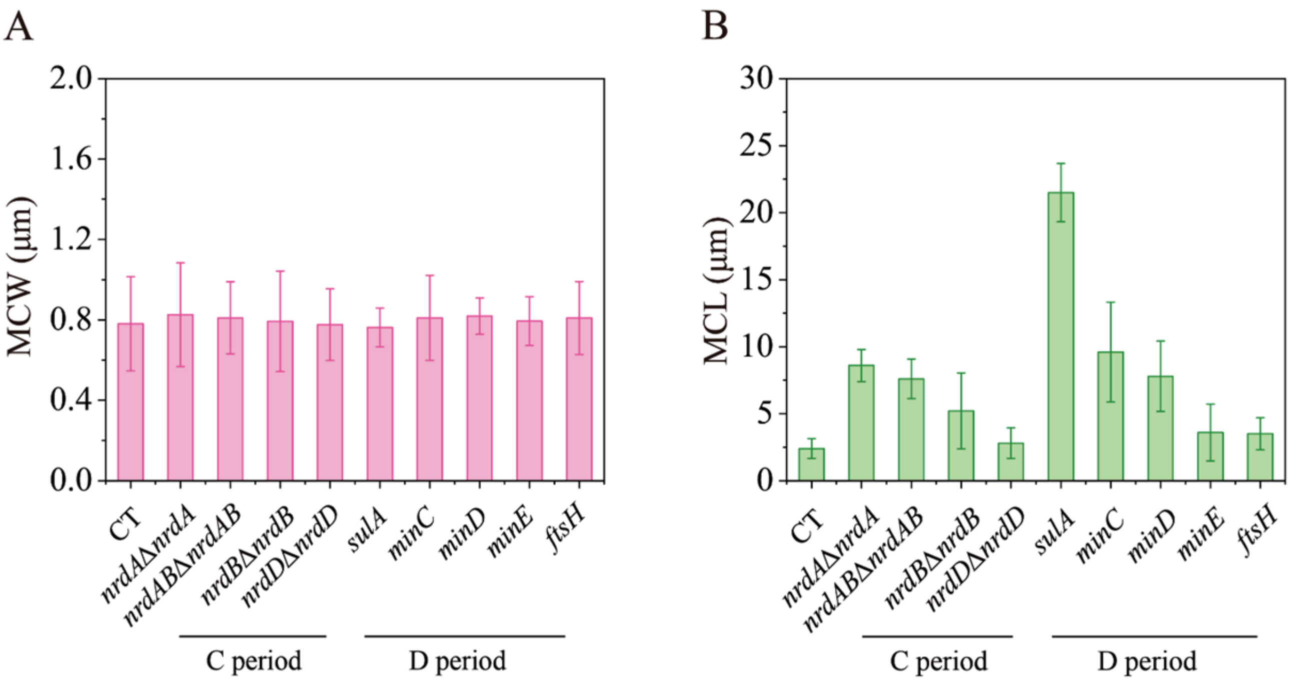

Supplementary Figure 2. Effect of prolonging the C and D periods of cell division on the MCW and MCL. (A) Effect of prolonging the C and D periods of cell division on the mean cell width (MCW), respectively. **(B)** Effect of prolonging the C and D periods of cell division on the mean cell length (MCL), respectively. For A, B, values are shown as mean ± s.d. from three biological independent replicates, with total 100 cells (n=100). Source data are provided as a Source Data file.

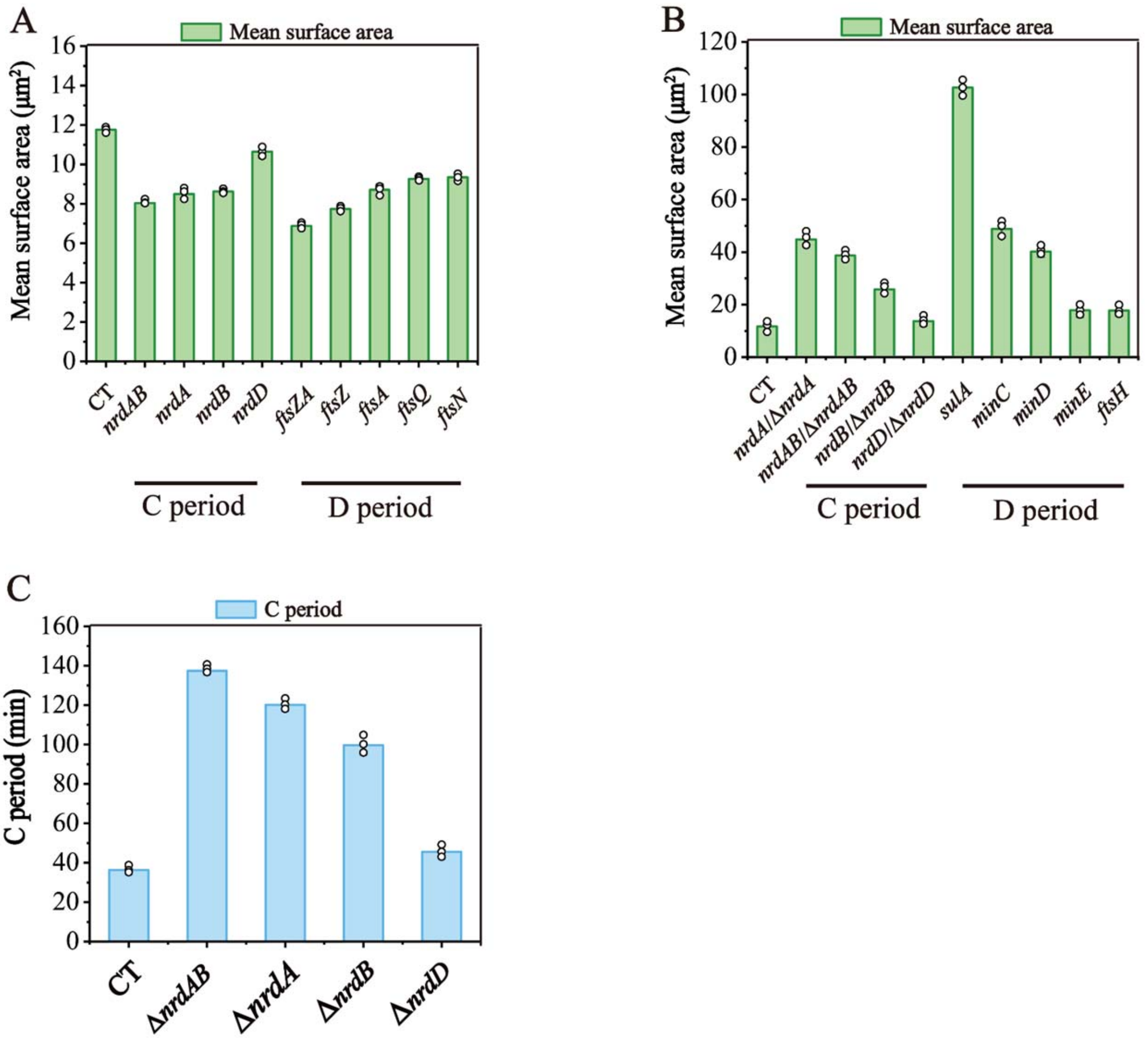

Supplementary Figure 3. Mean surface area, specific surface area, and C period with different strains. (A) The mean surface area of the shortened cell division. **(B)** The specific surface area of the prolonged cell division. **(C)** The C period of the Δ*nrdAB,* Δ*nrdA,* Δ*nrdB,* Δ*nrdD* mutant strains. For A-C, values are shown as mean ± s.d. from three (n = 3) biological independent replicates. Source data are provided as a Source Data file.

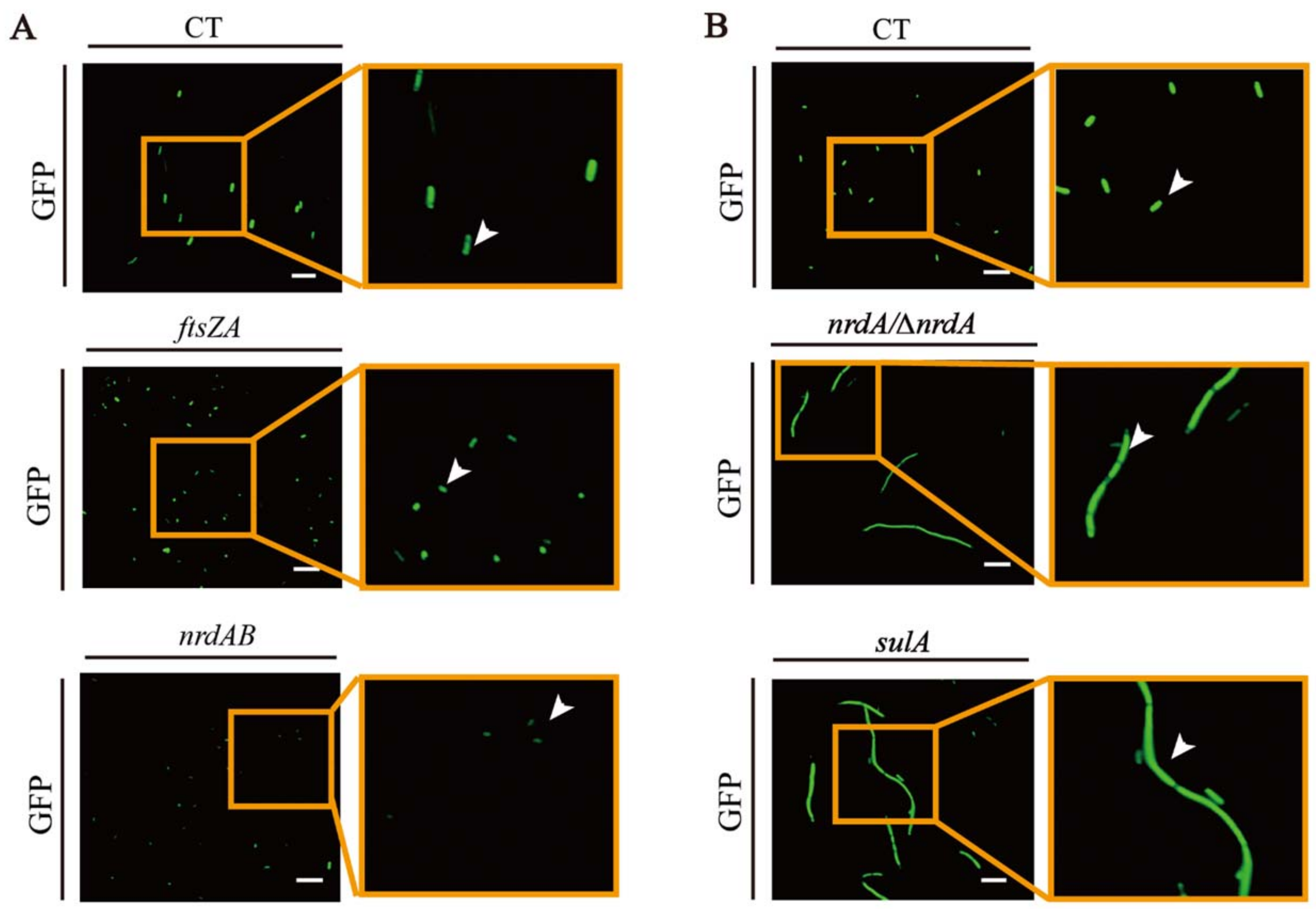

Supplementary Figure 4. Identifying cell morphology by regulating genes to shorten and prolong cell division. **(A)** Confirming the morphological variations of the engineered strains with *nrdAB* and *ftsZA* genes by *Nikon eclipse* 80i microscope. The shapes of *E. coli* showed the differences in white arrow. **(B)** Confirming the morphological variations of the engineered strains with *nrdA/ΔnrdA* and *sulA* genes by Nikon eclipse 80i microscope. For A, B, Scale bar is 10 µm. Source data are provided as a Source Data file.

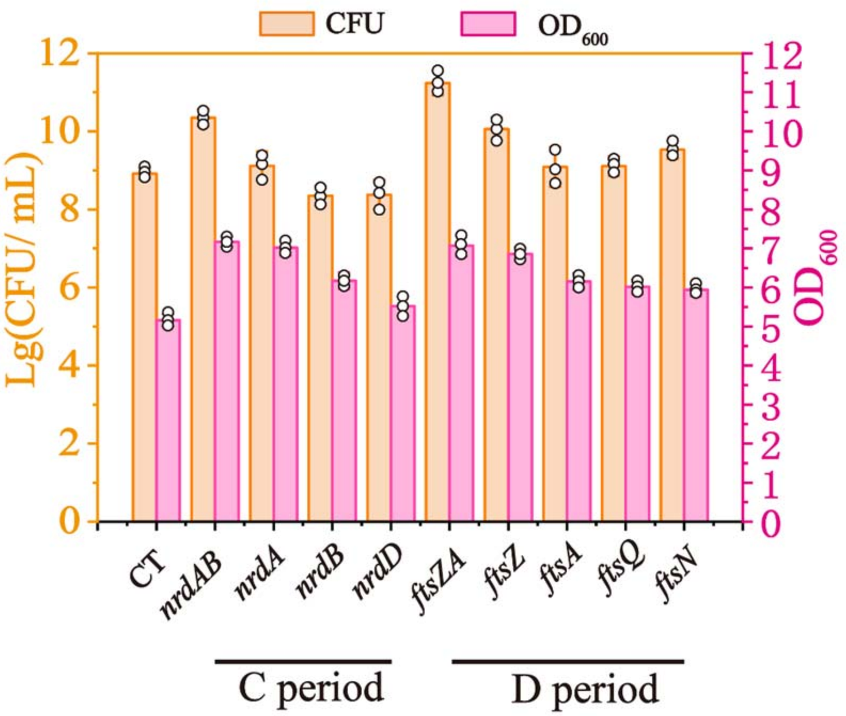

Supplementary Figure 5. Effect of shortening the C and D periods of cell division on cell count (c.f.u) and OD. Values are shown as mean ± s.d. from three (n = 3) biological independent replicates. Source data are provided as a Source Data file.

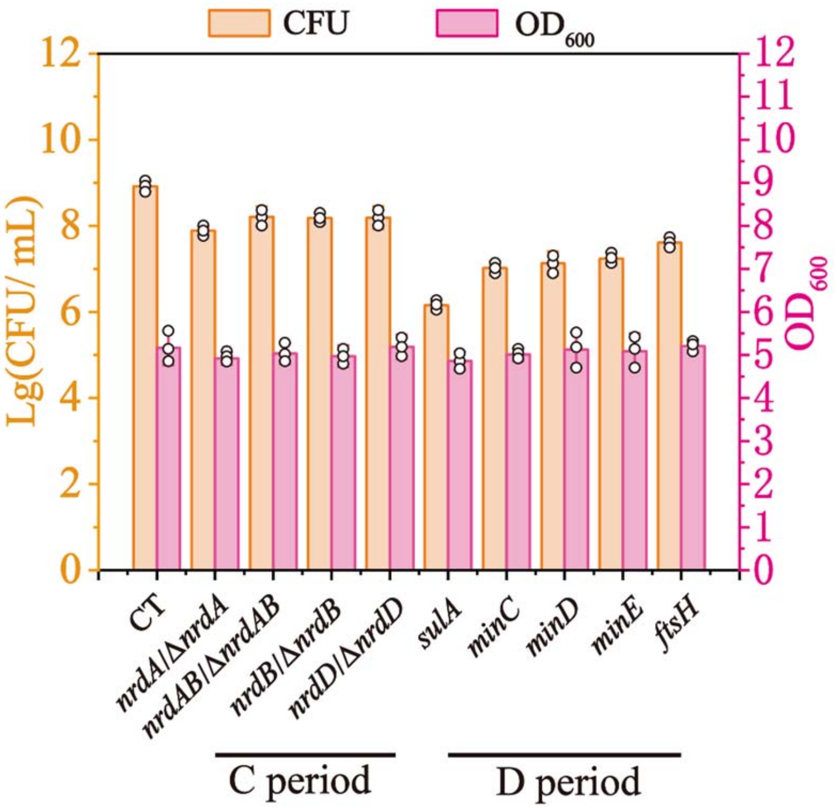

Supplementary Figure 6. Effect of prolonging the C and D periods of cell division on cell count (c.f.u) and OD. Values are shown as mean ± s.d. from three (n = 3) biological independent replicates. Source data are provided as a Source Data file.

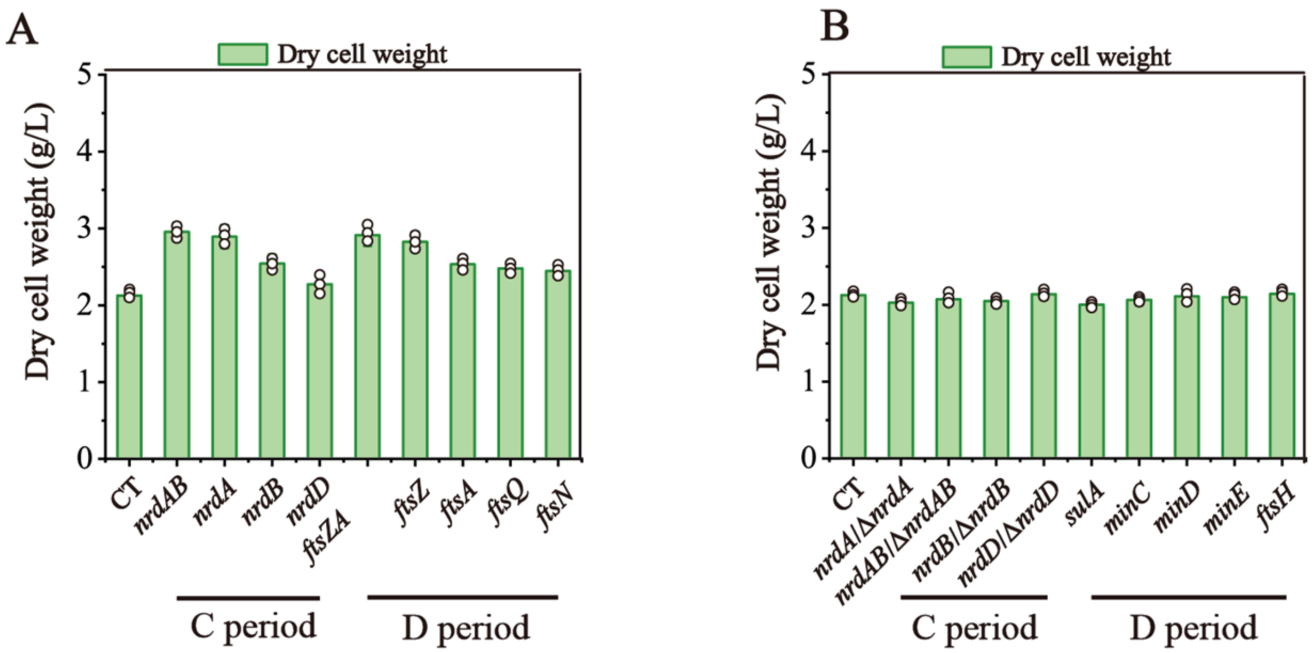

Supplementary Figure 7. Effect of morphology changes on the dry cell weigh. (A) Effect of shortening the C and D periods of cell division on the dry cell weight (DCW). **(B)** Effect of prolonging the C and D periods of cell division on the DCW. For A, B, values are shown as mean ± s.d. from three (n = 3) biological independent replicates. Source data are provided as a Source Data file.

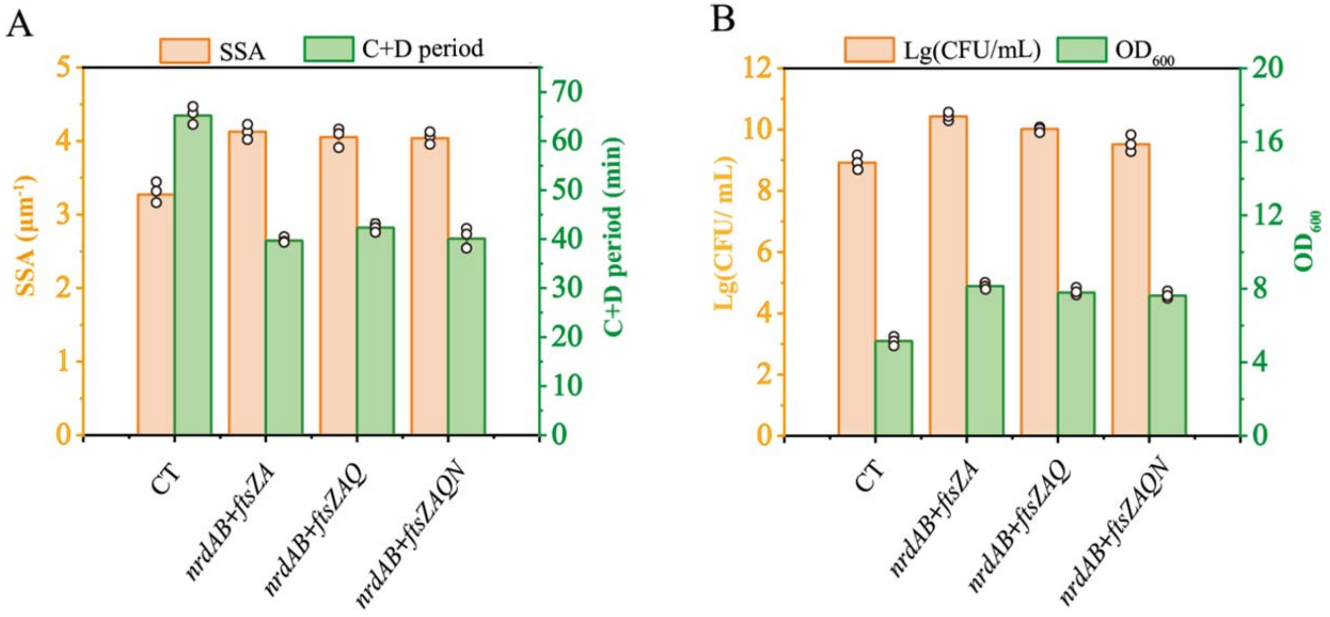

Supplementary Figure 8. Combining the *nrdAB* genes in the C period with the key genes in the D period. (A) Effect of the C and D periods of cell division on the specific surface area (SSA) and the C+D periods. **(B)** Effect of the C and D periods of cell division on cell count (c.f.u) and OD. For A, B, values are shown as mean ± s.d. from three (n = 3) biological independent replicates. Source data are provided as a Source Data file.

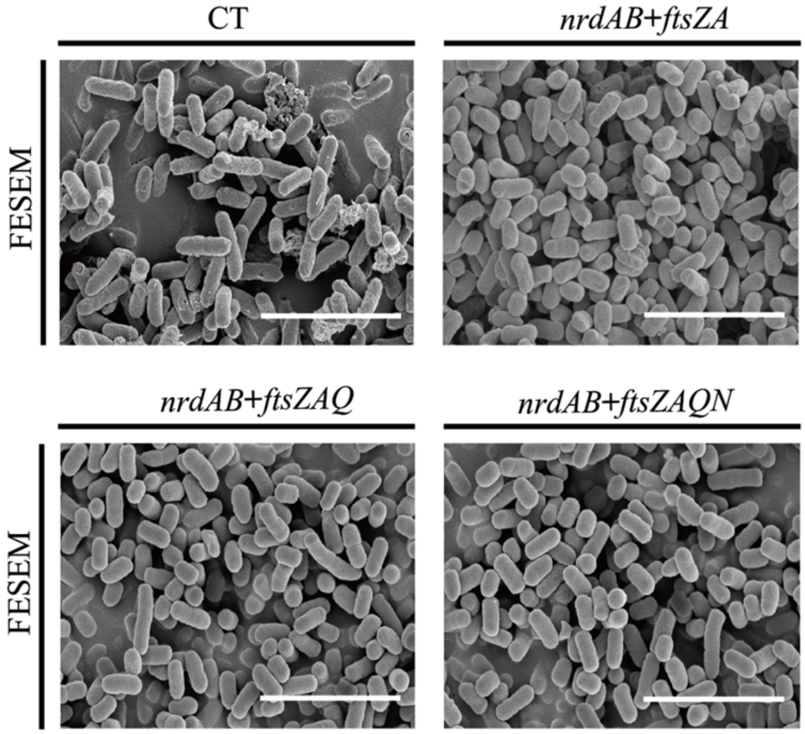

Supplementary Figure 9. Effect of gene combination on cell morphology by shortening the C and D periods of cell division. Scale bar is 5 μm. FESEM is the filed emission scanning electron microscopy. Source data are provided as a Source Data file.

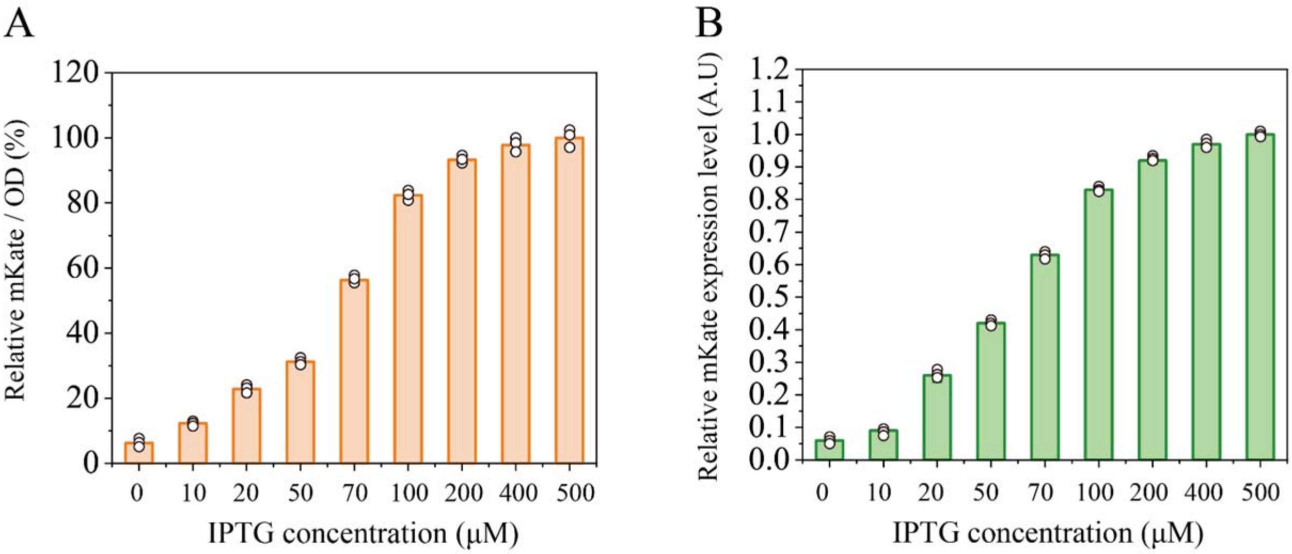

Supplementary Figure 10. Effect of IPTG concentrations on mKate expression. **(A)** Relative mKate gene expression with different IPTG concentrations. The data are normalized to the highest value obtained. **(B)** Relative mKate gene expression level with different IPTG concentrations. The data are normalized to the highest value obtained. The corresponding illustration was introduced in the Supplementary Note 5. For A, B, values are shown as mean ± s.d. from three (n = 3) biological independent replicates. Source data are provided as a Source Data file.

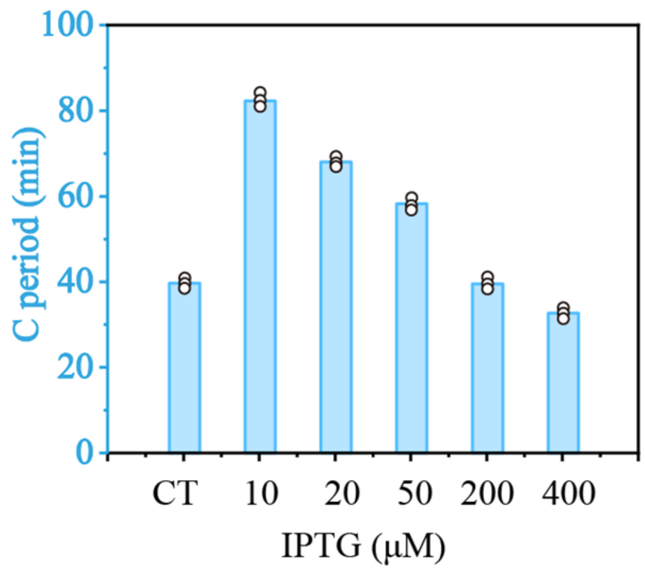

Supplementary Figure 11. Effect of IPTG concentrations on the C period of cell division in *nrdA/ΔnrdA* mutant. The corresponding illustration was introduced in the Supplementary Note 2. For A, values are shown as mean ± s.d. from three (n = 3) biological independent replicates. Source data are provided as a Source Data file.

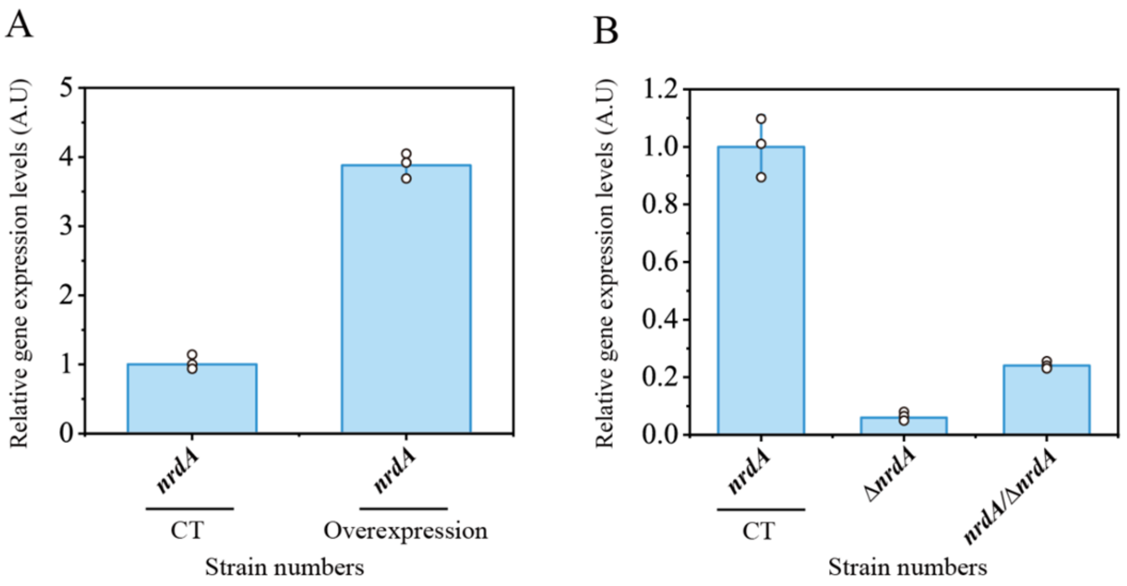

Supplementary Figure 12. The relative expression levels of *nrdA*, Δ*nrdA*, *nrdA*/Δ*nrdA* strains. (A) The relative expression levels of *nrdA* genes with or without 500 μM IPTG. **(B)** The relative expression levels of *nrdA*, Δ*nrdA*, *nrdA*/Δ*nrdA*. *nrdA*/Δ*nrdA* strains are weakly expressed by 10 μM IPTG. CT is the control group. The data are normalized to the corresponding gene expression in control group. The corresponding illustration was introduced in the Supplementary Note 2. For A, B, values are shown as mean ± s.d. from three (n = 3) biological independent replicates. Source data are provided as a Source Data file.

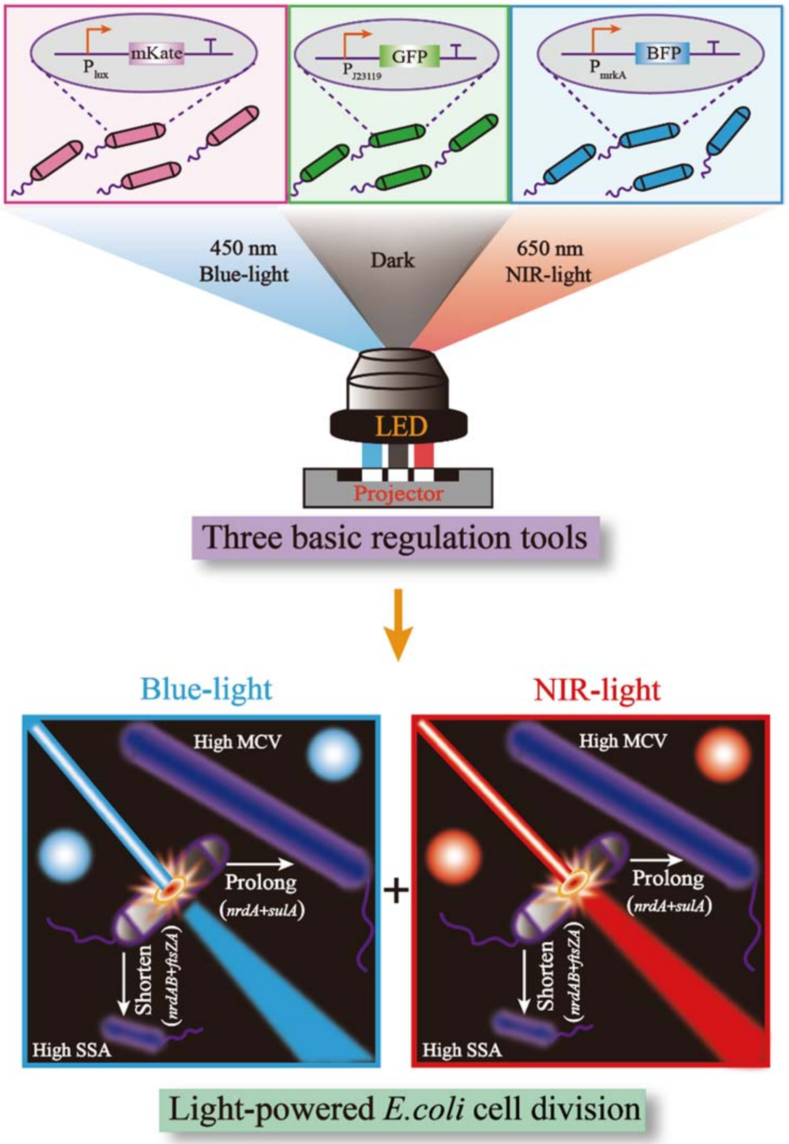

Supplementary Figure 13. Three basic optogenetics tools for regulating cell division.

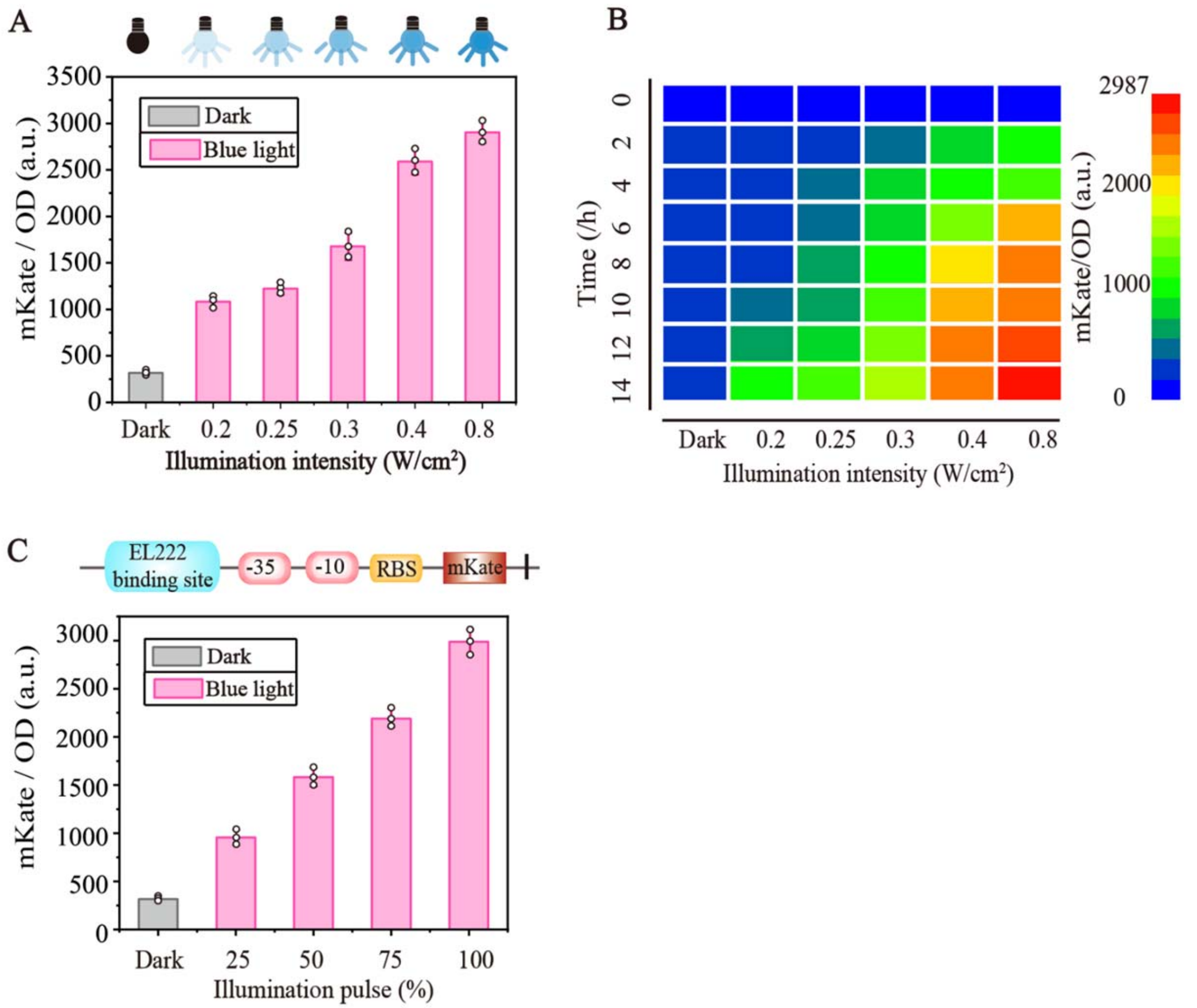

Supplementary Figure 14. The performance of the BLAT tool. (A) Dose-dependent activation with different illumination intensity (0-0.8 W/cm^2). **(B)** Dose-dependent activation with different illumination intensity and culture time (0-0.8 W/cm^2, 0-14 h). **(C)** Dose-dependent activation with different illumination pulse (0.8 W/cm^2, 0, 25%, 50%, 75% and 100%). For A-C, values are shown as mean ± s.d. from three (n = 3) biological independent replicates. Source data are provided as a Source Data file.

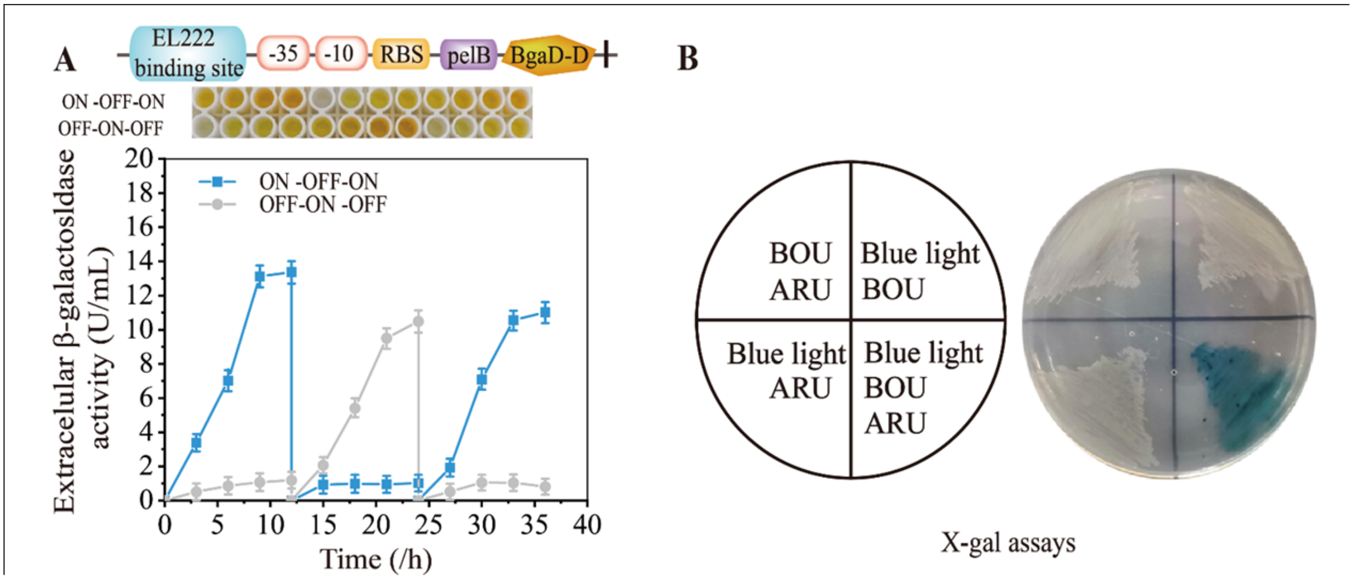

Supplementary Figure 15. The analysis of the BLAT tool. (A) Reversibility of the BLAT-triggered transgene expression with β-galactosidase by an ON-OFF-ON / OFF-ON-OFF switch. The secretroy signal peptide pelB is used to transport β-galactosidase to extracellular medium. The culture medium is refreshed every 12 h to clean the extracellular β-galactosidase. The inserted figure is the production of yellow NPG via β-galactosidase. **(B)** Converting X-gal into 5-bromo-4-indigo by β-galactosidase with BLAT regulation. For A, values are shown as mean ± s.d. from three (n = 3) biological independent replicates. Source data are provided as a Source Data file.

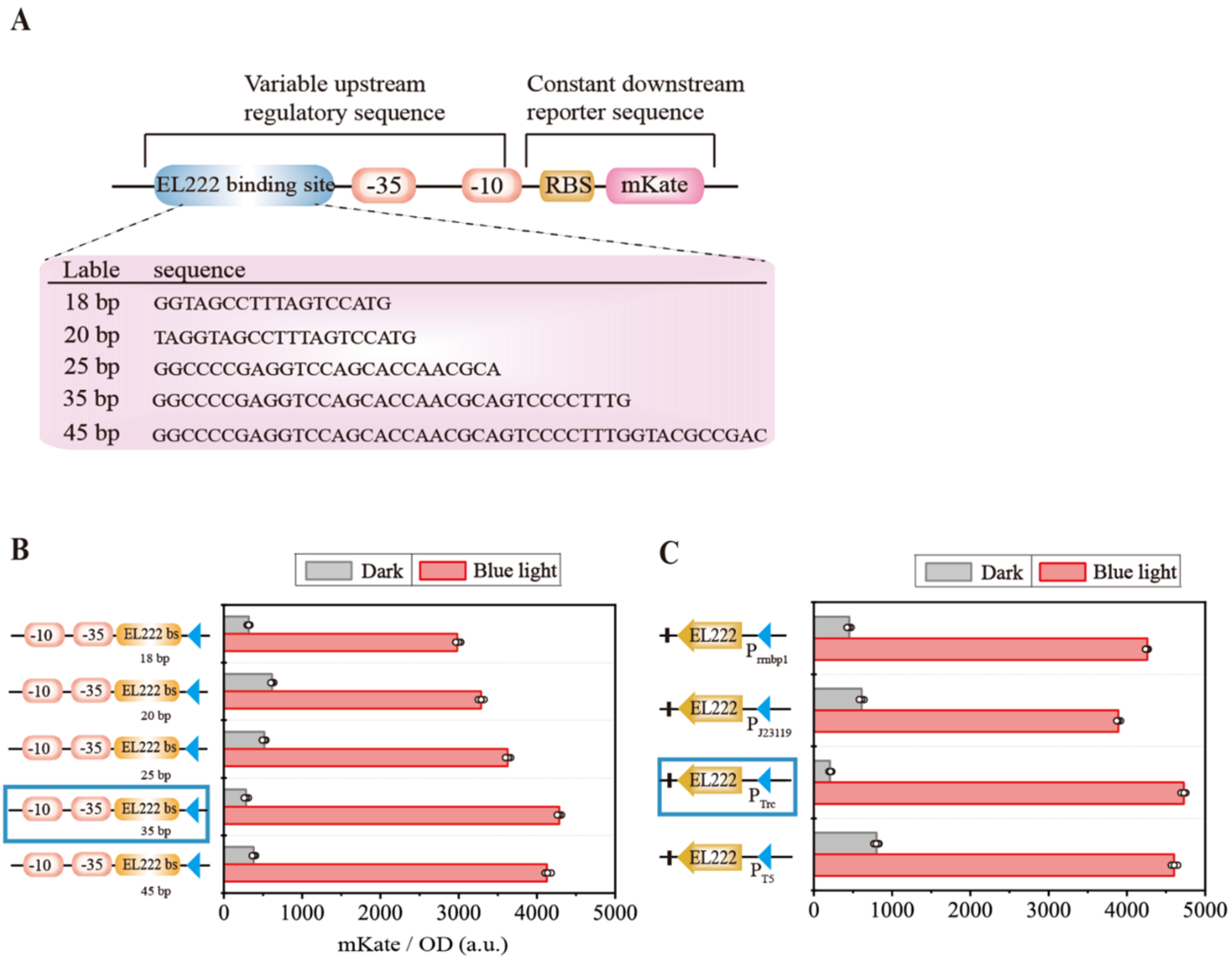

Supplementary Figure 16. Optimization of the BLAT tool. (A) Different length of EL222 binding site for the BLAT tool. **(B)** Effect of different length of EL222 binding sites on mKate expression in the BLAT tool. The blue line is the best length of EL222 binding sites for the BLAT tool. **(C)** Effect of different strength of EL222 expression on mKate expression in the BLAT tool. The blue line is the optimal expression level of EL222. For B, C, values are shown as mean ± s.d. from three (n = 3) biological independent replicates. Source data underlying Supplementary Fig. 16B and 16C are provided as a Source Data file.

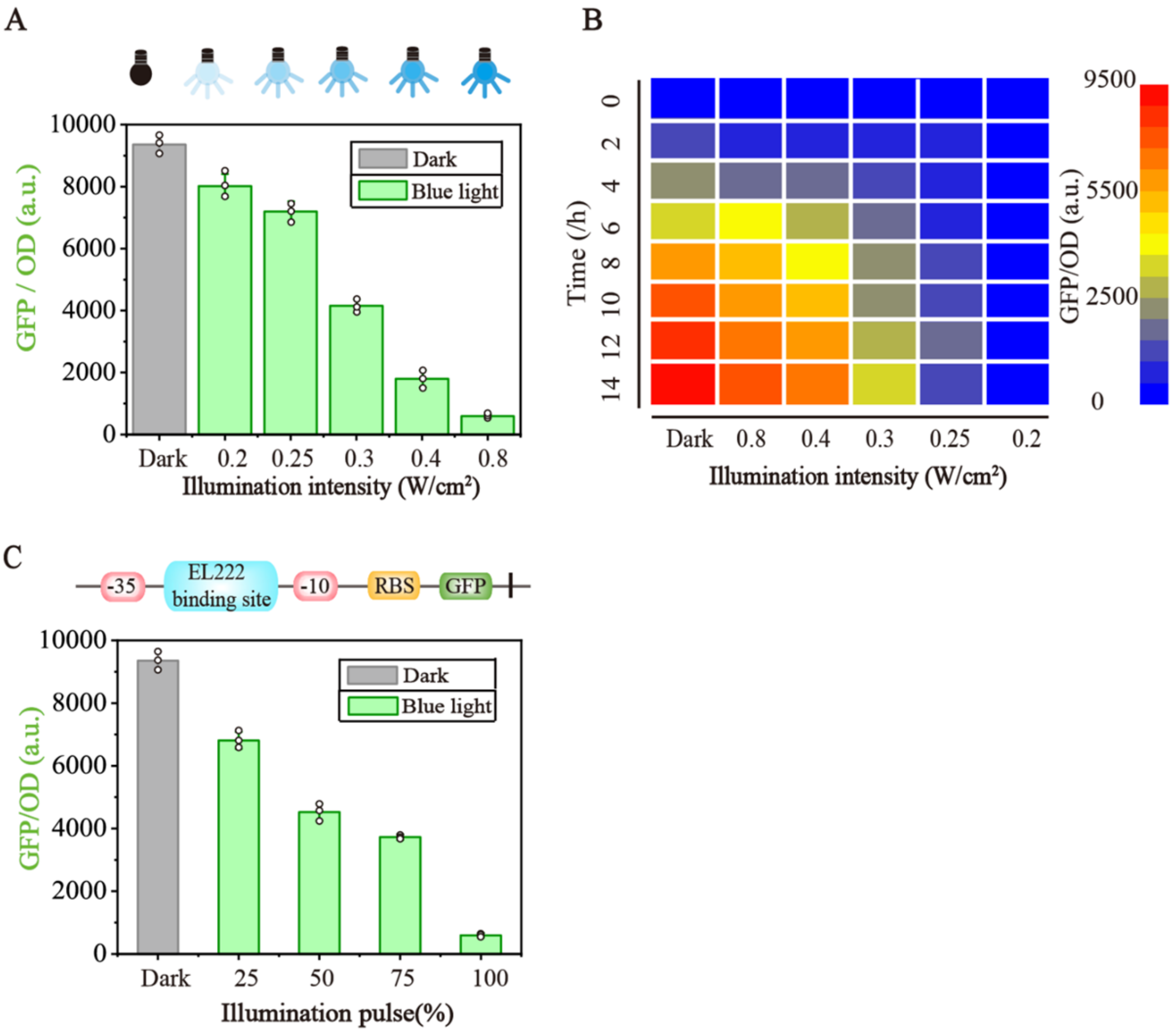

Supplementary Figure 17. The performance of the BLRT tool. (A) Dose-dependent repression with different illumination intensity (0-0.8 W/cm^2). **(B)** Dose-dependent repression with different illumination intensity and culture time (0-0.8 W/cm^2). **(C)** Dose-dependent repression with different illumination pulse (0.8 W/cm^2, 0, 25%, 50%, 75% and 100%). For A-C, values are shown as mean ± s.d. from three (n = 3) biological independent replicates. Source data are provided as a Source Data file.

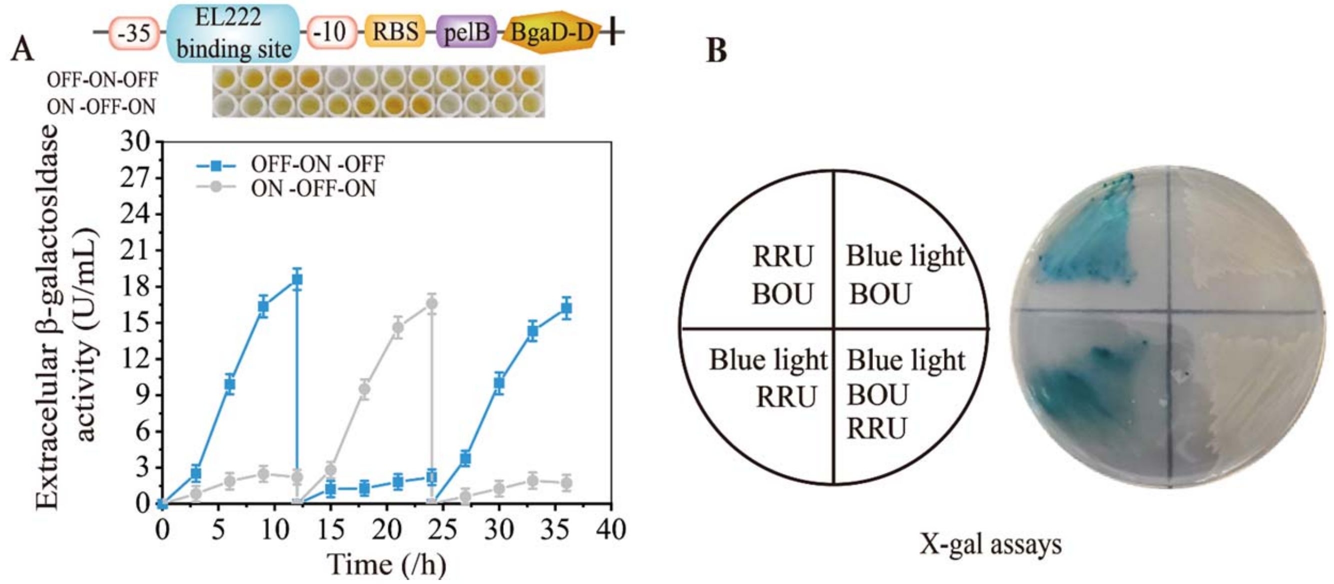

Supplementary Figure 18. The analysis of the BLRT tool. (A) Reversibility of the BLRT-triggered transgene repression with β-galactosidase by an OFF-ON-OFF / ON-OFF-ON switch. The secretroy signal peptide pelB is used to transport β-galactosidase to extracellular medium. The culture medium was refreshed every 12 h to clean the extracellular β-galactosidase. The inserted figure is the production of yellow NPG via β-galactosidase. **(B)** Converting X-gal into 5-bromo-4-indigo by β-galactosidase with BLRT regulation. For A, values are shown as mean ± s.d. from three (n = 3) biological independent replicates. Source data are provided as a Source Data file.

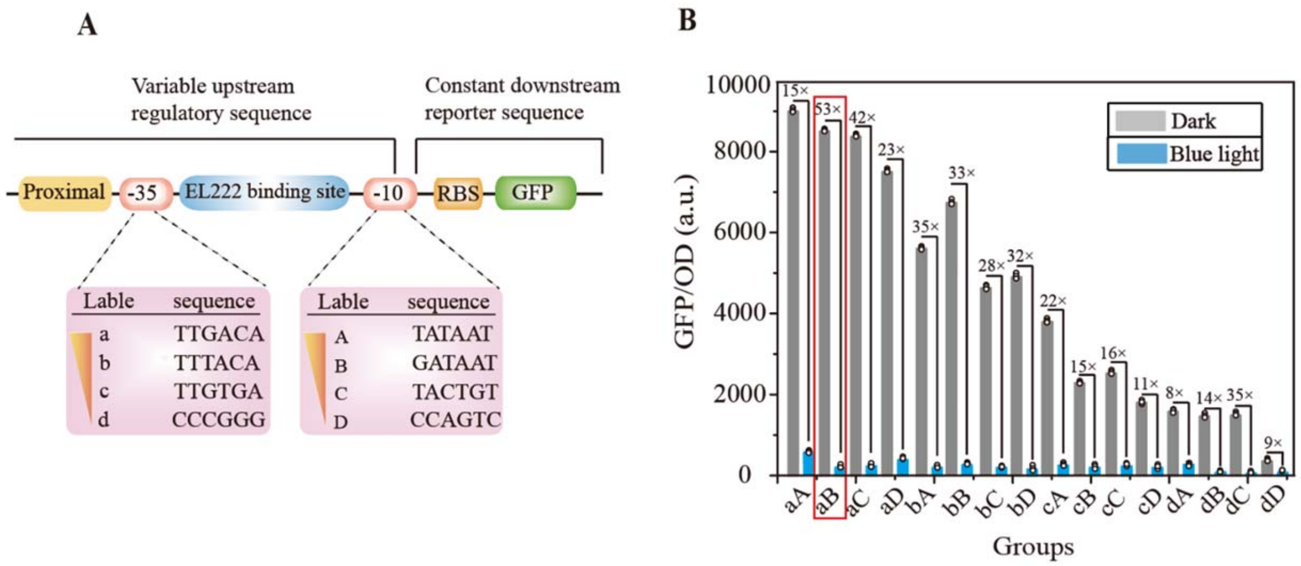

Supplementary Figure 19. Optimization of the BLRT tool. (A) Effect of the modified P_{J23119} promoter on GFP expression. **(B)** Effect of 16 groups of promoter library on the repression ratio of blue-light. The red line is the best group with blue light. **aA** is the control group. For B, values are shown as mean ± s.d. from three (n = 3) biological independent replicates. Source data underlying Supplementary Fig. 19B are provided as a Source Data file.

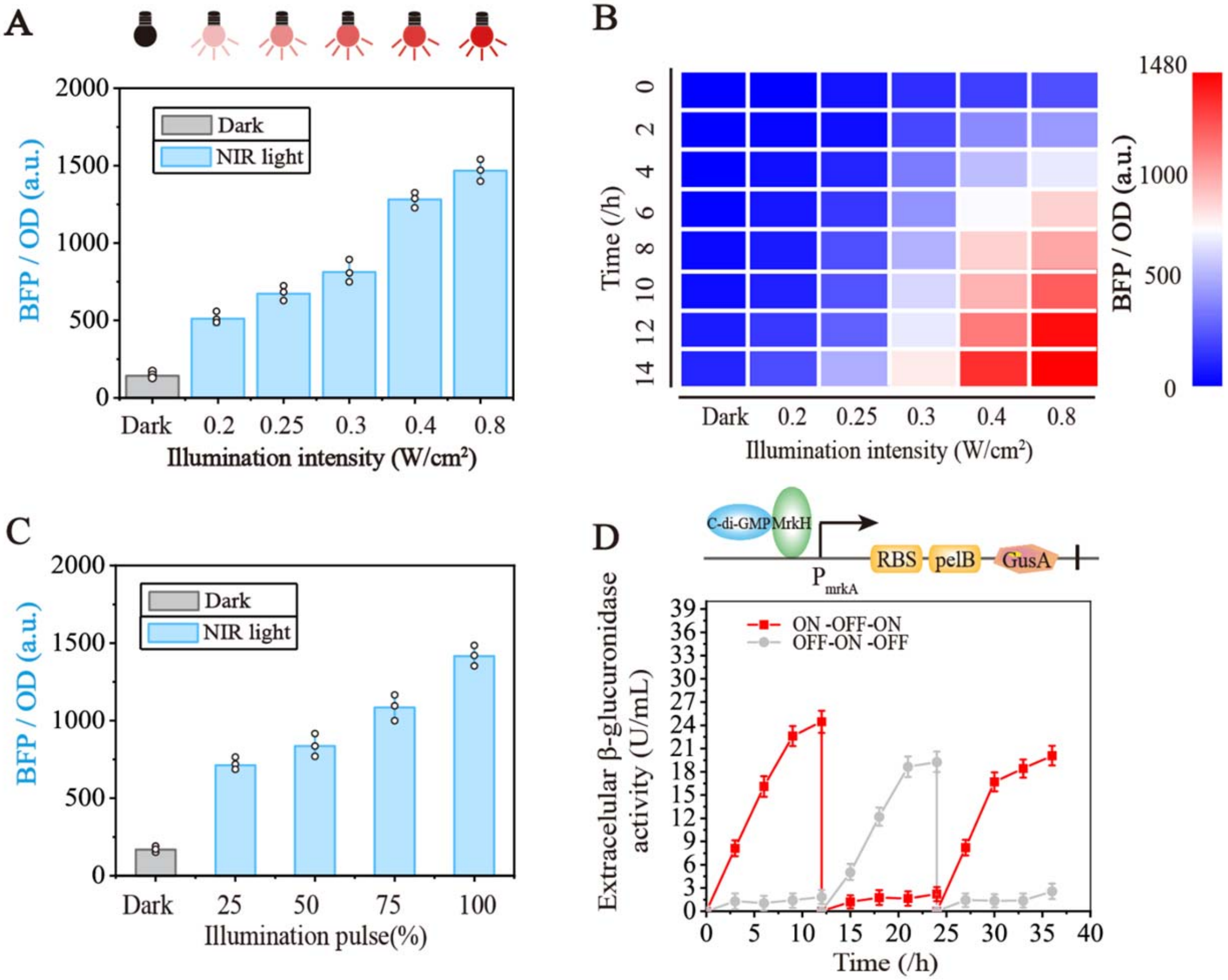

Supplementary Figure 20. The performance of the NRAT tool. (A) Dose-dependent activation with different illumination intensity (0-0.8 W/cm^2). **(B)** Dose-dependent activation with different illumination intensity and culture time (0-0.8 W/cm^2). **(C)** Dose-dependent activation with different illumination pulse (0.8 W/cm^2, 0, 25%, 50%, 75% and 100%). **(D)** Reversibility of the NRAT-triggered transgene expression with β-glucuronidase by an ON-OFF-ON/ OFF-ON-OFF switch. The secretroy signal peptide pelB is used to transport β-galactosidase to extracellular medium. The culture medium is refreshed every 12 h to clean the extracellular β-glucuronidase. For A-D, values are shown as mean ± s.d. from three (n = 3) biological independent replicates. Source data are provided as a Source Data file.

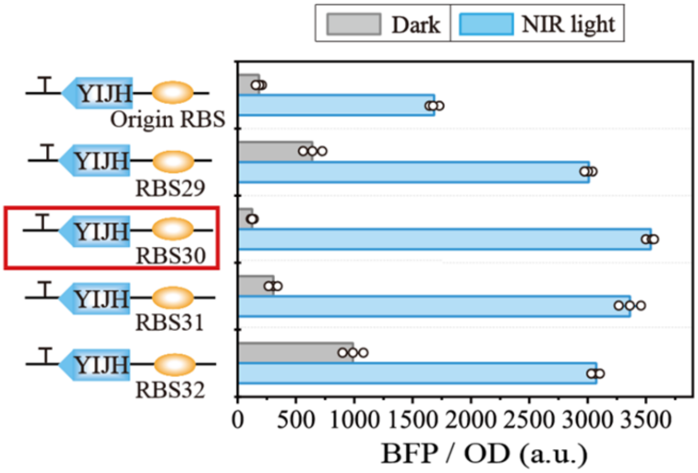

Supplementary Figure 21. Optimization of the NRAT tool. Effect of different RBS strength of *yhjh* gene on BFP expression. The red line is the best RBS strength for *yhjh* gene. Values are shown as mean ± s.d. from three (n = 3) biological independent replicates. Source data are provided as a Source Data file.

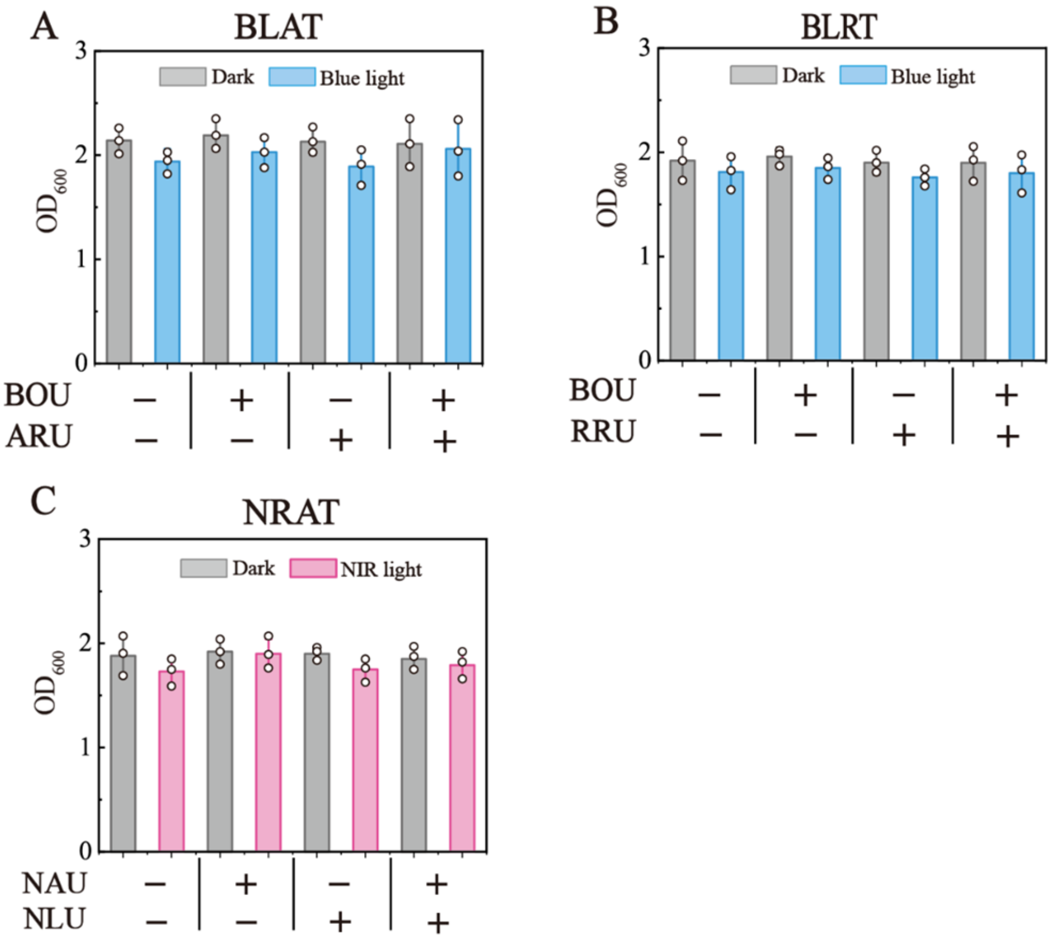

Supplementary Figure 22. Effect of the BLAT, BLRT and NRAT tools on OD. (A) Effect of the BLAT tool on OD. **(B)** Effect of the BLRT tool on OD. **(C)** Effect of the NRAT tool on OD**.** For A-C, values are shown as mean ± s.d. from three (n = 3) biological independent replicates. Source data are provided as a Source Data file.

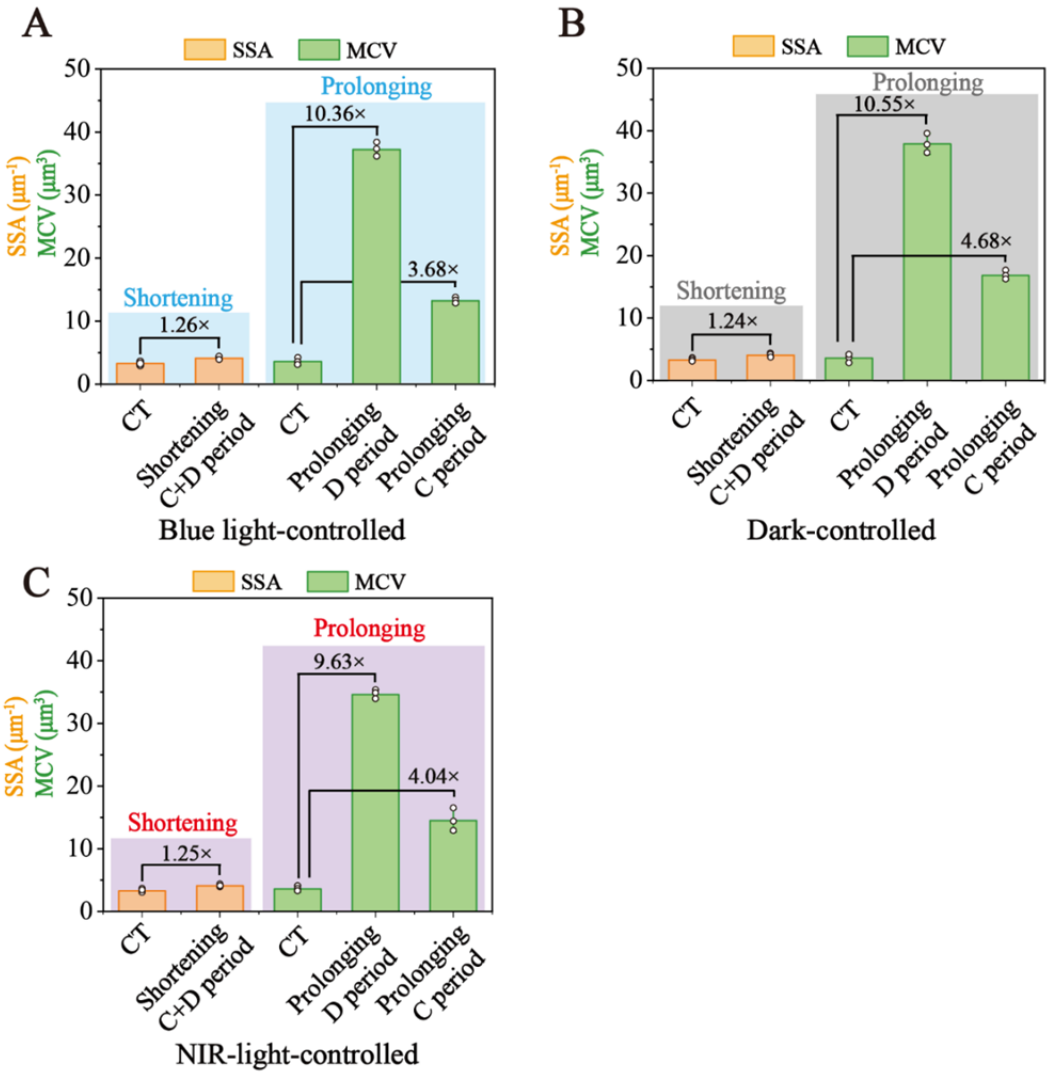

Supplementary Figure 23. Controlling the C and D periods of cell division by three optogenetics tools. (A) Controlling the C and D periods of cell division by the BLAT tool. The *nrdAB+ftsZA* and *sulA* genes were controlled by 0.8 W/cm^2 illumination intensity, respectively. The *nrdA* gene was controlled by 0.2 W/cm^2 illumination intensity. **(B)** Controlling the C and D periods of cell division by the BLRT tool. **(C)** Controlling the C and D periods of cell division by the NRAT tool. For A-C, values are shown as mean ± s.d. from three (n = 3) biological independent replicates. Source data are provided as a Source Data file.

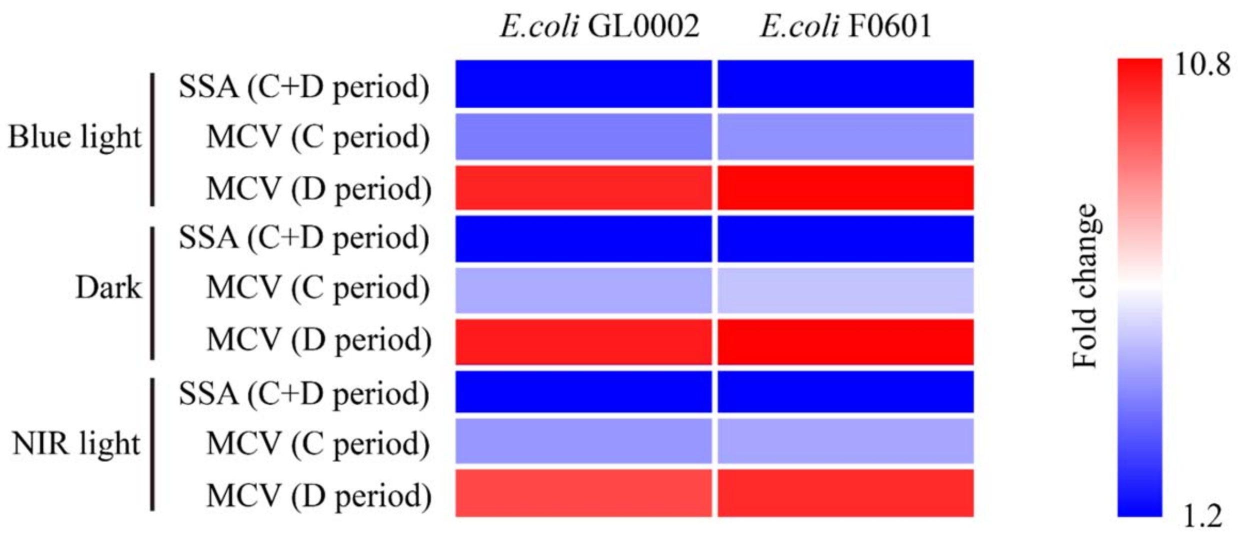

Supplementary Figure 24. Controlling the C and D periods of cell division by three optogenetics tools in *E. coli* GL0002 and *E. coli* F0601. The *nrdAB+ftsZA* and *sulA* genes were controlled by 0.8 W/cm^2 illumination intensity, respectively. The *nrdA* gene was controlled by 0.2 W/cm^2 illumination intensity. Source data are provided as a Source Data file.

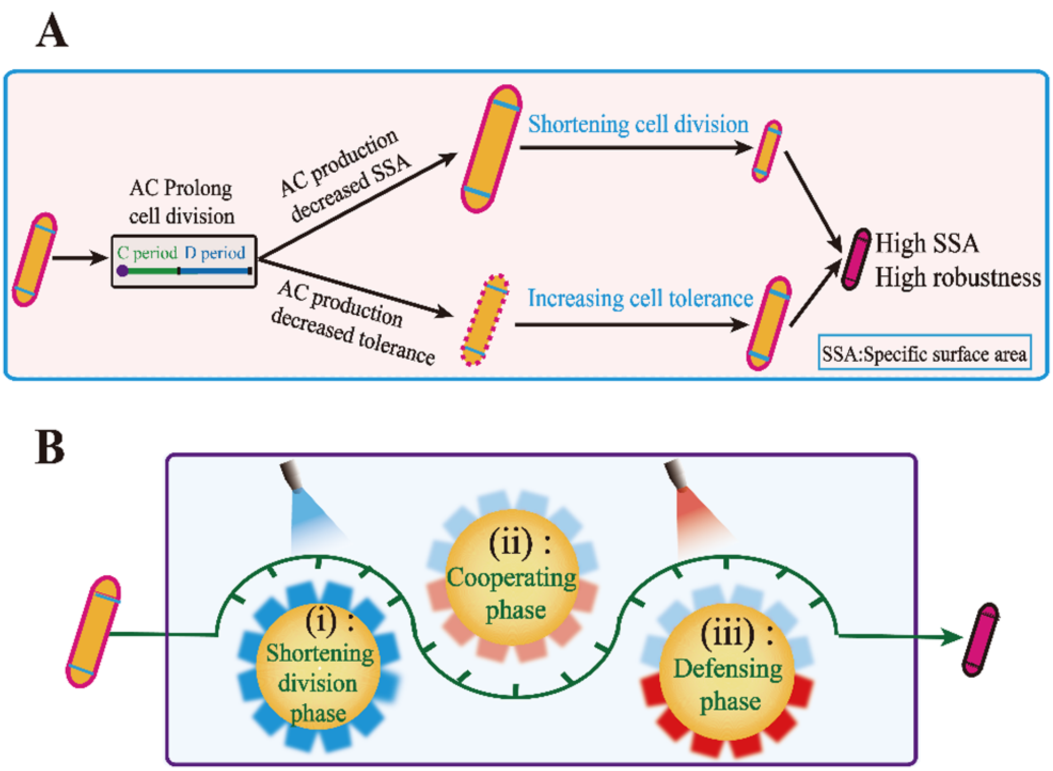

Supplementary Figure 25. Key problems and the corresponding solution in the process of acetoin production. (A) Key problems in the process of acetoin biosynthesis. **(B)** Three phases regulation strategies for acetoin production, including shortening division phase, cooperating phase, and defensing phase.

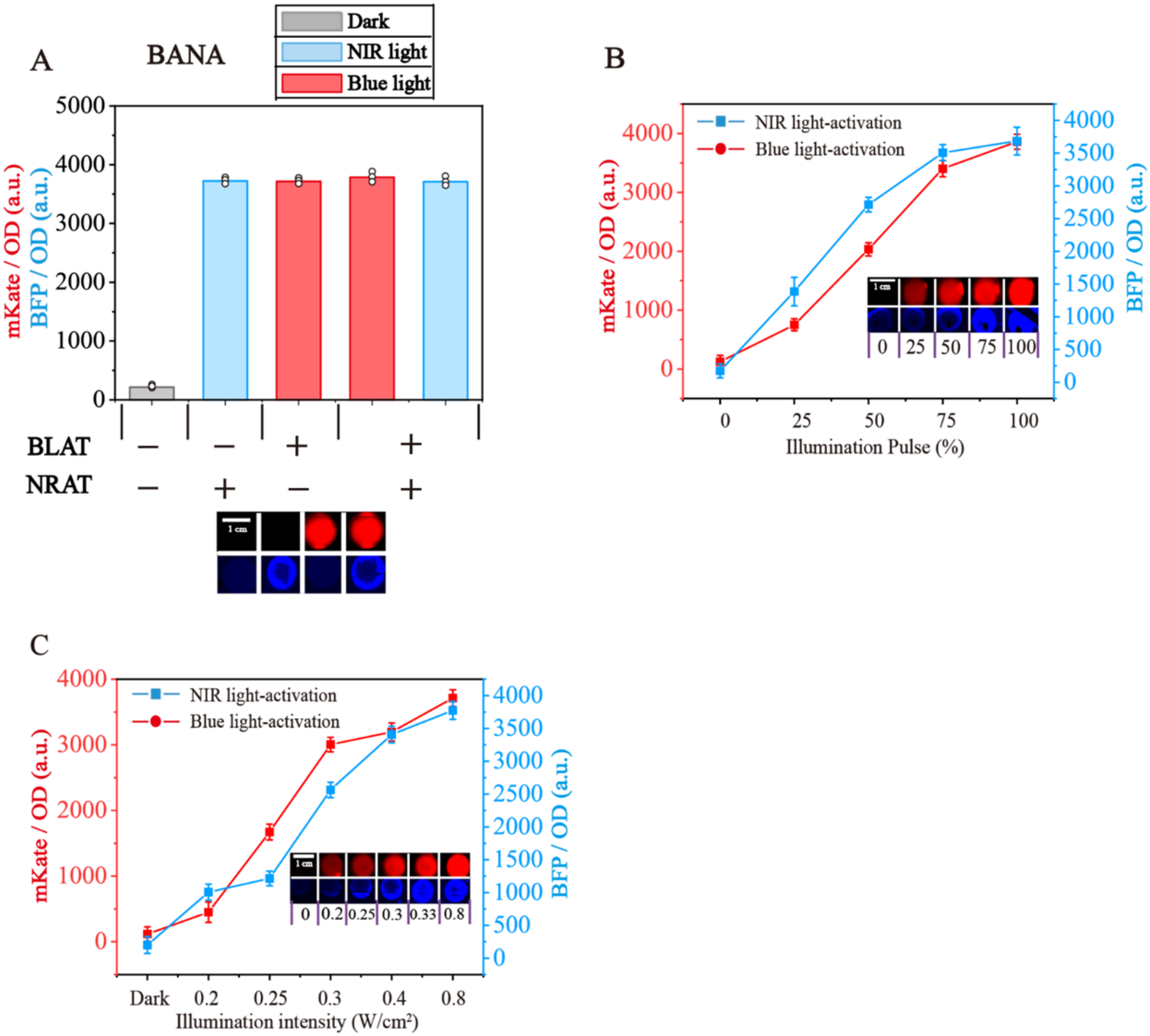

Supplementary Figure 26. The performance of the BANA system. (A) The fluorescence activation of BANA system. **(B)** Dose-dependent activation with different illumination pulse. **(C)** Dose-dependent activation with different illumination intensity. The inserted figure is the fluorescence profiles for two illumination conditions. For A-C. Scale bar, 1 cm. For A-C, values are shown as mean ± s.d. from three (n = 3) biological independent replicates. Source data are provided as a Source Data file.

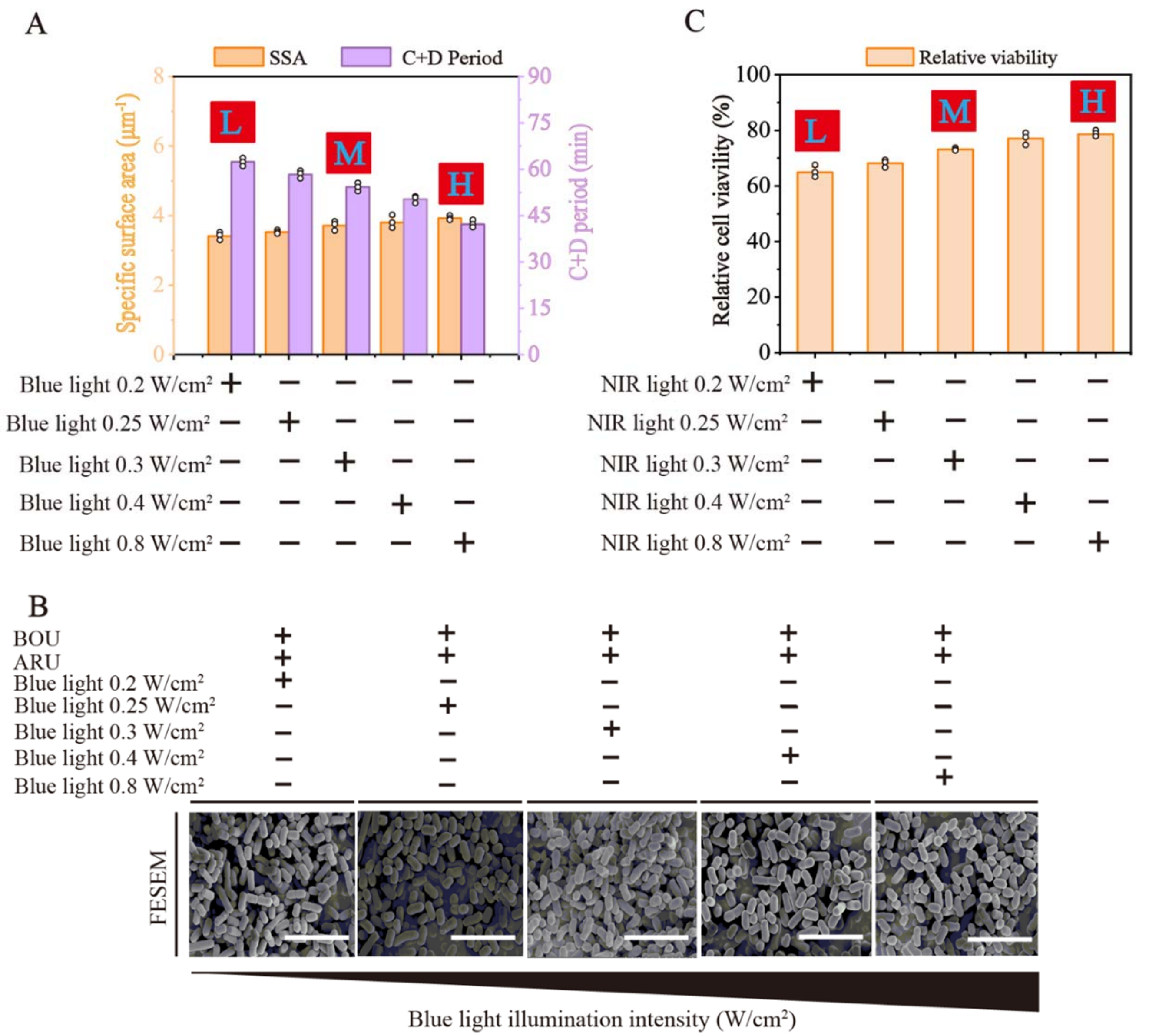

Supplementary Figure 27. The performance of shortening cell division by the BANA system. (A) Effect of blue-light on the SSA. **(B)** Effect of blue-light on cell morphology. **(C)** Effect of NIR-light on cell viability. H is 0.8 W/cm^2 (High SSA or high relative cell viability); M is 0.3 W/cm^2 (Moderating SSA or high relative cell viability); L is 0.2 W/cm^2 (Low SSA or high relative cell viability). For B. Scale bar is 5 μm. For A, C, values are shown as mean ± s.d. from three (n = 3) biological independent replicates. Source data are provided as a Source Data file.

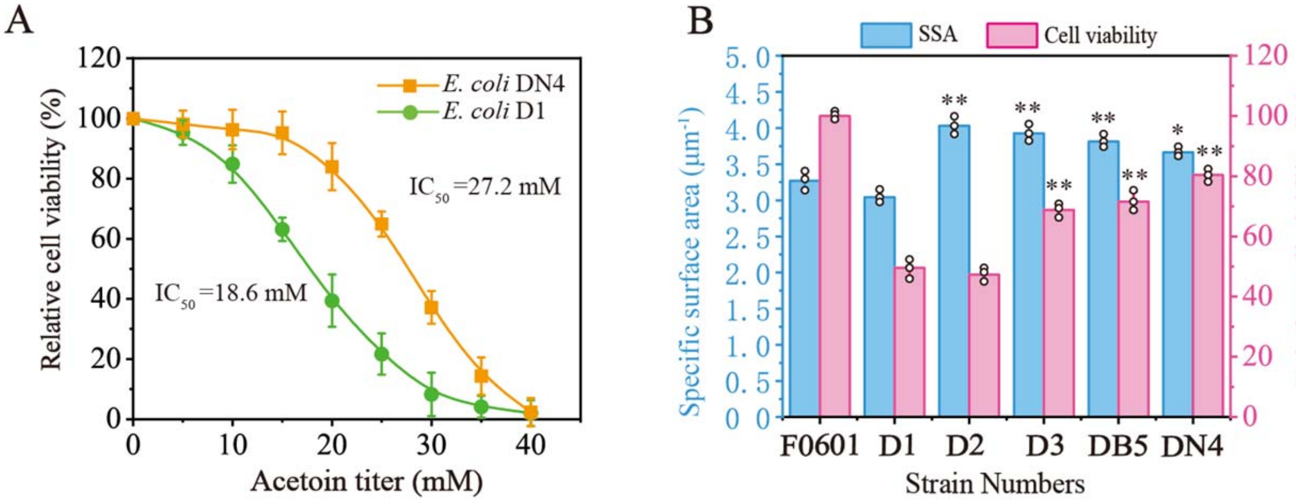

Supplementary Figure 28. Effect of the BANA system on the specific surface area and cell viability. (A) Effect of acetoin titer on cell viability. IC50, half maximal inhibitory of *E. coli* D1 and *E. coli* DN4. **(B)** Effect of the BANA system on SSA and cell viability in different strains. The relative cell viability was normalized to *E. coli* F0601. For A, B, values are shown as mean ± s.d. from three (n = 3) biological independent replicates. Significance (*p*-value) was evaluated by two-sided t-test (*, p<0.05; **, p<0.01; ***, p<0.001), compared to *E. coli* D1. Source data are provided as a Source Data file.

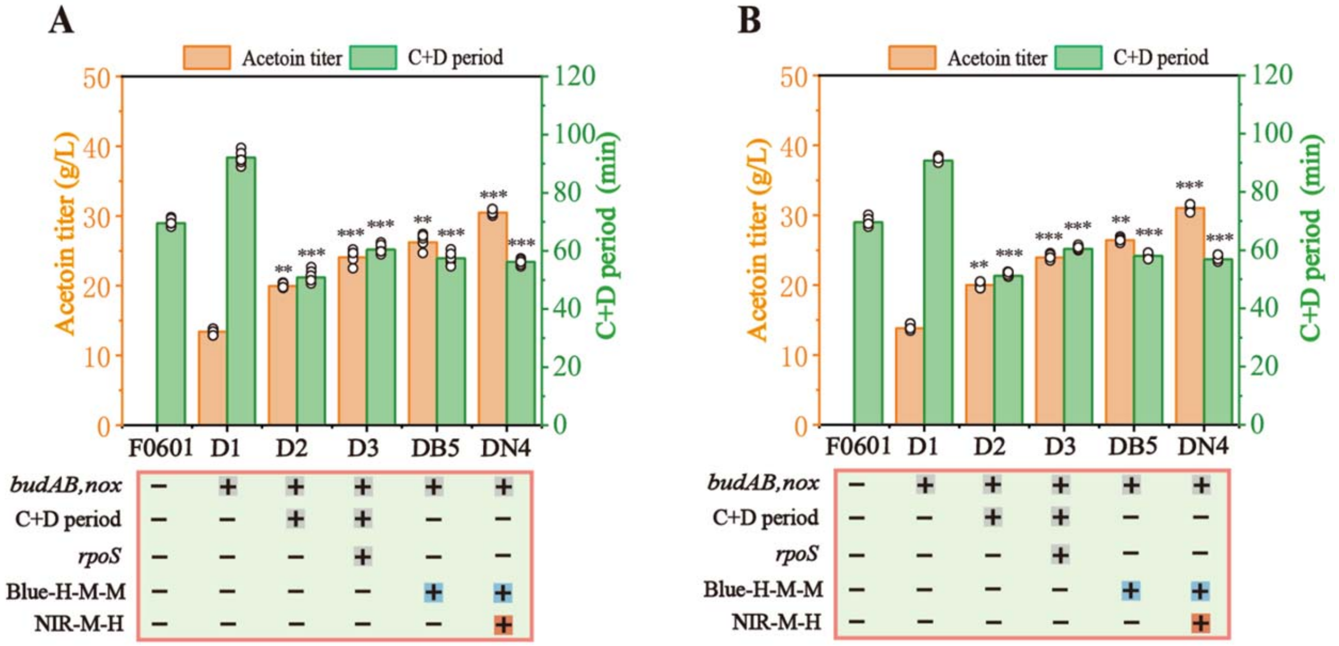

Supplementary Figure 29. The independent repetitions of acetoin production. (A) The first round repetition of acetoin production. **(B)** The second round repetition of acetoin production. For A, B, values are shown as mean ± s.d. from three (n = 6) biological independent replicates. Significance (*p*-value) was evaluated by two-sided t-test (*, p<0.05; **, p<0.01; ***, p<0.001), compared to *E. coli* D1. Source data are provided as a Source Data file.

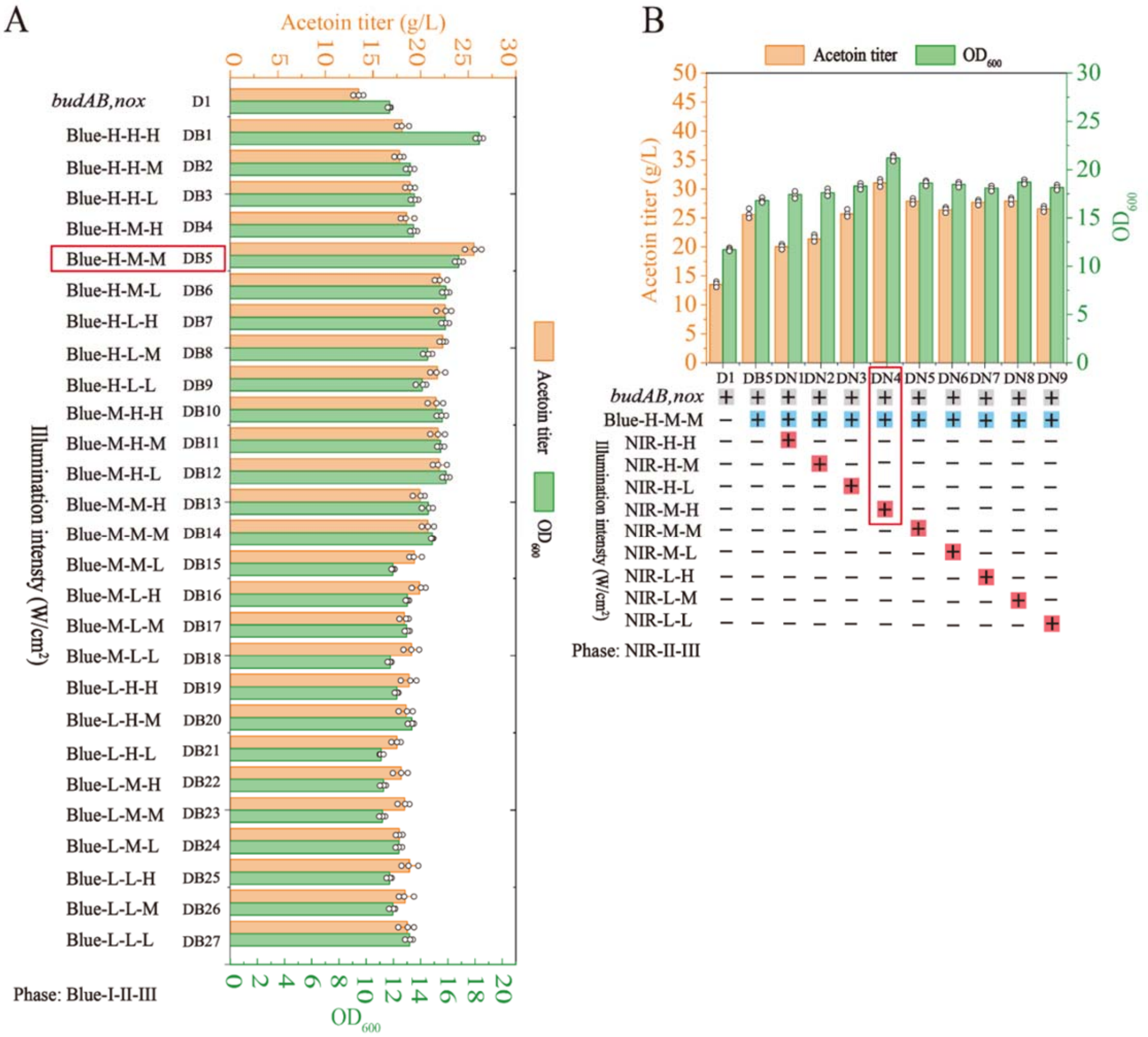

Supplementary Figure 30. Optimization of the Blue and NIR light illumination intensity for acetoin production. **(A)** Optimization of blue light illumination intensity. Blue light illumination intensity in three phases was optimized under three different levels: high (H, 0.8 W/cm^2), moderate (M, 0.3 W/cm^2), and low (L, 0.2 W/cm^2). **(B)** Optimization of NIR illumination intensity. NIR light illumination in two phases was optimized under three different levels: high (H, 0.8 W/cm^2), moderate (M, 0.3 W/cm^2), and low (L, 0.2 W/cm^2). The corresponding illustration was provided in Supplementary Note 6. For A, B, values are shown as mean ± s.d. from three (n = 3) biological independent replicates. Source data are provided as a Source Data file.

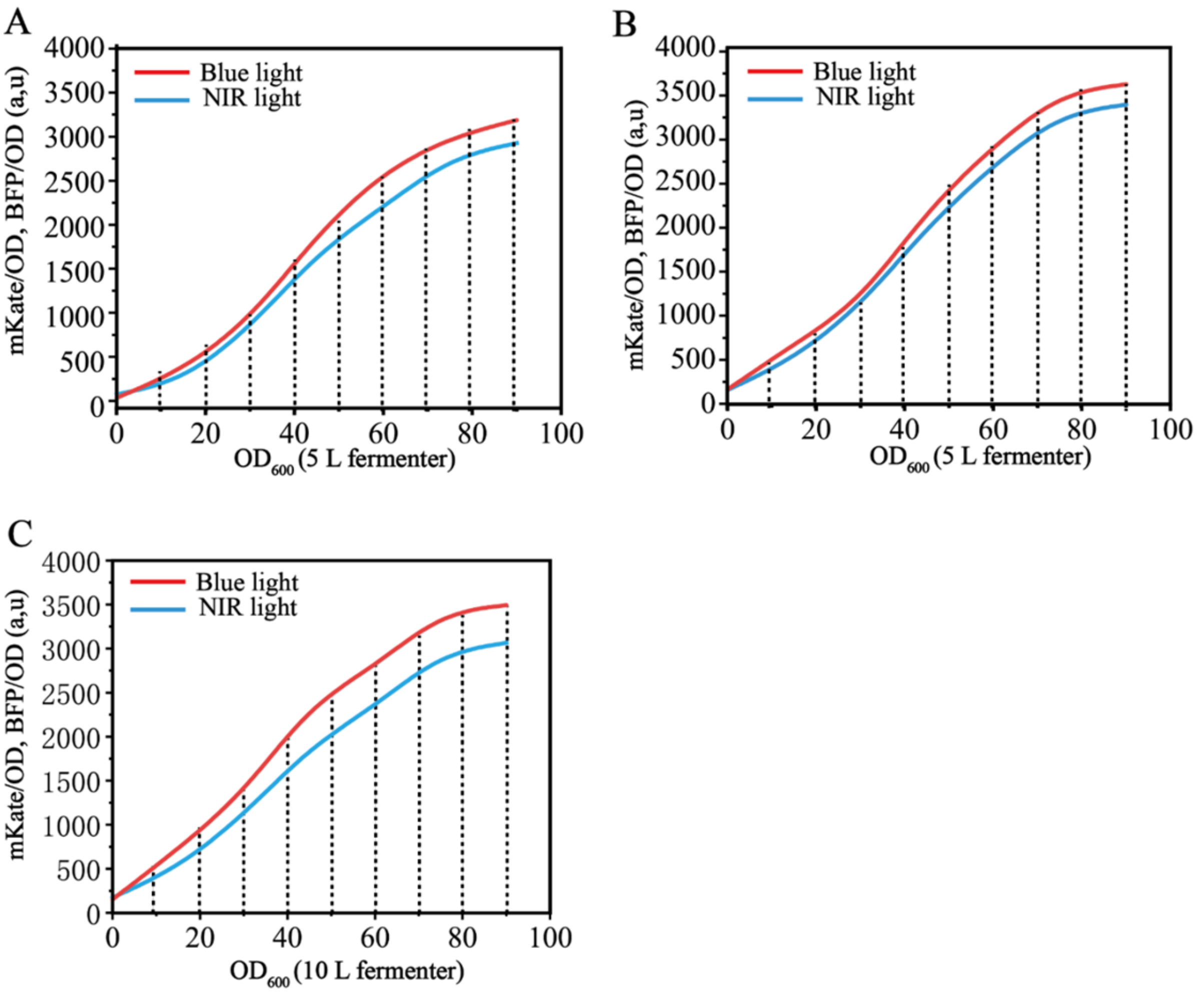

Supplementary Figure 31. Effect of BANA on genes expression in 5-L and 10-L fermenters. (A) Effect of BANA with one blue light and one NIR light illumination sources on genes expression in 5-L fermenter. **(B)** Effect of BANA with two blue light and two NIR light illumination sources on genes expression in 5-L fermenter. **(C)** Effect of BANA with two blue light and two NIR light illumination sources on genes expression in 10-L fermenter. The corresponding illustration was introduced in the Supplementary Note 4. For A-C, values are shown as mean ± s.d. from three (n = 3) biological independent replicates. Source data are provided as a Source Data file.

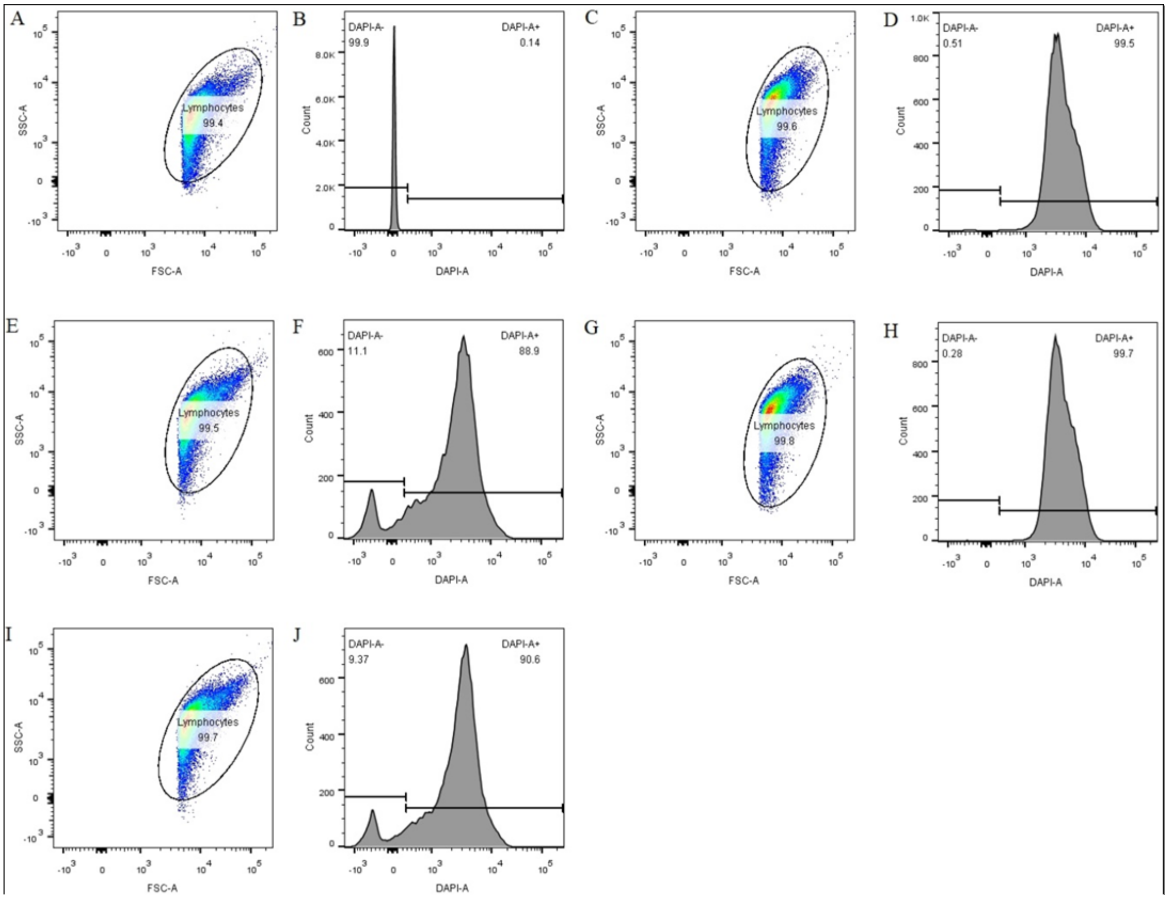

Supplementary Figure 32. Effect of fermenter scale on BFP fluorescence density. (A) and **(B)** Effect of 250 mL shake flasks on BFP fluorescence density under dark condition. **(C)** and **(D)** Effect of 250 mL shake flasks on BFP fluorescence density under one blue light and one NIR light illumination sources. **(E)** and **(F)** Effect of 5-L fermenter on BFP fluorescence density under one blue light and one NIR light illumination sources. **(G)** and **(H)** Effect of 5-L fermenter on BFP fluorescence density under two blue light and two NIR light illumination sources. **(I)** and **(J)** Effect of 10-L fermenter on BFP fluorescence density under two blue light and two NIR light illumination sources. Flow Cytometry analysis was added in the Methods (**Flow cytometry assays**). The corresponding illustration was introduced in the Supplementary Note 4.

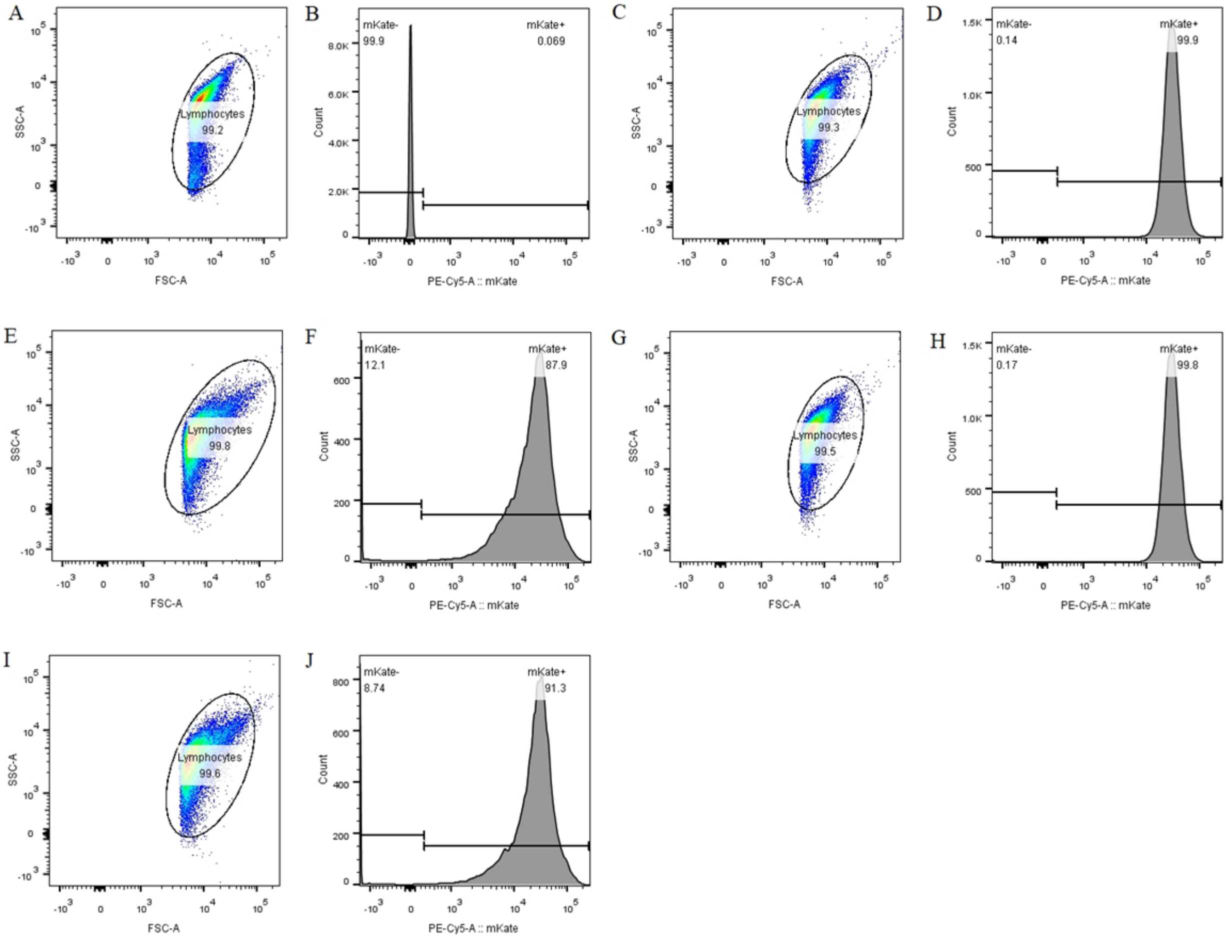

Supplementary Figure 33. Effect of fermenter scale on mKate fluorescence density. (A) and **(B)** Effect of 250 mL shake flasks on mKate fluorescence density under dark condition. **(C)** and **(D)** Effect of 250 mL shake flasks on mKate fluorescence density under one blue light and one NIR light illumination sources. **(E)** and **(F)** Effect of 5-L fermenter on mKate fluorescence density under one blue light and one NIR light illumination sources. **(G)** and **(H)** Effect of 5-L fermenter on mKate fluorescence density under two blue light and two NIR light illumination sources. **(I)** and **(J)** Effect of 10-L fermenter on mKate fluorescence density under two blue light and two NIR light illumination sources. Flow Cytometry analysis was added in the Methods (**Flow cytometry assays**). The corresponding illustration was introduced in the Supplementary Note 4.

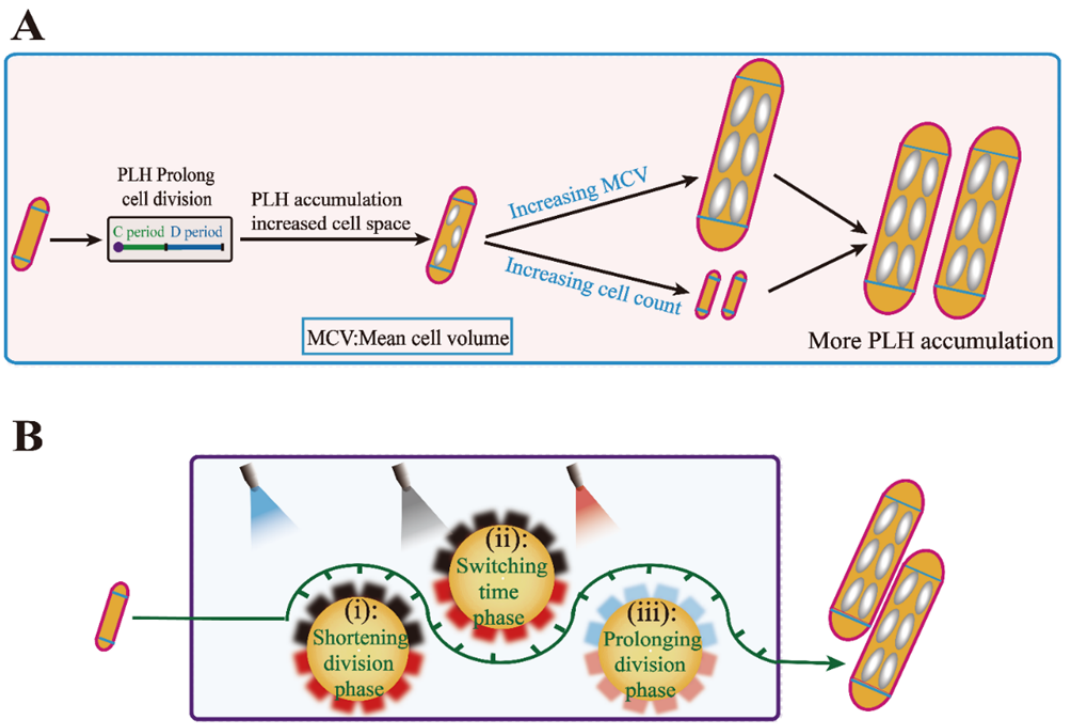

Supplementary Figure 34. Key problems and the corresponding solution in the process of PLH production. (A) Key problems in the process of PLH biosynthesis. **(B)** Three phases regulation strategies for PLH production, including shortening division phase, switching time phase, and prolonging division phase.

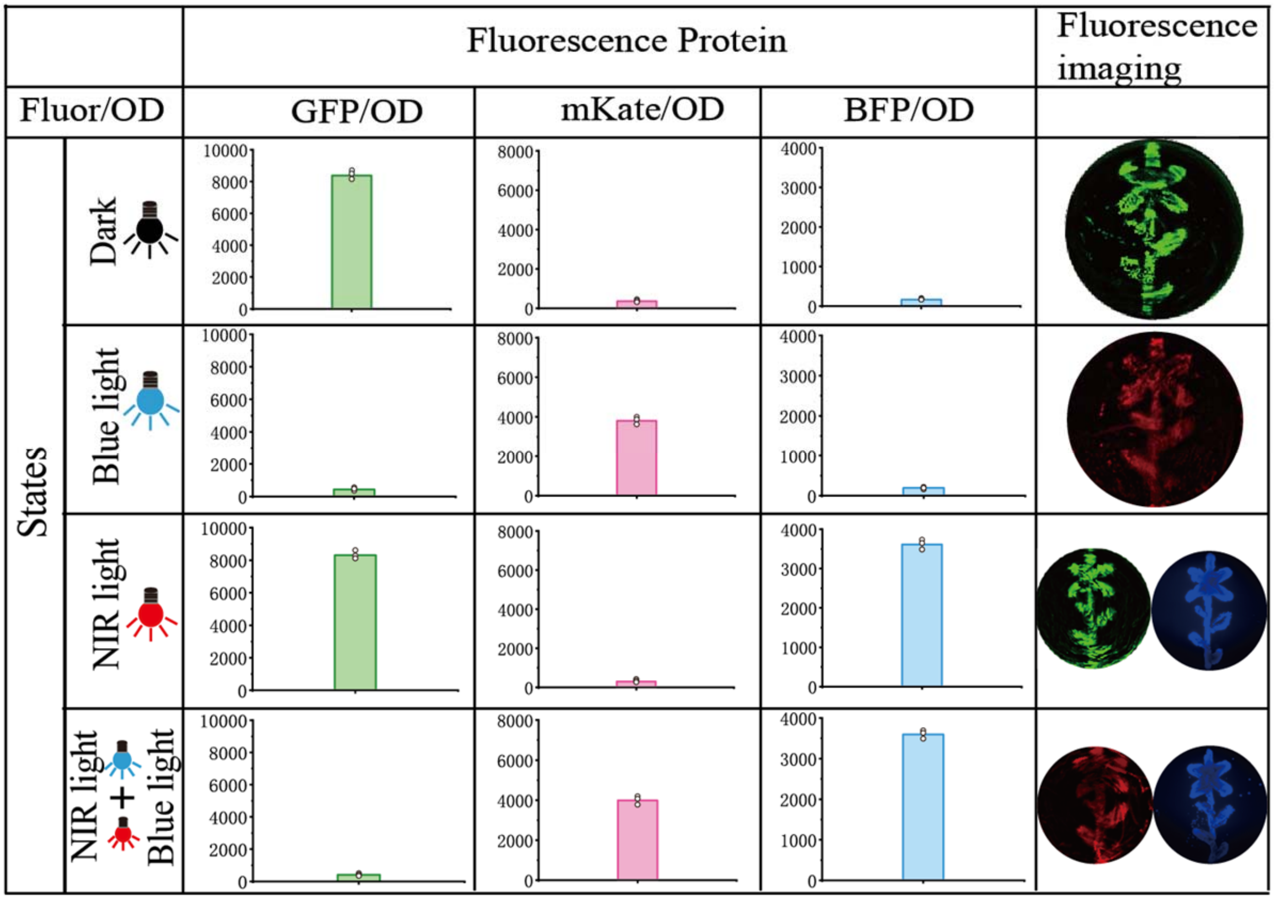

Supplementary Figure 35. The performances of the BARNA system. Effect of blue- and NIR-light (0.8 W/cm^2) on the GOI expression. The inserted figure is the bioimaging for different color Flowers. The detail methods were added in the method (**The bioimage of agarose plates**). For A, values are shown as mean ± s.d. from three (n = 3) biological independent replicates. Source data are provided as a Source Data file.

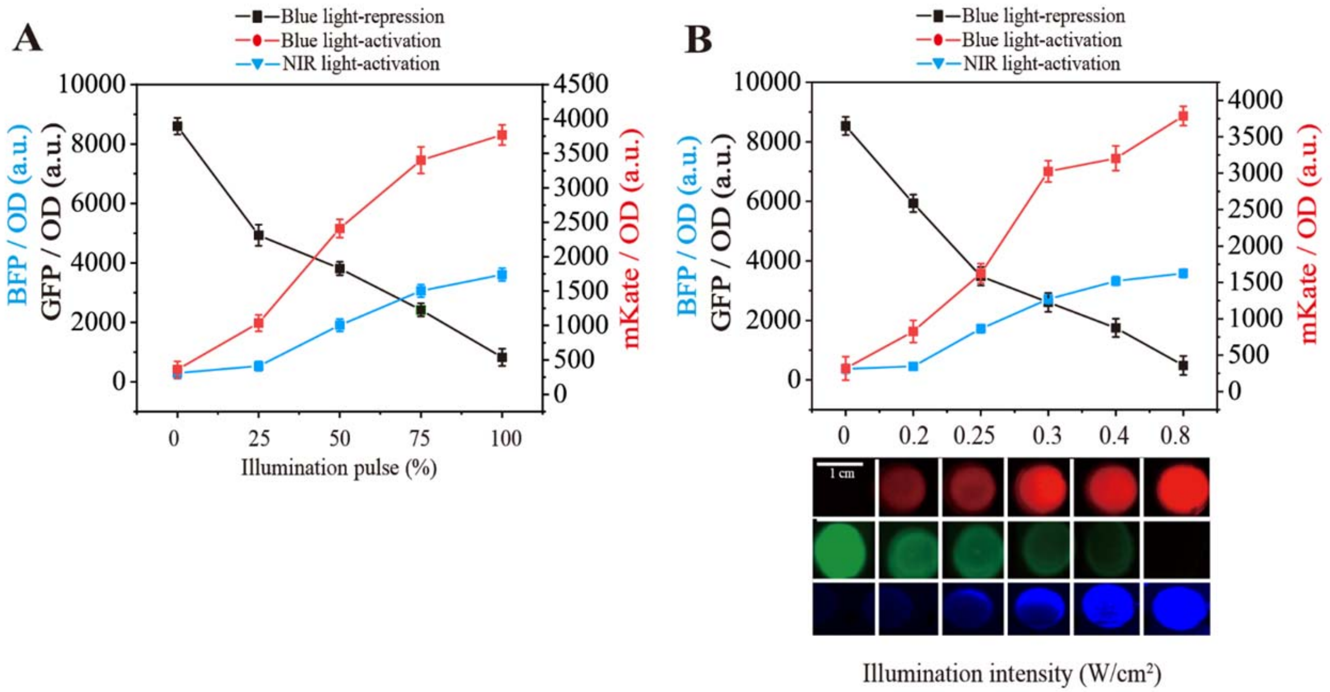

Supplementary Figure 36. Effect of the BARNA system on genes expression. (A) Dose-dependent activation and repression with different illumination pulse. **(B)** Fluorescence regulation with blue-and NIR-light. The inserted figures are the fluorescence profiles for different conditions. For A, B, Scale bar is 1 cm. For A, B, values are shown as mean ± s.d. from three (n = 3) biological independent replicates. Source data are provided as a Source Data file.

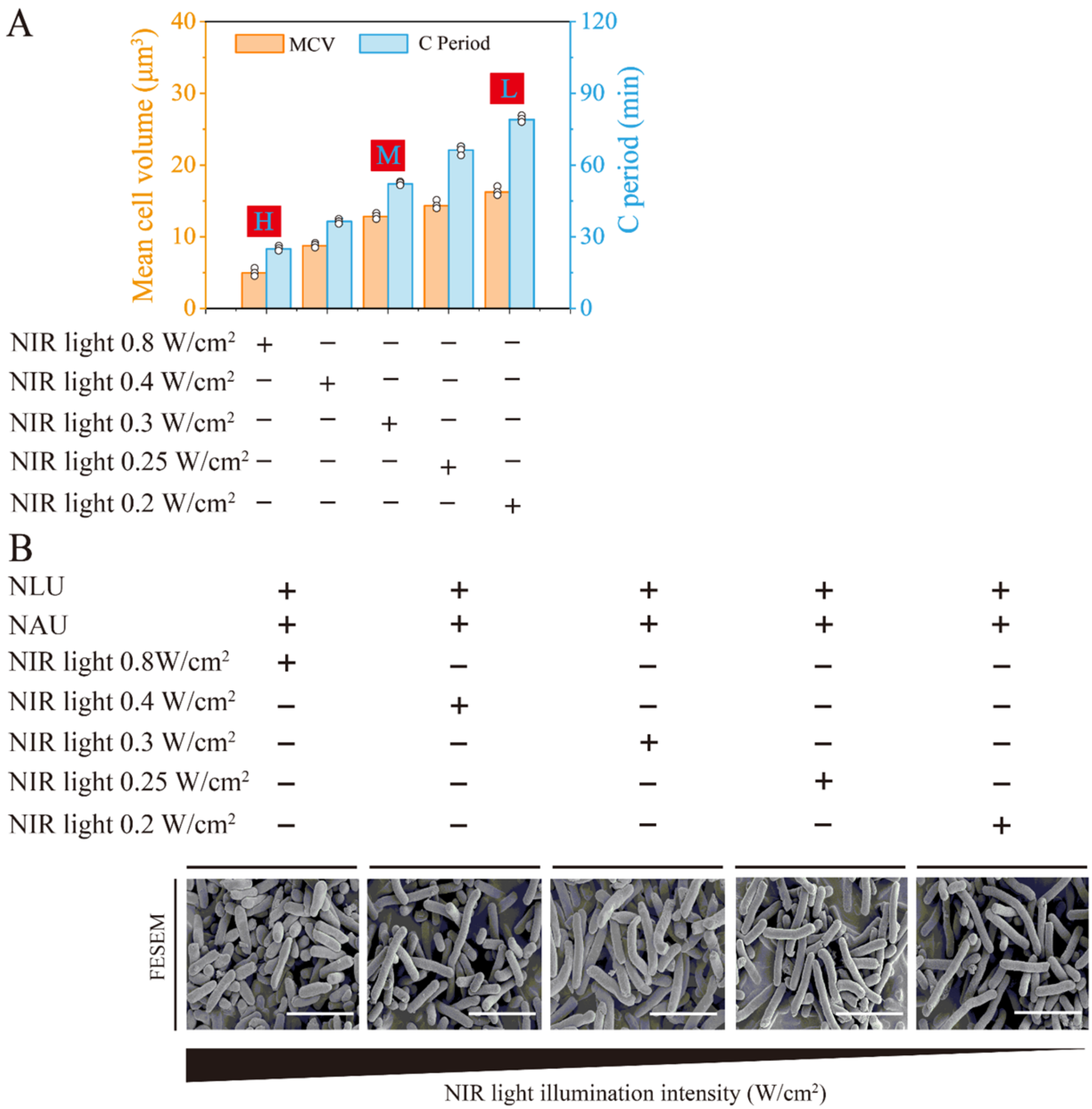

Supplementary Figure 37. The performance of prolonging the C period by the BARNA system. (A). Effect of NIR-light on the MCV. **(B)** Effect of NIR-light on the MCL. H is 0.8 W/cm^2 (Low MCV); M is 0.3 W/cm^2 (Moderate MCV); L is 0.2 W/cm^2 (High MCV). For B, Scale bar is 5 μm. For A, values are shown as mean ± s.d. from three (n = 3) biological independent replicates. Source data are provided as a Source Data file.

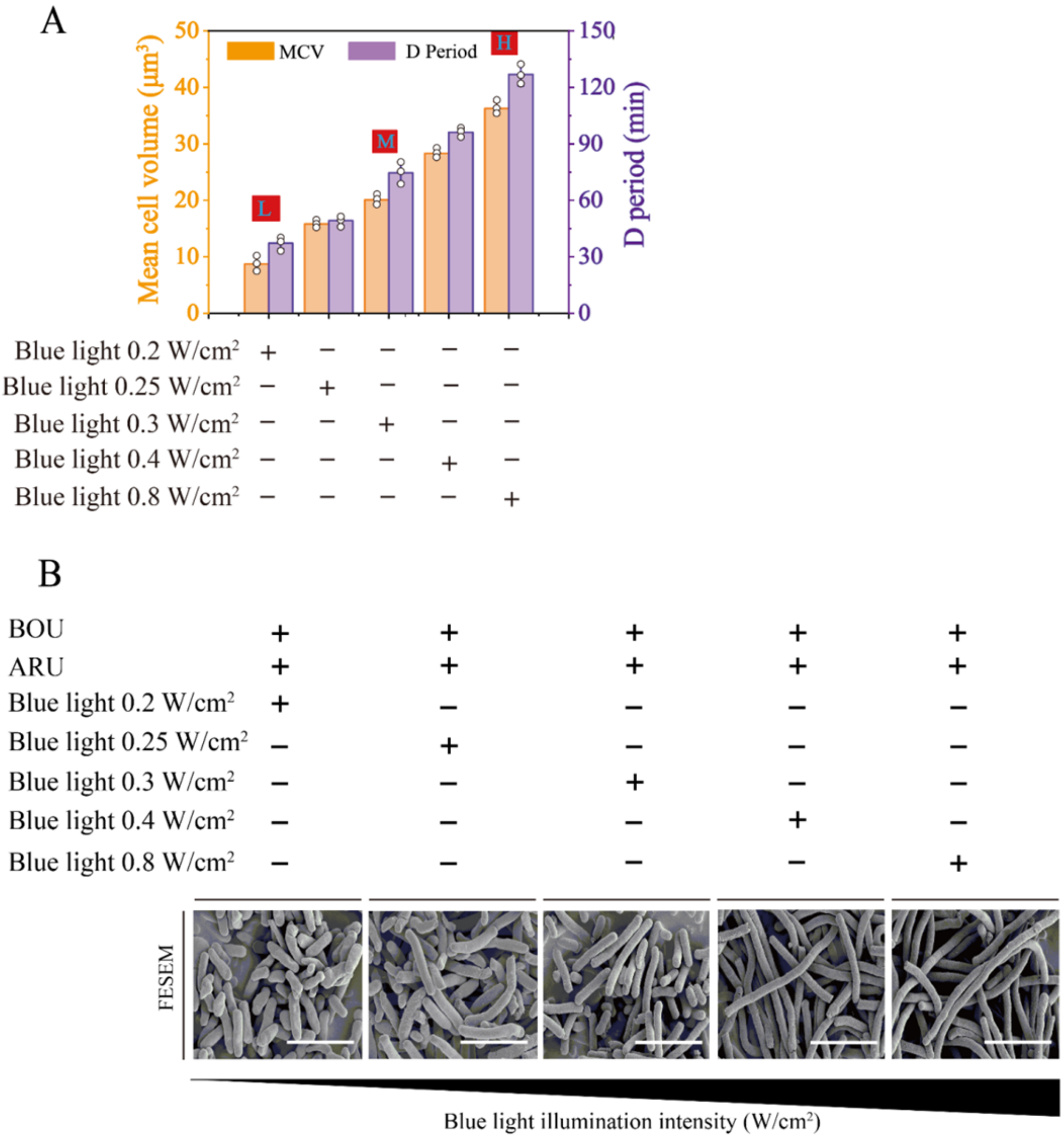

Supplementary Figure 38. The performance of prolonging the D period by the BARNA system. (A) Effect of blue-light on the MCV. **(B)** Effect of blue-light on the MCL. H is 0.8 W/cm^2 (High MCV); M is 0.3 W/cm^2 (Moderate MCV); L is 0.2 W/cm^2 (Low MCV). For A, values are shown as mean ± s.d. from three (n = 3) biological independent replicates. For B, Scale bar is 5 μm. Source data are provided as a Source Data file.

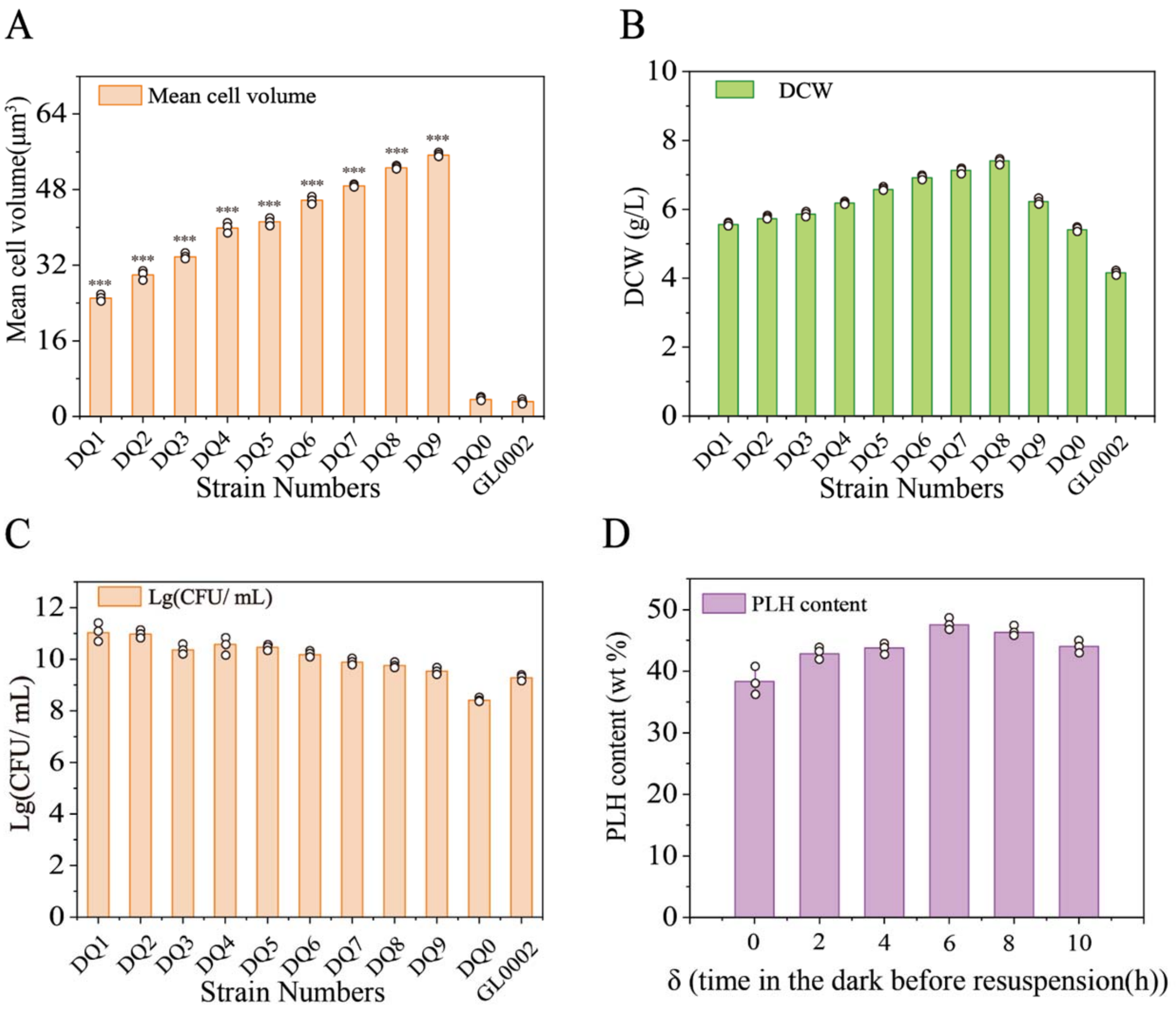

Supplementary Figure 39. Effect of blue and NIR light combination on cell morphology. (A) Effect of blue and NIR light combination on mean cell volume. **(B)** Effect of blue and NIR light combination on dry cell weight (DCW) . **(C)** Effect of blue and NIR light combination on Lg (CFU/mL). **(D)** Effect of switching time on PLH content For A-D, values are shown as mean ± s.d. from three (n = 3) biological independent replicates. Significance (*p*-value) was evaluated by two-sided t-test (*, $p<0.05$; **, $p<0.01$; ***, $p<0.001$), compared to *E. coli* DQ0. Source data are provided as a Source Data file.

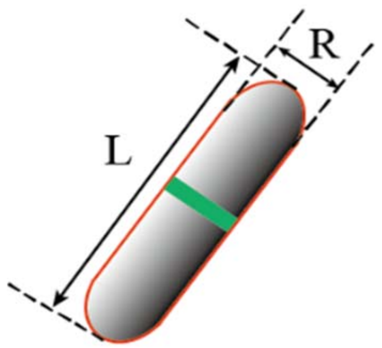

Supplementary Figure 40. The model of *E. coli*. This model of *E. coli* was mainly used to calculate the mean cell width and mean cell length.

Supplementary Table 1. Primers used in this study.

Names	Sequence (5′–3′)	purpose
YZ-tet-S	CCTTCGATTCCGACCTCAT	DNA fragment inserting confirmation
YZ-tet-A	GTTCACCGACAAACAACAGATA	DNA fragment inserting confirmation
YZ-pBLind-v1-S	CGAACTGAGATACCTACAGCG	DNA fragment inserting confirmation
YZ-pBLind-v1-A	TAACAGGGTAATCCATGGAGATCT	DNA fragment inserting confirmation
YZ-pBLrep-v1-S	AGCCCAGCTTGGAGCGAACG	DNA fragment inserting confirmation
YZ-pBLind-v1-A	GTAGGGATAACAGGGTAATAG	DNA fragment inserting confirmation
YZ-P_{J23119}-S	GCATGCAAGGAGATGGCG	DNA fragment inserting confirmation
YZ-P_{J23119}-A	ATCCGGATATAGTTCCTCCTTTCA	DNA fragment inserting confirmation
KZ-*ftsZA*-R-S	TAGTCCATGTATAATTGAATTCTCTAGAAAAGAGGAGAAAACGCGTATGTTTGAACCAATGGAAC	*ftsZA* genes cloning in pBLrep-v1
KZ-*ftsZA*-R-A	GGATAACAGGGTAATCCATGGAGATCTTTAAAACTCTTTTCGCAGCCAA	*ftsZA* genes cloning in pBLrep-v1
KZ-*sulA*-I-S	TGAATTCTCTAGAAAAGAGGAGAAAACGCGTATGTACACTTCAGGCTATGCACATC	*sulA* gene cloning in pBLind-v1
KZ-*sulA*-I-A	CAGGGTAATAGATCTAAGCTTTTAATGATACAAATTAGAGTGAATTTTTAGC	*sulA* gene cloning in pBLind-v1
KZ-*nrdA*-M-S	CGACTAGTAAAGAGGAGAAAACGCGTATGAATCAGAATCTGCTGGTGA	*nrdA* gene cloning in P_{mrkA}
KZ-*nrdA*-M-A	GAAAGGCCCACCCGAAGGTGAGCCTCAGAGCTGGAAGTTACTCAA	*nrdA* gene cloning in P_{mrkA}
KZ-*nrdAB*-R-S	ATGTATAATTGAATTCTCTAGAAAAGAGGAGAAAACGCGTATGAATCAGAATCTGCTGGTGA	*nrdAB* genes cloning in pBLrep-v1
KZ-*nrdAB*-R-A	AGGGTAATAGATCTAAGCTTTCAGAGCTGGAAGTTACTCAA	*nrdAB* genes cloning in pBLrep-v1
KZ-*budAB*-R-S	CACCATCACCATCACGGATCCATGAACGAAAAACACGGGTGTT	*budAB* genes cloning in P_{J23119}
KZ-*budAB*-R-A	TCGACCCGGGGTACCGAGCTCTTAAATCATCTGGCTGAAGTTCATCT	*budAB* genes cloning in P_{J23119}
KZ-*nox*-S	AGCCAGATGATTTAAGAGCTCAAAGAGGAGAAAACGCGTATGAAAATCATTAGCATTAAATTCGTG	*nox* gene cloning in P_{J23119}
KZ-*nox*-A	TCTTTACCAGACTCGAGAAGCTTCGCATTCACGCTCTGCGCA	*nox* gene cloning in P_{J23119}
KZ-*phaAB*-S	ATGAATTCGAGCTCCGTCGACTTCACACAGGAAACAGAATTCATGACAGACGTGGTGATTGTTAGC	*phaAB* genes cloning in P_{J23119}
KZ-*phaAB*-A	CTCGAGTGCGGCCGCAAGCTTGCCATTGGGATGGAACTTAACC	*phaAB* genes cloning in P_{J23119}
KZ-*phaC*-S	GGCGGCGGCGGCAGCGGCGGCGGCGGCAGCATGAGCAACAAAAACAGCG	*phaC* gene cloning in P_{J23119}
KZ-*phaC*-A	TTCTACTTTTCTCATGGTACCTTTCTCCTCTTTTTAGCGTTCATG	*phaC* gene cloning in P_{J23119}
KZ-*pCT*-S	TAAAAAGAGGAGAAAGGTACCATGAGAAAAGTAGAAATCATTAC	*pCT* gene cloning in P_{J23119}
KZ-*pCT*-A	TTACTCGAGTGCGGCCGCAAGCTTTTATTTTTTCAGTCCCATGGG	*pCT* gene cloning in P_{J23119}
KZ-*rpoS*-S	AAGCAAGCTGATTAAGTCGACAAAGAGGAGAAAACGCGTATGTTCCGTCAAGGGATCACG	*rpoS* gene cloning in P_{mrkA}
KZ-*rpoS*-A	GTGGTGGTGGTGGTGCTCGAGTTACTCGCGGAACAGCGC	*rpoS* gene cloning in P_{mrkA}
KZ-Kan-F	TACAGGTAGTCTGCATGAAACTATTGCGGAAAGAATTCCA	DNA fragment deletion for *nrdA* gene

	AAAACAGGTACGACATACGTGTAGGCTGGAGCTGCTT	
KZ-Kan-R	TACAAACCGAGCCGTAGGCCGGATAAGGCGTTTACGCCGCATCCGGCATCTCAATACATATGAATATCCTCCTTAG	DNA fragment deletion for *nrdA* gene
YZ-Kan-nrdA-F	ACAATAACCGAATAGAAAACAACC	DNA fragment deletion for *nrdA* gene
YZ-Kan-nrdA-R	CAGGAGCAAGGTGAGATGAC	DNA fragment deletion for *nrdA* gene
KZ-*el222*-T-S	ATGAATTCGAGCTCCGTCGACAAAGAGGAGAAAACGCGTATGG	*el222* gene cloning in P_{tet}
KZ-*el222*-T-A	CGTTTTCTCCTCTTTGTCGACTTAGATTCCGGCTTCGACGG	*el222* gene cloning in P_{tet}
KZ-*mrkH*-T-S	TGAAAGAGGAGAAAACGCGTATGACCGAGGGTACGATCAA	*mrkH* gene cloning in P_{tet}
KZ-*mrkH*-T-A	GCCTGGAGATCCTTACTCGAGAATACGCTTTTTGCGCTTCG	*mrkH* gene cloning in P_{tet}
KZ-*yhjH*-T-S	AAGCTTATATACATACGCGTATGATTCGCCAAGTTATTCAGC	*yhjH* gene cloning in P_{tet}
KZ-*yhjH*-T-A	ATACGCGTTTTCTCCTCTTTCAGTGCCAGAACTGCGGTAT	*yhjH* gene cloning in P_{tet}
KZ-*bphO*-T-S	ATAAAGAGGAGAAAACGCGTATGCCGCTGAGCCGCGATCT	*bphO* gene cloning in P_{tet}
KZ-*bphO*-T-A	CTCGAGTGCGGCCGCAAGCTTTTACACACGGCGGGCCGC	*bphO* gene cloning in P_{tet}
KZ-*bphS*-T-S	ATGAATTCGAGCTCCGTCGACAAAGAGGAGAAAACGCGTATGGCCCGTGGTTGTTTAATG	*bphS* gene cloning in P_{tet}
KZ-*bphS*-T-A	ATACGCGTTTTCTCCTCTTTATGATGGTGATGGTGATGATACT	*bphS* gene cloning in P_{tet}
KZ-*bfp*-M-S	CATTCTTTGACGCCGACTAGTAAAGAGGAGAAAACGCGTATGAGCGAGCTGATTAAGGAGAA	*bfp gene* cloning in P_{mrkA}
KZ-*bfp*-M-A	ACCTGTCAAAGATCTAAGCTTTTAATTGAGCTTGTGCCCCAG	*bfp gene* cloning in P_{mrkA}
KZ-*gfp*-R-S	GACAAAGAGGAGAAAACGCGTATGGTGAGCAAGGGCGAGG	*gfp* gene cloning in pBLrep-v1
KZ-*gfp*-R-A	GCTGCCGCCGCCGCCGCTGCCGCCGCCGCCCTTGTACAGCTCGTCCATGC	*gfp* gene cloning in pBLrep-v1
KZ-*mKate*-I-S	GAATTCAAAGAGGAGAAAACGCGTATGTCAGAATTAATTAAAGAAAATATGCAC	*mKate* genes cloning in pBLind-v1
KZ-*mKate*-I-A	CATGCTGCCGCCGCCGCCGCTGCCGCCGCCGCCACGATGTCCTAATTTCGACG	*mKate* genes cloning in pBLind-v1
KZ-*bgaD-D*-I-S	TCGAATAAATGAATTCTCTAGAAAAGAGGAGAAAACGCGTATGAGCGTTAGCTACGATGGTG	*bgaD-D* genes cloning in pBLind-v1
KZ-*bgaD-D*-I-A	CAGGGTAATAGATCTAAGCTTCGGGGTCACGGTGAAAACG	*bgaD-D* genes cloning in pBLind-v1
KZ-*bgaD-D*-R-S	TATAATTGAATTCTCTAGAAAAGAGGAGAAAACGCGTATGAGCGTTAGCTACGATGGTG	*bgaD-D* genes cloning in pBLrep-v1
KZ-*bgaD-D*-I-A	CAGGGTAATAGATCTAAGCTTCGGGGTCACGGTGAAAACG	*bgaD-D* genes cloning in pBLrep-v1

Supplementary Table 2. Primers used for RT-PCR in this study.

Numbers	Primer	Sequence (5′–3′)
1	NrdA-F	GATGGACACCTTTATCGA
2	NrdA -R	GTACCAGATATTTGCCTTC
3	MKate-F	GCATCAACAGAAACCTTA
4	MKate-R	GCCTCCAACTAATTTCAG
5	BFP-F	ACCACATATAGATCCAAGA
6	BFP-R	GTCTGTAGTCCACATAGTA
7	OriC-F	GCCCTGTGGATAACAAGGAT
8	OriC-R	CCTCATTCTGATCCCAGCTT
9	Ter-F	TCCTCGCTGTTTGTCATCTT
10	Ter-R	GGTCTTGCTCGAATCCCTT
11	16S sRNA-F	GCTACAATGGCGCATACAAA
12	16S sRNA-R	TTCATGGAGTCGAGTTGCAG

Supplementary Note 1. The role of 12 genes of in *E. coli*

***ftsZ*[1]:** A cell-division determinant and bacterial tubulin homolog that polymerizes into filaments, which is assembled into the Z ring at the future division site. It provides a scaffold for the assembly of the entire divisome, forming the Z ring under the membrane at the center of the cell.

***ftsA*[1]:** An actin-like protein associated with the membrane through an amphi-pathic helix to stabilize the Z-ring. Owing to the oligomerization state of FtsA, it has been suggested as a critical step in divisome assembly. one would expect that the ATPase cycle of FtsA would be critical for its function.

***ftsQ*[1]:** A bitopic membrane proteins that form a complex, acting as a scaffold for the recruitment of downstream divisome proteins, and a part of the divisome. FtsQ, FtsL and FtsB form a subcomplex even before they localize to the Z ring.

***ftsN*[1]:** It promotes premature interaction between FtsN and FtsA and back recruits the divisome proteins to the Z ring. FtsN is the last recruit during divisome assembly, and there must be another division protein that is earlier than FtsN and interacts with monomeric FtsA.

***nrdA*[2]:** An ribonucleoside-diphosphate reductase 1 subunit alpha for intracellular deoxynucleoside triphosphate synthesis.

***nrdB*[2]:** An ribonucleoside-diphosphate reductase 1 subunit beta for intracellular deoxynucleoside triphosphate synthesis.

***nrdD*[2]:** An anaerobic ribonucleoside-triphosphate reductase for intracellular deoxynucleoside triphosphate synthesis.

***sulA*[3]:** A cell division inhibitor, blocking binary division, turning the normally rod-shape *E. coli* into filamentary cells. Induction of the SulA leads to cell division inhibition by preventing the assembly of nascent Z rings, facilitating the disassembly of existing Z rings, and preventing FtsZ localization into the ring structure.

***minC*[3]:** A septum site-determining protein consists of 231 amino acids, and its N-terminal contains FtsZ binding domain, which directly interacts with FtsZ protein and inhibits the polymerization of FtsZ protein.

***minD*[3]:** A septum site-determining protein consists of 270 amino acids, which can suppress the cell division and prevent the formation of cell division septum, and bind to ATP complex binds to the membrane.

***minE*[3]:** A cell division topological specificity factor consists of 88 amino acids, which can suppress the cell division. It forms a spiral loop in the cell, moving from one pole to the other, and drives the MinCD complex between the two poles.

***ftsH*[3]:** An cell division protease, ATP-dependent zinc metalloprotease, and integral cytoplasmic membrane protein spanning the membrane twice. It has a large cytoplasmic carboxy-terminal part with a putative ATP-binding domain.

Supplementary Note 2. The weak expression of *nrdA* and overexpression of *nrdA* in the C period of cell division.

By analyzing the relative gene expression levels, the *nrdA* expression levels in the Δ*nrdA* mutant was decreased by 94%, compared to that of wild type (Supplementary Fig. 3C and Supplementary Fig. 12B). Thus, the C period of Δ*nrdA* mutant was increased by 240.99% up to 120.2 min, compared to that of wild type (Supplementary Fig. 3C).

The *nrdA* expression levels in the Δ*nrdA* mutant was decreased by 76%, compared to that of wild type (Supplementary Fig. 11A, Supplementary Fig. 12B). Thus, the weak expression of *nrdA* in the Δ*nrdA* mutant led to a 124.6% increase in the C period up to 9.17 min (Fig. 2A).

The *nrdA* expression levels in *E.coli* DQ181 was increased by 3.88-fold, compared to that of wild type (Supplementary Fig. 11A, Supplementary Fig. 12A).The overexpresssion of *nrdA* in wild type led to a 29.15% decrease in the C period up to 25.79 min (Fig. 1A).

Supplementary Note 3. The two phases fermentation for acetoin production

Two phases fermentation was carried out for acetoin production. In phase I*, the *nrdAB* and *ftsZA* genes were expressed at moderate level for cell growth from 0-12 h. In phase II*, the *rpoS* gene was expressed at high level and the *nrdAB* and *ftsZA* genes were expressed at moderate level for defensing acetoin stress from 12-72 h. The final results showed that the acetoin titer, productivity, $OD_{600,}$ and cell growth rate were increased to 59.88 $g·L^{−1}$, 0.83 $g·L^{−1}·h^{−1}$, 57.49, and 2.82 $h^{−1}$, but were decreased by 10.89%, 10.89%, 11.51%, and 20.34% compared to that of three phases fermentation, respectively (Supplementary Date 4). Overall, these results indicated that phase II was an important phase that might not be skipped from phase I into phase III.

Supplementary Note 4. Effect of light penetration on fluorescence expression in 5-L and 10-L fermenters

In 5-L fermenter, our supplementary experiments showed that mKate and BFP expression could be activated at cell density OD_{600} = 80. The fluorescence intensity of mKate and BFP was increased to 3588.31 a,u. and 3358.32 a,u., which was decreased by 3.34% and 9.6%, compared to that of 250 mL shake flasks, respectively. In addition, the fluorescence density percentage of mKate and BFP was increased to 99.8% and 99.7%, which was similar to that of 250 mL shake flasks, respectively (Supplementary Fig. 31, Supplementary Fig. 32C-H, and Supplementary Fig. 33C-H). Overall, our optogenetics regulation strategy exhibited the fine suitability in the process of fermentation in 5-L fermenter.

In 10-L fermenter, our supplementary experiments showed that mKate and BFP expression could be activated at cell density OD_{600} = 90. The fluorescence intensity of mKate and BFP was increased to 3493.47 a,u. and 3065.65 a,u.,which was decreased by 2.64% and 8.71%, compared to that of 5-L fermenter, respectively. In addition, the fluorescence density percentage of mKate and BFP was increased to 91.3% and 90.6%, which was decreased by 8.52% and 9.13% , respectively (Supplementary Fig. 31C, Supplementary Fig. 32C-J, and Supplementary Fig. 33C-J). Overall, our optogenetics regulation strategy exhibited the fine suitability in the process of fermentation in 10-L fermenter.

To sum up, the above results indicated that optogenetics tools possessed the fine suitability and applicability from 250 mL shake flasks to 5-L fermenter, and then to 10-L fermenter. These optogenetics tools could meet the need of metabolic engineering and the process of fermentation production.

Supplementary Note 5. Effect of IPTG concentration on gene expression

For investigating the effect of IPTG concentration on gene expression, we used IPTG to induce the expression of key genes in cell division. With the increase of IPTG concentration, the relative expression levels of mKate were increased from 0.05 to 1. In addition, the mKate/OD was increased from 6.25% to 100% (Supplementary Fig. 10A, B). Overexpression of *nrdAB* and *ftsZA* genes could efficiently shorten the C and D periods to 21.93 and 17.95 min, respectively. In addition, the weak *nrdA* expression and *sulA* overexpression could efficiently prolong the C and D periods to 79.31 and 127.21 min, respectively (Fig. 1A, C); These results showed that the gene expression in the C and D periods of cell division could be efficiently controlled by different IPTG concentration.

Supplementary Note 6. Illumination intensity experiments for acetoin production

The complete blue light and NIR light illumination intensity experiments in three phases were carried out: **(i)** blue light illumination intensity in three phases was optimized under three different levels: high (H, 0.8 W/cm^2), moderate (M, 0.3 W/cm^2), and low (L, 0.2 W/cm^2) (Supplementary Fig. 30A). The genes in the C+D periods of cell division were controlled by blue light in *E. coli* D1, resulting in *E. coli* DB5 (Blue-H-M-M represented 0.8 W/cm^2 blue-light in phase I and 0.3 W/cm^2 blue-light in phase II and phase III). The results showed that *E. coli* DB5 produced the highest acetoin titer up to 25.6 $g{\cdot}L^{-1}$, which exhibited a 89.63% improvement compared to that of the control strain *E. coli* D1. Further, the cell growth (OD_{600}) of *E. coli* DB5 was up to 16.8, which was 43.34% higher than that of the *E. coli* D1; **(ii)** NIR light illumination intensity in two phases (phase II and III) was optimized under three different levels (Supplementary Fig. 30B). The *rpoS* gene was controlled by NIR-light in *E. coli* DB5, resulting in *E. coli* DN4. The results showed that *E. coli* DN4 produced the highest acetoin titer up to 31.2 $g{\cdot}L^{-1}$, which exhibited a 131.1% improvements compared to that of the control. Further, the cell growth (OD_{600}) of *E. coli* DN4 was up to 21.2 (OD_{600}), which was 80.89% higher than that of the control strain *E. coli* D1. These results showed that the acetoin titer could be improved by optimizing blue light and NIR light illumination intensity.

Supplementary References

1. Du, S. & Lutkenhaus, J. Assembly and activation of the *Escherichia coli* divisome. *Mol Microbiol* **105**, 177-187 (2017).
2. Zhu, M.l. et al. Manipulating the bacterial cell cycle and cell size by titrating the expression of ribonucleotide reductase. *Mbio* **16**, 38-51 (2017).
3. Jiang, X.R. & Chen, G.Q. Morphology engineering of bacteria for bio-production. *Biotechnol Adv* **34**, 435-440 (2016).
