# Supplementary material for: Light-powered Escherichia coli cell division for chemical production
Source: Nat Commun. 2020 May 8;11:2262. doi: 10.1038/s41467-020-16154-3 (PMC7210317; doi:10.1038/s41467-020-16154-3)
Supplement: Supplementary file 3 — Description of Additional Supplementary Files [file 41467_2020_16154_MOESM3_ESM.docx]

**Description of Additional Supplementary Files**

File name: Supplementary Data 1
Description: Plasmids. The relative plasmids are constructed in this study.

File name: Supplementary Data 2
Description: Strains. The relative plasmids are constructed in this study.

Supplementary Data 3
Description: DNA sequences. DNA sequences for plasmid genetic parts, optogenetics part, reporter protein, and pathway enzymes.

File name: Supplementary Data 4
Description: Acetoin production. Different phases regulation for acetoin production.
